# Novel 1,2,4-Triazole- and Tetrazole-Containing 4*H*-Thiopyrano[2,3-*b*]quinolines: Synthesis Based on the Thio-Michael/aza-Morita–Baylis–Hillman Tandem Reaction and Investigation of Antiviral Activity

**DOI:** 10.3390/molecules28217427

**Published:** 2023-11-04

**Authors:** Andrey V. Khramchikhin, Mariya A. Skryl’nikova, Maxim A. Gureev, Vladimir V. Zarubaev, Iana L. Esaulkova, Polina A. Ilyina, Oussama Abdelhamid Mammeri, Dar’ya V. Spiridonova, Yuri B. Porozov, Vladimir A. Ostrovskii

**Affiliations:** 1 Department of Chemistry and Technology of Organic Nitrogen Compounds, Saint Petersburg State Institute of Technology (Technical University), 26 Moskovsky Avenue, 190013 St. Petersburg, Russia; xram62@mail.ru (A.V.K.); mari954@mail.ru (M.A.S.); 2 Saint Petersburg Federal Research Center of the Russian Academy of Sciences (SPC RAS), 39, 14th Line, 199178 St. Petersburg, Russia; 3 Center of Bio- and Chemoinformatics, I. M. Sechenov First Moscow State Medical University, 8, Trubetskaya Street, Bld. 2, 119991 Moscow, Russia; max_technik@mail.ru (M.A.G.); yuri.porozov@gmail.com (Y.B.P.); 4 St. Petersburg School of Physics, Mathematics and Computer Science, HSE University, 16, Soyuza Pechatnikov Str., 190008 St. Petersburg , Russia; 5 Saint Petersburg Pasteur Research Institute of Epidemiology and Microbiology, 14 Mira Street, 197101 St. Petersburg, Russia; zarubaev@gmail.com (V.V.Z.); esaulkova@pasteurorg.ru (I.L.E.); il401polina@gmail.com (P.A.I.); 6 Chemical Analysis and Materials Research Center, St. Petersburg State University, 26, Universitetskii Prospect, Petergof, 198504 St. Petersburg, Russia; oussama.mammeri@mail.ru; 7 Research Park, St. Petersburg State University, 26, Universitetskaya Emb. 7/9, 199034 St. Petersburg, Russia; daria.spiridonova@spbu.ru

**Keywords:** thiopyrano[2,3-*b*]quinolines, tetrazole, 1,2,4-triazole, 3-phenyl-2-propynal, antiviral activity, molecular docking, influenza virus A/Puerto Rico/8/34

## Abstract

A novel method for synthesizing 1,2,4-triazole- and tetrazole-containing 4*H*-thiopyrano[2,3-*b*]quinolines using a new combination of the thio-Michael and aza-Morita–Baylis–Hillman reactions was developed. Target compounds were evaluated for their cytotoxicities and antiviral activities against influenza A/Puerto Rico/8/34 virus in MDCK cells. The compounds showed low toxicity and some exhibited moderate antiviral activity. Molecular docking identified the M2 channel and polymerase basic protein 2 as potential targets. We observed that the antiviral activity of thiopyrano[2,3-*b*]quinolines is notably affected by both the nature and position of the substituent within the tetrazole ring, as well as the substituent within the benzene moiety of quinoline. These findings contribute to the further search for new antiviral agents against influenza A viruses among derivatives of thiopyrano[2,3-*b*]quinoline.

## 1. Introduction

In recent decades, a significant increase in the number of pandemic-prone viral infections has been registered, posing serious threats to public health [1]. The ability of viruses to evolve and develop resistance to existing antiviral drugs presents an urgent task for the scientific community to search for new and effective active pharmaceutical ingredients [2].

**Figure 1:**
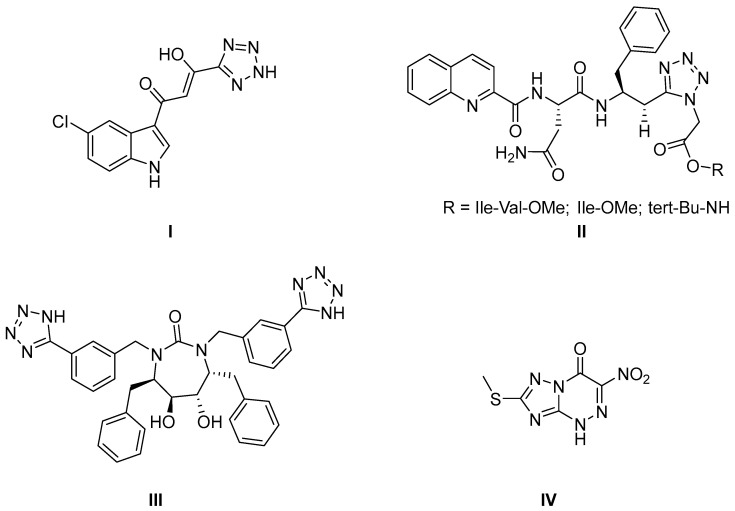
Triazole-, tetrazole-, and quinoline-containing active pharmaceutical ingredients exhibiting antiviral activity: I—viral integrase inhibitor; II, III—HIV protease inhibitors; IV—an inhibitor of viral RNA replication.

In this context, the search for new compounds capable of exhibiting antiviral activity plays an important role. Specifically, triazolyl, tetrazolyl, and quinoline fragments have attracted the attention of researchers as promising scaffolds for the development of antiviral drugs (Figure 1) [3,4,5,6]. These fragments represent prospective building blocks for the creation of new medicine.

It is important to note that the introduction of a sulfur atom into the molecular structure of active pharmaceutical ingredients is also a promising approach that allows for the modulation of antiviral activity. The sulfur atom can play a crucial role in the interaction of compounds with biological targets and enhance their pharmacological effectiveness [7], as illustrated by antiviral drugs with a nitrogen-containing heterocycle and an exo- or endo-cyclic sulfur, such as triazavirine, baloxavir marboxil, and umifenovir [2,8,9,10,11].

One of the promising biologically active compounds of this type is thiopyrano[2,3-b]quinolines. While the corpus of knowledge concerning their biological activity remains constrained, it is pertinent to acknowledge their potential to manifest substantial biological efficacy, notably in terms of antitumor properties [12]. Furthermore, they have demonstrated remarkable attributes encompassing antimicrobial [13], antioxidant activity, and modulation of the mGlu 1 receptor [14].

In this article, we present the results of an experimental study dedicated to the development of new approaches for the synthesis of 1,2,4-triazole- and tetrazole-containing 4*H*-thiopyrano[2,3-b]quinolines based on the tandem thio-Michael/aza-Morita–Baylis–Hillman reaction. Furthermore, the in vitro activity against influenza A/Puerto Rico/8/34 (H1N1) virus was investigated. To interpret the obtained data, molecular docking and scoring were performed using the Schrödinger Suite 2022-4 platform.

## 2. Results and Discussion

### 2.1. Chemistry

Successful development of new drugs requires experimental testing of a large number of compounds [15]. Therefore, to optimize the synthesis process, it is necessary to use a minimal number of starting compounds to obtain a broad spectrum of substances [16]. In this context, acetylenic aldehydes are of particular interest due to their high reactivity, which results from the presence of an activated triple bond and a carbonyl group. As a second component, we used 2-mercaptoquinolin-3-carbaldehyde, which possesses two reactive centers—mercapto and carbonyl groups. In the investigated reactions, this compound can be used both as an independent substrate and in the form of derivatives, such as azomethines. Such an approach enables further studying, for the generation of a diverse set of compounds, using a limited number of key reagents.

The modern approaches to the development of new synthetic methods are based on the search for reactions that eliminate the use of expensive, toxic, and hazardous reagents, while allowing the synthesis of a wide range of diverse substituted organic compounds with high yields in a minimal number of steps, with minimal byproducts [17]. Numerous studies conducted in recent decades demonstrate that atom-economical reactions best meet these requirements as all atoms of the reactants are retained in the products. Moreover, reactions such as an addition to double and triple carbon bonds often serve as a basis for the development of convenient synthesis methods for compounds that are otherwise challenging to obtain through alternative means. A significant number of tandem reactions belong to this category of atom-economical reactions [18].

Tandem reactions are powerful tools in organic synthesis, enabling the efficient synthesis of complex molecules. At the core of tandem reactions is the sequential conducting of two or more chemical transformations within a single reaction system [19]. They allow for the rational use of reaction intermediates generated in the first step to directly conduct the second step of the reaction without isolating intermediate products. One promising tandem reaction used in the synthesis of biologically active compounds is the combination of the Michael reaction with the Morita–Baylis–Hillman reaction [20]. The Michael reaction, based on the nucleophilic attack on the triple bond of acetylenic carbonyl compounds, can be combined with the Morita–Baylis–Hillman reaction, which yields an adduct containing a double bond and a carbonyl group [21]. Such reactions enable the efficient synthesis of complex molecules, including tetrazolyl and triazolyl derivatives of thiopyranoquinolines, which exhibit potential antiviral activity. Moreover, they provide a unique opportunity to create combinations of diverse chemical structures and functional groups, opening up new prospects in the development of biologically active compounds.

**Scheme 1:**
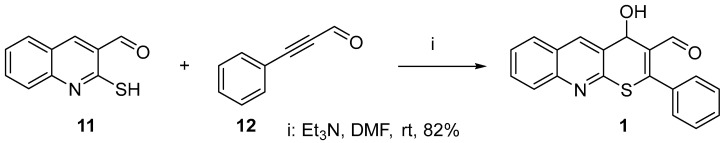
Synthesis of 4-hydroxy-2-phenyl-4*H*-thiopyrano[2,3-b]quinoline-3-carbaldehyde **1**.

The reaction between 2-mercaptoquinoline-3-carbaldehyde and the key annulation reagent, 3-phenyl-2-propynal, was used as our model system. We demonstrated, for the first time, that the interaction of these reagents proceeds through a tandem reaction pathway involving the thio-Michael/Morita–Baylis–Hillman reaction, resulting in the formation of 4-hydroxy-2-phenyl-4*H*-thiopyrano[2,3-b]quinoline-3-carbaldehyde **1** (Scheme 1) [22]. Usually, in both intermolecular and intramolecular Morita–Baylis–Hillman reactions, relatively expensive tertiary amines such as DABCO, DBU, and tertiary phosphines are used as catalysts and these reactions can endure for extended durations, spanning hours or even weeks [20]. However, triethylamine, which is considerably more affordable, is reported in the literature sources to be ineffective in Morita–Baylis–Hillman reactions [23,24]. In our initial attempts to bond a thiol group of 2-mercaptoquinolin-3-carbaldehyde **11** to the triple bond of 3-phenyl-2-propynal **12**, we unexpectedly discovered that triethylamine not only catalyzed the thio-Michael addition but also efficiently catalyzed the subsequent Morita–Baylis–Hillman reaction. It is noteworthy that the reaction with triethylamine proceeded in DMF at room temperature within a few minutes. The resulting cyclic adduct **1** precipitated and, after cooling and filtration, did not require further purification. The yield was 82%.

**Scheme 2:**
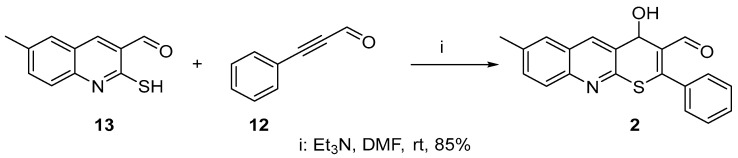
Synthesis of 4-hydroxy-7-methyl-2-phenyl-4*H*-thiopyrano[2,3-b]quinoline-3-carbaldehyde **2**.

In the present article, we present the synthesis data of a homologue of compound **1**—4-hydroxy-7-methyl-2-phenyl-4*H*-thiopyrano[2,3-b]quinoline-3-carbaldehyde **2** (Scheme 2).

**Scheme 3:**
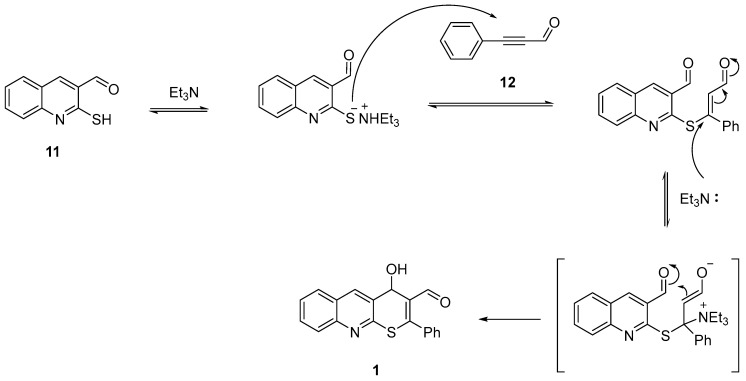
The potential mechanistic pathway of the tandem reaction.

The potential mechanistic pathway of the tandem reaction (Scheme 3), illustrated by the synthesis of compound **1**, commences with the initial attachment of the sulfur anion of 2-mercaptoquinolin-3-carbaldehyde **11** to the triple bond of 3-phenyl-2-propynal **12** through a Michael-type reaction. Subsequently, triethylamine, functioning as a nucleophile, engages in an attack on the activated double bond of the intermediate compound, forming a zwitterionic substance. In this sequence, a conventional electrophilic center takes on the role of a nucleophilic center, participating in an attack on the carbonyl group. The elimination of triethylamine leads to the formation of the desired 4*H*-thiopyrano[2,3-b]quinoline product.

**Scheme 4:**
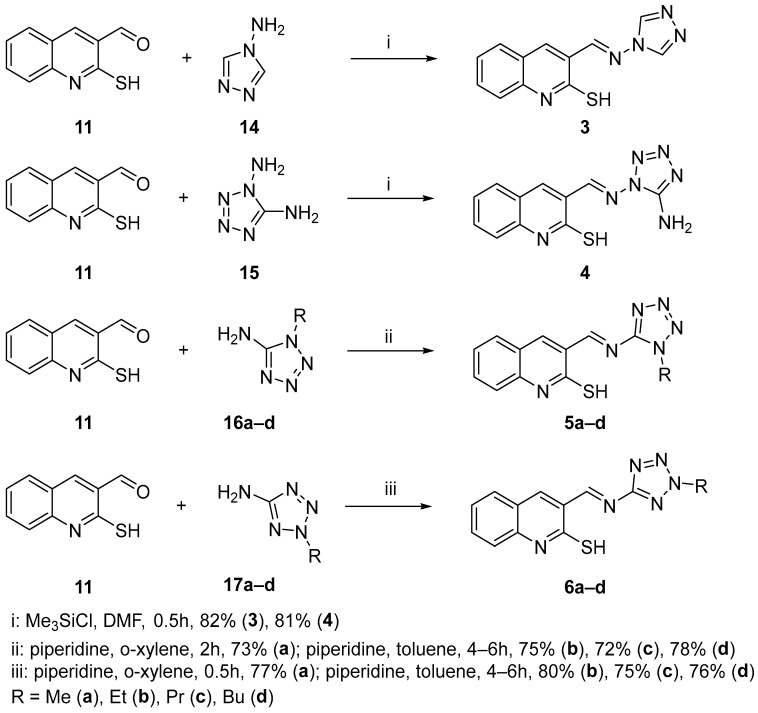
Synthesis of azomethines **3**, **4**, **5a–d**, and **6a–d**.

In our further experiments, we explored the thio-Michael/aza-Morita–Baylis–Hillman tandem reaction by using azomethines **3**, **4**, **5a–d**, and **6a–d** as substrates. These azomethines were derived from 2-mercaptoquinolin-3-carbaldehyde **11** and various heterocyclic amines **14**, **15**, **16a–d**, and **17a–d** (Scheme 4). The azomethines, when using 4*H*-1,2,4-triazol-4-amine **14** and 1*H*-tetrazole-1,5-diamine **15**, were synthesized in the presence of trimethylchlorosilane as a catalyst in DMF following the procedure [25]. When 1-alkyl and 2-alkyl-5-aminotetrazoles **16a–d** and **17a–d** were used, the reaction was carried out in boiling xylene or toluene with piperidine as the catalyst, using a Dean–Stark trap [26].

**Scheme 5:**
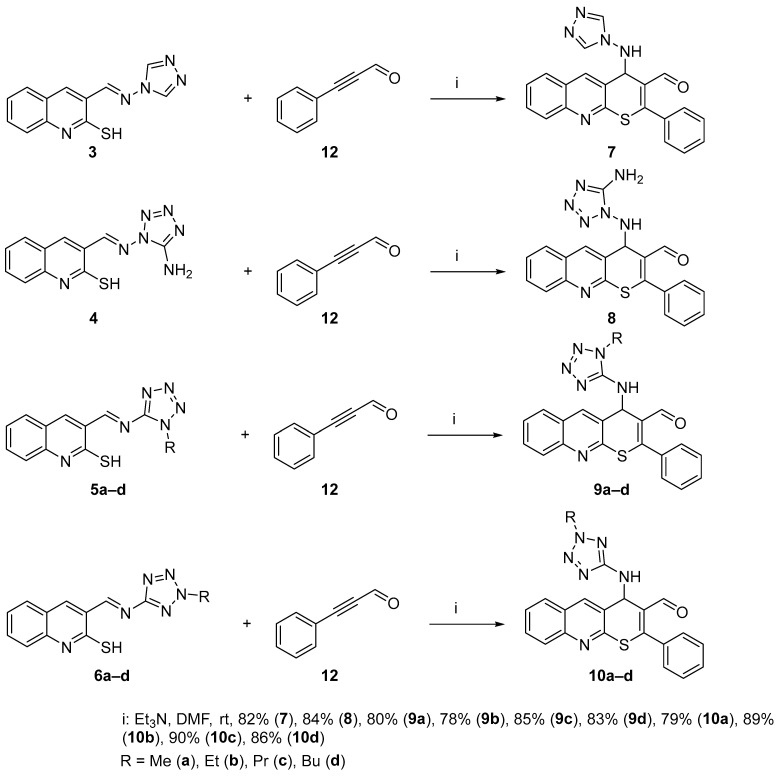
Synthesis of thiopyrano[2,3-b]quinolines **7**, **8**, **9a–d**, and **10a–d**.

This enabled us to significantly increase both the quantity and diversity of target compounds and incorporate pharmacophoric 1,2,4-triazolyl and tetrazolyl fragments into their structures. Similar to compounds **1** and **2**, the thio-Michael/aza-Morita–Baylis–Hillman tandem reaction using azomethines **3**, **4**, **5a–d**, **6a–d**, and 3-phenyl-2-propynal proceeded rapidly with minimal polymerization of starting materials and high yields. No byproducts were detected, which is notable since tandem reactions very often involve their formation [27]. The resulting cycloadducts did not require additional purification following isolation (Scheme 5).

**Figure 2:**
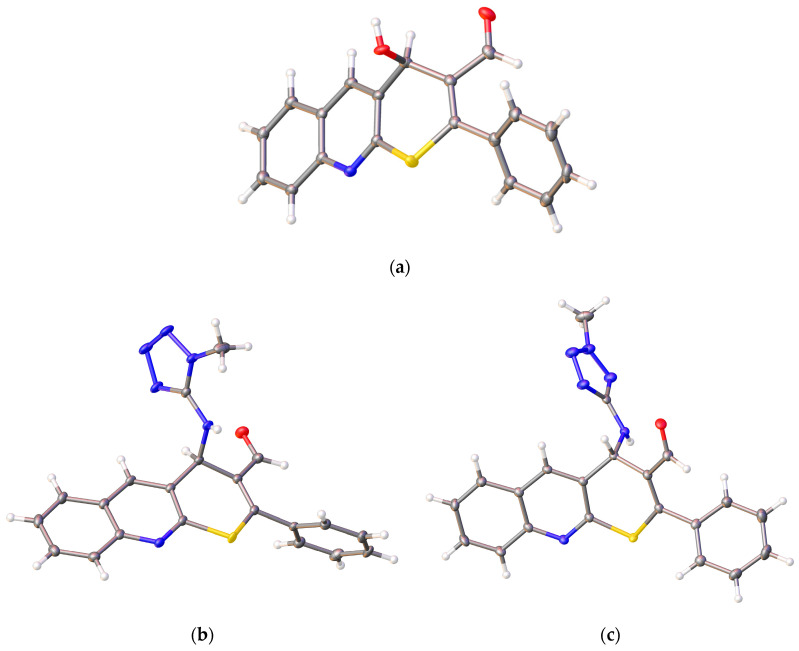
Structures of the compounds: (**a**) **1**; (**b**) **9a**; and (**c**) **10a**.

It is also worth noting the high potential of the obtained thiopyrano[2,3-b]quinolines, which contain a highly reactive carbonyl group and an activated double bond, for further chemical transformations. The structures of the compounds were confirmed by NMR spectroscopy, IR spectroscopy, X-ray crystallography (XRD), and the elemental composition was confirmed by high-resolution mass spectrometry. The structure of compounds **1** (CCDC 2289287), **9a** (CCDC 2271109), and **10a** (CCDC 2271110) is presented in Figure 2.

### 2.2. In Vitro Experiments and Target Validation

We initially conducted a biological activity prediction for the synthesized compounds using the latest version (2022) of the PASS (Prediction of Activity Spectra for Substances) online web resource [28,29]. The first attempt to establish a correlation between the molecular structure and its activity revealed a low probability of exhibiting antiviral activity. For instance, compound **10c** exhibited the highest *Pa* value (estimated probability of activity) among the target compounds, with a value of only 0.37, whereas satisfactory *Pa* values should exceed 0.7 [30]. According to PASS developers, this result indicates the absence of similar compounds in the database, highlighting the novelty of our research [30]. Consequently, we did not stop at the achieved outcome but continued our investigations towards in vitro tests against the influenza virus A/Puerto Rico/8/34 and employed contemporary molecular docking tools to interpret the obtained data.

The cytotoxic and antiviral properties of the synthesized compounds **1**, **2**, **7**, **8**, **9a–d**, and **10a–d** were tested in vitro against the influenza A/Puerto Rico/8/34 virus in MDCK cells. In general, the investigated compounds were characterized by low toxicity. CC_50_ values within the studied concentration range (3.7–300 μg/mL) were observed for only 2 (**10a** and **10d**) out of the 12 examined compounds. The remaining compounds showed no cytotoxicity even at the maximum concentrations used (300 μg/mL). The compound **10d**, which had a butyl substituent at the second nitrogen atom of the tetrazole ring, was the most toxic, highlighting the significance of both the length and position of the aliphatic group for the compound’s cytotoxicity.

The antiviral properties of the investigated compounds were generally weak. Out of the 12 compounds, only three (**1**, **9a**, and **10c**) exhibited selectivity indices above 10 (Table 1). Analysis of the structure–activity relationship revealed that compound **1** showed the highest activity among the compounds, with a hydroxyl group at the 4-position of the thiopyrano ring. The introduction of a methyl group at the 6-position of the scaffold led to a loss of activity (compounds **1** and **2**, SI = 31 and >3, respectively). Among the derivatives with 4-azolamino substitutions, compound **10c**, containing a propyl group at the second nitrogen atom of the tetrazole ring, exhibited the highest activity (IC_50_ = 18.4 µM, SI > 38). Compound **9a**, containing a methyl group at the first nitrogen atom of the tetrazole ring, showed slightly higher activity (IC_50_ = 46 µM, SI > 16). Compound **7**, with a triazole substituent, also demonstrated a certain level of selectivity (SI = 5). Other modifications of the azole substituents, including the introduction of aliphatic groups, elongation of their chains, and their relocation from one position to another, completely eliminated the antiviral activity of the compounds.

According to experimental data, some compounds have shown higher antiviral activity than the standard, rimantadine, but not enough to recommend them for preclinical studies. Nevertheless, in our view, compounds in this series may exhibit significantly greater antiviral activity, and it is currently important to define the strategy for searching for new active structures within this group of compounds. To justify the direction of further research, we have leveraged the capabilities of computer modeling, including modern approaches and resources. We believe that, at this stage, such research is reasonable and will be beneficial to a wide range of researchers.

The following targets (and corresponding PDB models) were considered: HA (hemagglutinin, 1RU7 [31]), NP (nucleoprotein, 5TJW [32]), PB2 (polymerase basic protein 2, 4U6O), and PA (polymerase acidic protein, 5FDG [33]) (Table 2).

All protein structures are from influenza A virus subtype A/H1N1/Puerto Rico/8/34 and were obtained from the RCSB Protein Data Bank.

None of the molecules were able to form an energetically favorable ligand–protein complex with hemagglutinin at the camphecene-binding site [34].

Regarding neuraminidase (NA), all compounds showed a low level of predicted affinity. The clustering of docking solutions was also low, indicating a low probability of site-specific interaction. A similar situation was observed with nucleoprotein (NP).

Therefore, the most interesting targets are the M2 channel, where the investigated compounds bind with relatively high-scoring function values comparable to the reference and the PB2 protein, which is also included in the pool of potential targets as its scoring function profile correlates with the IC_50_ values.

Additionally, some structures (compounds **9c** and **9d**) exhibit selective but weak interaction with the polymerase protein PA.

**Figure 3:**
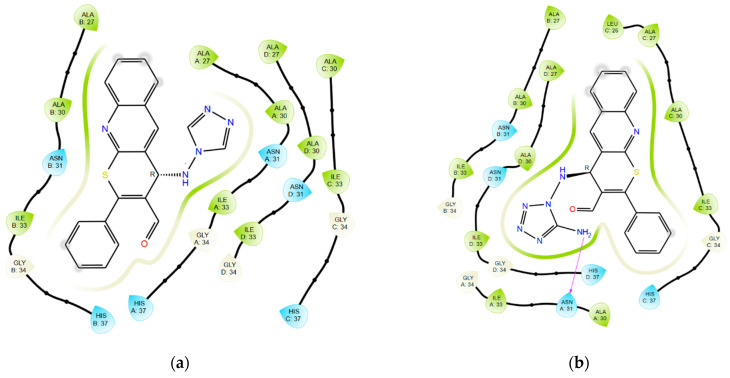

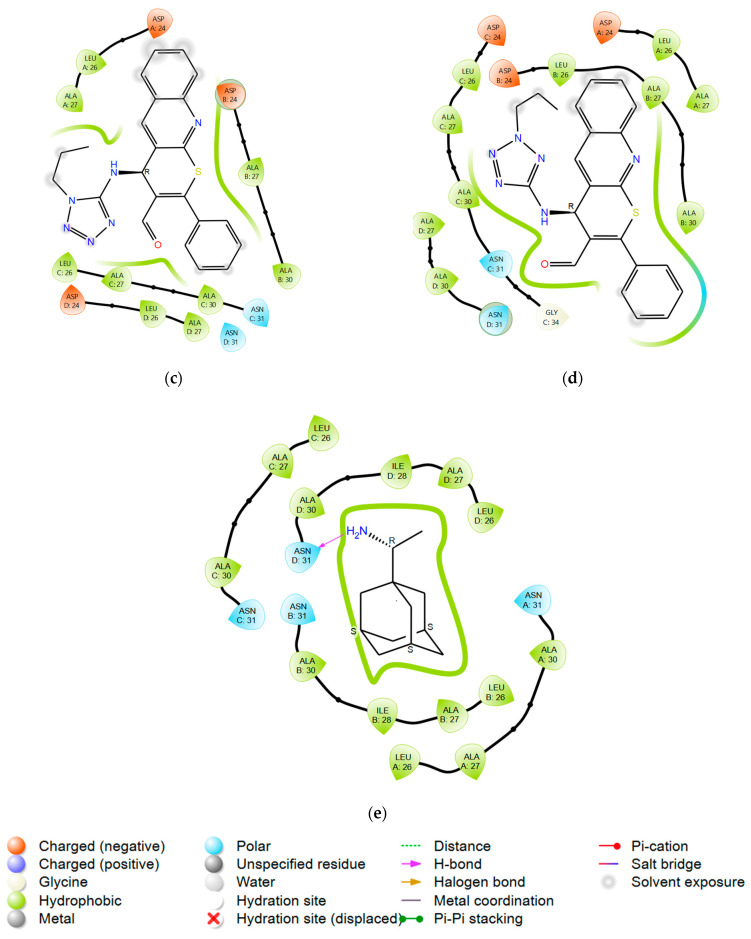
Ligand interaction diagrams for compounds: (**a**) **7**; (**b**) **8**; (**c**) **9c**; (**d**) **10c**; and (**e**) **rimantadine** with M2 channel.

**Figure 4:**
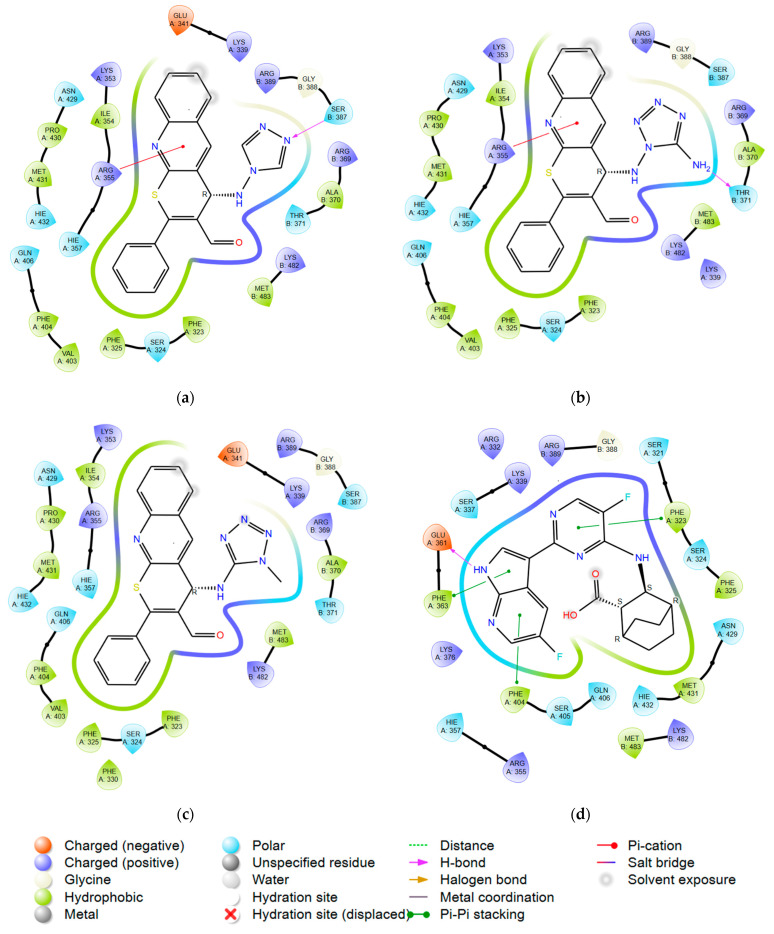
Ligand interaction diagrams for compounds: (**a**) **7**; (**b**) **8**; (**c**) **9a**; and (**d**) native control from the 4U6O pdb model with PB2 protein.

Structures with optimal scoring function values exhibit a high degree of clustering of docking solutions and reproduce key interactions observed in control structures. Detailed interactions are shown in Figure 3 and Figure 4.

Compounds **10b**–**d** have weak predicted affinity to the M2 channel and PB2 (based on the scoring function values in Table 2, values less than for the control compounds). However, on the basis of experimental data, compounds **10c** and **10d** display relatively high IC_50_ values. This phenomenon may be explained by a synergistic effect achieved by weak but notable interaction with two different targets that engage distinct molecular mechanisms, leading to the suppression of viral activity.

Compound **10c**, which is an isomer of **9c**, demonstrates higher activity due to more stable hydrophobic contacts with the M2 channel. The propyl substituent is oriented towards the leucine residues Leu26 of chains B and C, as well as Ala30, providing a more favorable binding within the M2 channel cavity.

As it can be seen from the results (Table 2) that the values of IC_50_ do not correspond to the values of docking score. This can be explained by the differing bioavailability of compounds, which is important for in vitro results where live cells are used, and is not taken into account in the course of molecular modeling. In particular, compounds may be of low stability or low ability to cross the cellular membrane due to their physicochemical properties. Alternatively, limitations of the methods of modeling we used may play a role.

Binding poses of observed compounds in PB2 active pocket for compounds **7**, **8**, and **9a** and native control from the 4U6O pdb model are presented in the Supplementary Material (Figure S52).

Compounds **7**, **8**, and **9a**, with remarkable affinity to PB2, showed the presence of principal interactions similar to the control compound (Figure 4d). The crucial interactions are: lipophilic interactions with Phe404/325/323, charged Arg355, and Lys376 [35]. Heterocyclic sulfur gives additional electron density, stimulating protein–ligand binding affinity.

In the case of the M2 channel, there is a pronounced affinity gradient that correlates with the IC_50_ values (Table 3). The key binding factor of the investigated compounds is the hydrophobic interactions realized within the active site of the M2 channel. The energy of these interactions slightly increases with the IC_50_ values. However, there is no significant change observed that correlates with the level of activity.

**Figure 5:**
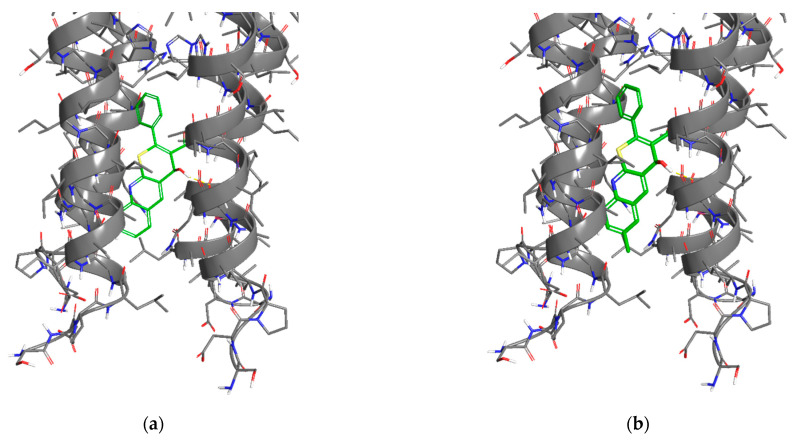

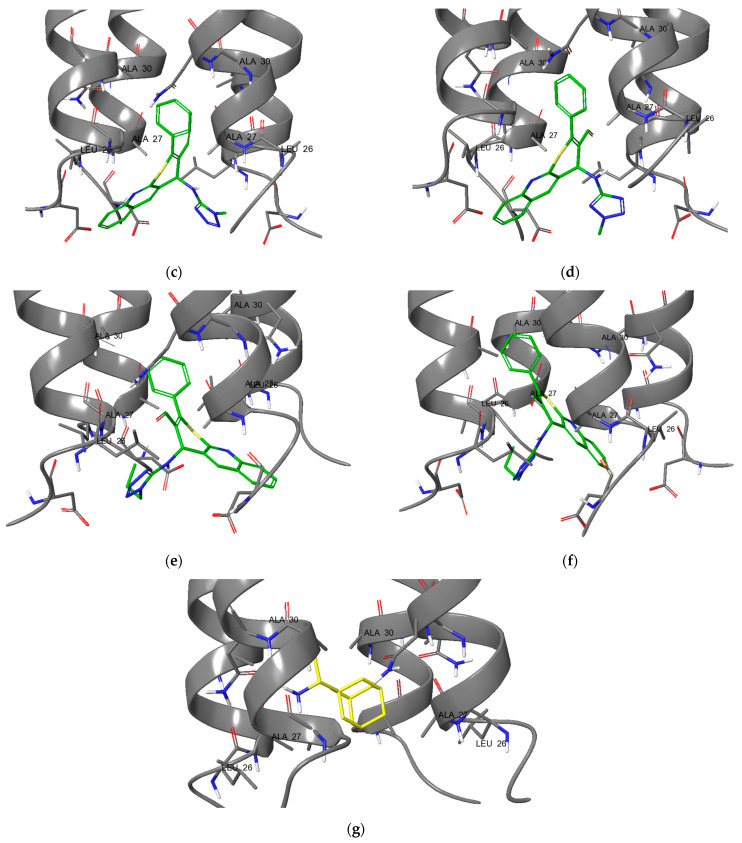
Protein–ligand complexes of compounds: (**a**) **1**; (**b**) **2**; (**c**) **9a**; (**d**) **10a**; (**e**) **9c**; (**f**) **10c**; and (**g**) **rimantadine** with M2 channel.

Analyzing the binding modes of the investigated compounds may provide us with more information about the changes in activity level based on the introduced substituents in the structure (Figure 5).

The analysis of the binding modes revealed that the tetrazole-substituted compounds exhibit different binding modes. All observed compounds interact with the active site of the M2 channel in a different manner. Compounds **1** and **2** (Figure 5a,b) have similar binding poses and are positioned closely to the histidine catalytic center (His37 in chains A–D) and its quinoline moiety interacts between Ala27/30 of M2 channel. This is similar to rimantadine. The addition of a methyl group to the ring of quinoline in the sixth position leads to the activity loss. This can be explained by the strain energy growth (Table 3), which indicates the presence of strained contacts, which is unfavorable for protein–ligand complex formation and thus scoring is worse. Compounds **9a** and **10a** directly obstruct the channel entrance (Figure 5c,d). The scaffold with phenyl substituent interacts with the hydrophobic core of the receptor: Ala27/30 in chains A–D (similar to rimantadine). However, the less potent compound **10a** has increased strain energy (Table 3), which indicates a lower affinity due to unstable binding (also estimated by GlideScore value). Compounds **9c** and **10c**, on the contrary, demonstrate affinity growth when 1-propyl-1*H*-tetrazole substituent was changed to 2-propyl-2*H*-tetrazole, but overall scoring values showed low target affinity.

**Figure 6:**
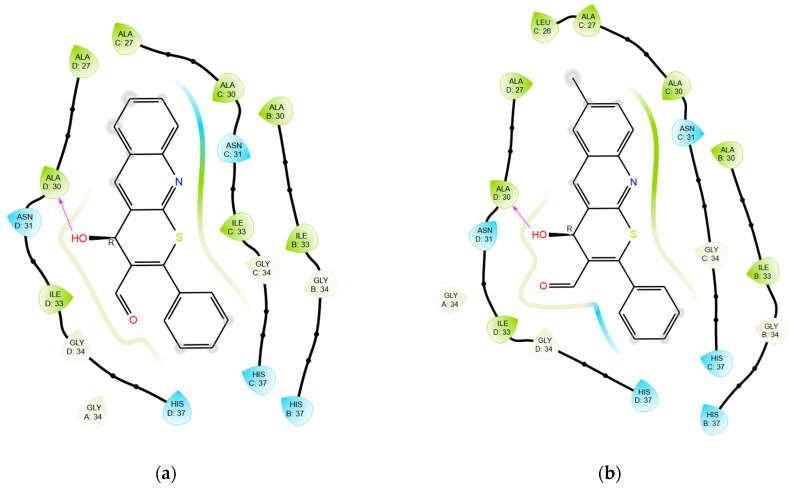

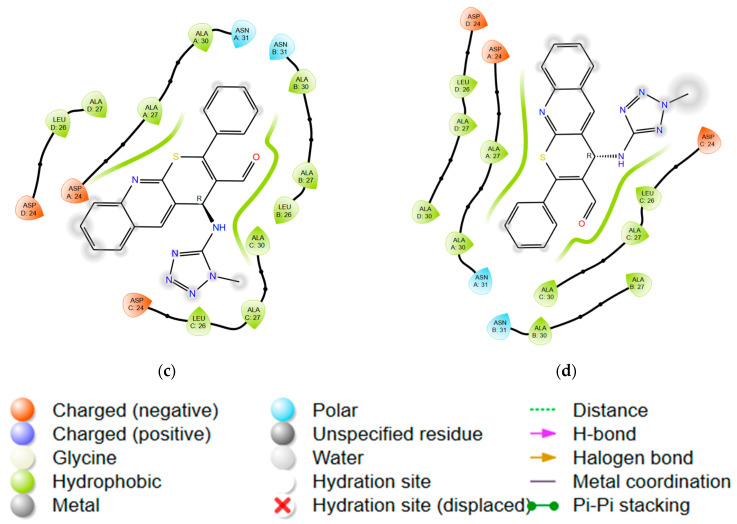
Ligand interactions diagrams for compounds: (**a**) **1**; (**b**) **2**; (**c**) **9a**; and (**d**) **10a** with M2 channel.

The trend of decreasing activity (experimental) associated with the introduction of a methyl substituent (**1** and **2**) or its migration along the nitrogen atoms of the tetrazole ring (**9a** and **10a**) is likely linked to the alteration of the hydrophobic interactions map (Figure 6). This results in steric hindrance during ligand binding to the active site of the M2 channel, caused by early contact with external hydrophobic amino acid sidechains at the active cavity entrance. As a consequence, the binding selectivity to the protein’s active center diminishes.

Thus, the calculations performed on the studied compounds revealed that the M2 channel and polymerase basic protein 2 are the most favorable targets. The interaction with the M2 channel is energetically advantageous, thanks to significant lipophilic interactions, ensuring specific binding, while the binding to PB2 primarily relies on electrostatic interactions facilitated by the aromatic scaffold structure and the electron density of the tetrazole/triazole fragments.

During in vitro studies and computer data interpretation, we discovered that the nature and placement of the substituent in the tetrazole ring, as well as the substituent in the benzene moiety of quinoline, significantly alter antiviral activity of studied thiopyrano[2,3-b]quinolines. Based on this finding, we plan to continue the search for active structures by varying the nature of the mentioned substituents.

## 3. Materials and Methods

### 3.1. General Information

IR spectra were recorded on an IR Affinity-1 Fourier transform spectrometer for studies in the mid-IR range in KBr pellets. ^1^H and ^13^C NMR spectra (400 and 101 MHz, respectively) were acquired on a Bruker Avance III HD 400 NanoBay spectrometer in DMSO-d_6_, using the signals of the deuterated solvent DMSO-d_6_ as internal standard. High resolution mass spectra (electrospray ionization ESI) were performed on a Bruker micrOTOF mass spectrometer. Melting points were determined on a Büchi M-560 apparatus with a heating rate of 1 °C/min in the melting range. Monitoring of the reaction progress was conducted by TLC on Merck Kieselgel 60 F_254_ plates. Analytical grade solvents were used without additional purification. The experimental NMR spectra were processed using the MestReNova software (v. 12.0.0-20080) to remove the signals of water protons present in DMSO-d_6_ in the ^1^H NMR spectra and noise in the ^13^C NMR spectra (an example of ^1^H and ^13^C NMR spectrum processing is shown in the Figures S53 and S54).

### 3.2. Chemistry

Synthesis and analytical data for compounds **1**, **5a**, **6a**, **9a**, and **10a** were described by us in the short communication [22]. Crystals of compounds **1**, **9a** and **10a** were obtained from DMF for RSA.

**General procedure for the synthesis of compounds 3**, **4.**

*N*-Aminoazole **14** or **15** (10 mmol) and 2-mercaptoquinoline-3-carbaldehyde **11** (10 mmol, 1.89 g) were dissolved in 20 mL of DMF while heating until a homogeneous solution was achieved. The solution was then cooled, and 3 drops of trimethylchlorosilane were added. The reaction mixture was placed in a freezer for 5 h. The precipitated solid was filtered, washed with cold methanol, and air-dried. The obtained substance was subsequently used without further purification.

3-(((4*H*-1,2,4-triazol-4-yl)imino)methyl)quinoline-2-thiol (**3**).Yield 2.09 g (82%). Orange powder; mp 321–322 °C; IR (KBr) ν_max_ 3440.8, 2923.9, 1627.8 cm^−1^; ^1^H NMR (DMSO-d_6_, 400 MHz) δ 14.10 (1H, s, SH), 9.50 (1H, s, CH=N_azomethine_), 9.24 (2H, s, CH_triazole_), 8.58 (1H, s, CH), 8.01 (1H, dd, *J* = 8.0, 1.3 Hz, CH), 7.76 (1H, td, *J* = 7.5, 7.0, 1.3 Hz, CH), 7.69 (1H, dd, *J* = 8.4, 1.2 Hz, CH), 7.48–7.40 (1H, m, CH); ^13^C NMR (DMSO-d_6_, 101 MHz) δ 180.40, 156.18 (CH=N_azomethine_), 140.78, 139.62 (CH_triazole_), 135.32 (CH), 133.88 (CH), 131.37, 130.10 (CH), 125.48 (CH), 122.38, 116.71 (CH). HRMS (ESI), m/z: 278.0467 [M + Na]^+^ (calculated for C_12_H_9_N_5_SNa: 278.0471).

3-(((5-amino-1*H*-tetrazol-1-yl)imino)methyl)quinoline-2-thiol (**4**). Yield 2.2 g (81%). Orange powder; mp 261–262 °C; IR (KBr) ν_max_ 3132.2, 1624, 1581.5 cm^−1^; ^1^H NMR (DMSO-d_6_, 400 MHz) δ 14.11 (1H, s, SH), 9.74 (1H, s, CH=N), 8.80 (1H, s, CH), 7.87 (1H, d, *J* = 8.0 Hz, CH), 7.76 (1H, t, *J* = 7.8 Hz, CH), 7.69 (1H, d, *J* = 8.5 Hz, CH), 7.46 (1H, t, *J* = 7.4 Hz, CH), 7.36 (2H, s, NH_2_); ^13^C NMR (DMSO-d_6_, 101 MHz) δ 180.58, 153.34, 150.93, 140.71, 135.10, 133.90, 130.90, 129.72, 125.67, 122.39, 116.87; HRMS (ESI), m/z: 294.0522 [M + Na]^+^ (calculated for C_11_H_9_N_7_SNa: 294.0532).

**General procedure for the synthesis of compounds 5b–d**, **6b–d.**

To a flask equipped with a Dean–Stark trap, 20 mL of toluene, 2-mercaptoquinoline-3-carbaldehyde **11** (5 mmol, 0.95 g), tetrazole-5-amine **15b–d** or **16b–d** (5 mmol), and 3 drops of piperidine were added. The reaction mixture was refluxed under stirring for 4–6 h while simultaneously removing water into the trap. The reaction progress was monitored by TLC (petroleum ether (40–70 °C):ethyl acetate = 2:1). After cooling the reaction mixture to room temperature, it was placed in a freezer overnight. The precipitated solid was filtered, washed with petroleum ether (40–70 °C), and air-dried. The obtained substances were subsequently used without further purification.

3-(((1-ethyl-1*H*-tetrazol-5-yl)imino)methyl)quinoline-2-thiol (**5b**). Yield 1.07 g (75%). Orange powder; mp 278–279 °C; IR (KBr) ν_max_ 3465.1, 2923.9, 1608.5 cm^−1^; ^1^H NMR (DMSO-d_6_, 400 MHz) δ 14.12 (1H, s, SH), 10.06 (1H, s, CH=N), 8.88 (1H, s, CH), 8.06 (1H, dd, *J* = 8.2, 1.4 Hz, CH), 7.79 (1H, ddd, *J* = 8.5, 7.1, 1.4 Hz, CH), 7.69 (1H, d, *J* = 8.3 Hz, CH), 7.46 (1H, ddd, *J* = 8.1, 7.0, 1.1 Hz, CH), 4.53 (2H, q, *J* = 7.3 Hz, CH_2_), 1.51 (3H, t, *J* = 7.3 Hz, CH_3_); ^13^C NMR (DMSO-d_6_, 101 MHz) δ 181.48, 167.95, 158.34, 141.43, 137.36 (CH), 134.76 (CH), 132.25, 130.78 (CH), 125.64 (CH), 122.40, 116.85 (CH), 41.82 (CH_2_), 15.09 (CH_3_); HRMS (ESI), m/z: 307.0742 [M + Na]^+^ (calculated for C_13_H_12_N_6_SNa: 307.0736).

3-(((2-ethyl-2*H*-tetrazol-5-yl)imino)methyl)quinoline-2-thiol (**6b**). Yield 1.14 g (80%). Orange powder; mp 275–276 °C; IR (KBr) ν_max_ 3465.1, 2923.9, 1607.4 cm^−1^; ^1^H NMR (DMSO-d_6_, 400 MHz) δ 13.97 (1H, s, SH), 10.04 (1H, s, CH=N), 8.81 (1H, s, CH), 8.06 (1H, d, *J* = 8.1 Hz, CH), 7.81–7.72 (1H, m, CH), 7.69 (1H, d, *J* = 8.3 Hz, CH), 7.43 (1H, t, *J* = 7.7 Hz, CH), 4.72 (2H, q, *J* = 7.3 Hz, CH_2_), 1.57 (3H, t, *J* = 7.3 Hz, CH_3_); ^13^C NMR (DMSO-d_6_, 101 MHz) δ 181.42, 169.52, 165.60, 141.08, 136.19, 134.23, 132.54, 130.62, 125.46, 122.50, 116.70, 49.02 (CH_2_), 14.59 (CH_3_); HRMS (ESI), m/z: 307.0732 [M + Na]^+^ (calculated for C_13_H_12_N_6_SNa: 307.0736).

3-(((1-propyl-1*H*-tetrazol-5-yl)imino)methyl)quinoline-2-thiol (**5c**). Yield 1.7 g (72%). Orange powder; mp 255–256 °C; IR (KBr) ν_max_ 3503.2, 2927.7, 1624 cm^−1^; ^1^H NMR (DMSO-d_6_, 400 MHz) δ 14.09 (1H, s, SH), 10.05 (1H, s, CH=N), 8.83 (1H, s, CH), 8.04 (1H, dd, *J* = 8.1, 1.3 Hz, CH), 7.77 (1H, ddd, *J* = 8.3, 7.0, 1.5 Hz, CH), 7.67 (1H, d, *J* = 7.8 Hz, CH), 7.44 (1H, t, *J* = 7.3 Hz, CH), 4.46 (2H, t, *J* = 6.9 Hz, CH_2_), 1.92 (2H, h, *J* = 7.2 Hz, CH_2_), 0.90 (3H, t, *J* = 7.4 Hz, CH_3_); ^13^C NMR (DMSO-d_6_, 101 MHz) δ 181.46, 167.96, 158.67, 141.40, 137.28, 134.71, 132.23, 130.75, 125.58, 122.36, 116.82, 47.89 (CH_2_), 22.86 (CH_2_), 11.29 (CH_3_); HRMS (ESI), m/z: 321.0902 [M + Na]^+^ (calculated for C_14_H_14_N_6_SNa: 321.0893).

3-(((2-propyl-2*H*-tetrazol-5-yl)imino)methyl)quinoline-2-thiol (**6c**). Yield 1.12 g (75%). Orange powder; mp 225–226 °C; IR (KBr) ν_max_ 3505.7, 2927.7, 1608.5 cm^−1^; ^1^H NMR (DMSO-d_6_, 400 MHz) δ 14.05 (1H, s, SH), 10.04 (1H, s, CH=N), 8.81 (1H, s, CH), 8.06 (1H, d, *J* = 8.0 Hz, CH), 7.76 (1H, t, *J* = 7.7 Hz, CH), 7.69 (1H, d, *J* = 8.4 Hz, CH), 7.43 (1H, t, *J* = 7.5 Hz, CH), 4.66 (2H, t, *J* = 6.8 Hz, CH_2_), 1.98 (2H, h, *J* = 7.3 Hz, CH_2_), 0.91 (3H, t, *J* = 7.3 Hz, CH_3_); ^13^C NMR (DMSO-d_6_, 101 MHz) δ 181.44, 169.54, 165.64, 141.11, 136.25 (CH), 134.27 (CH), 132.52, 130.64 (CH), 125.49 (CH), 122.52, 116.71 (CH), 55.12 (CH_2_), 22.66 (CH_2_), 11.19 (CH_3_); HRMS (ESI), m/z: 321.0900 [M + Na]^+^ (calculated for C_14_H_14_N_6_SNa: 321.0893).

3-(((1-butyl-1*H*-tetrazol-5-yl)imino)methyl)quinoline-2-thiol (**5d**). Yield 1.22 g (78%). Orange powder; mp 241–242 °C; IR (KBr) ν_max_ 2915.6, 1627.5, 1587.2 cm^−1^; ^1^H NMR (DMSO-d_6_, 400 MHz) δ 14.10 (1H, s, SH), 10.07 (1H, s, CH=N), 8.85 (1H, s, CH), 8.06 (1H, dd, *J* = 8.0, 1.4 Hz, CH), 7.79 (1H, ddd, *J* = 8.5, 7.0, 1.5 Hz, CH), 7.69 (1H, d, *J* = 8.4 Hz, CH), 7.50–7.41 (1H, m, CH), 4.50 (2H, t, *J* = 6.9 Hz, CH_2_), 1.94–1.83 (2H, m, CH_2_), 1.31 (2H, h, *J* = 7.4 Hz, CH_2_), 0.92 (3H, t, *J* = 7.4 Hz. CH_3_); ^13^C NMR (DMSO-d_6_, 101 MHz) δ 181.48, 168.00, 158.64, 141.46, 137.30 (CH), 134.75 (CH), 132.25, 130.78 (CH), 125.61 (CH), 122.39, 116.88 (CH), 46.03 (CH_2_), 31.33 (CH_2_), 19.51 (CH_2_), 13.76 (CH_3_); HRMS (ESI), m/z: 335.1053 [M + Na]^+^ (calculated for C_15_H_16_N_6_SNa: 335.1049).

3-(((2-butyl-2*H*-tetrazol-5-yl)imino)methyl)quinoline-2-thiol (**6d**). Yield 1.19 g (76%). Orange powder; mp 203–204 °C; IR (KBr) ν_max_ 2931.6, 1627.8, 1581.5 cm^−1^; ^1^H NMR (DMSO-d_6_, 400 MHz) δ 14.06 (1H, s, SH), 10.04 (1H, s, CH=N), 8.82 (1H, s, CH), 8.07 (1H, dd, *J* = 8.1, 1.4 Hz, CH), 7.77 (1H, ddd, *J* = 8.4, 7.0, 1.4 Hz, CH), 7.69 (1H, d, *J* = 8.4 Hz, CH), 7.44 (1H, ddd, *J* = 8.1, 7.0, 1.2 Hz, CH), 4.70 (2H, t, *J* = 6.9 Hz, CH_2_), 2.00–1.88 (2H, m, CH_2_), 1.38–1.24 (2H, m, CH_2_), 0.92 (3H, t, *J* = 7.4 Hz, CH_3_); ^13^C NMR (DMSO-d_6_, 101 MHz) δ 181.45, 169.53, 165.64, 141.13, 136.26 (CH), 134.26 (CH), 132.53, 130.65 (CH), 125.48 (CH), 122.53, 116.73 (CH), 53.30 (CH_2_), 31.05 (CH_2_), 19.48 (CH_2_), 13.70 (CH_3_); HRMS (ESI), m/z: 335.1050 [M + Na]^+^ (calculated for C_15_H_16_N_6_SNa: 335.1049).

**Procedure for the synthesis of 4-hydroxy-7-methyl-2-phenyl-4*H*-thiopyrano[2**,**3-b]quinoline-3-carbaldehyde (2).**

In a flat-bottomed flask, 2-mercapto-6-methylquinoline-3-carbaldehyde **13** (5 mmol, 1.02 g) and triethylamine (15 mmol, 1.52 g) were dissolved in 10 mL of DMF. Then, freshly distilled 3-phenyl-2-propynal **12** (5 mmol, 0.65 g) was added. After 20 min, the reaction mixture was placed in a freezer overnight. The precipitated solid was filtered, washed with cold methanol, and air-dried. Further purification of the compound was not required. Yield 1.42 g (85%). Colorless powder; mp 219–220 °C; IR (KBr) ν_max_ 2198.2, 1655, 1569.6 cm^−1^; ^1^H NMR (DMSO-d_6_, 400 MHz) δ 9.35 (1H, s, CH=O), 8.51 (1H, d, *J* = 1.9 Hz, CH), 7.90 (1H, dd, *J* = 8.7, 1.8 Hz, CH), 7.83 (1H, d, *J* = 2.1 Hz, CH), 7.70–7.54 (6H, m, CH), 6.06–6.00 (1H, m, CH-OH), 5.77–5.71 (1H, m, OH), 2.52 (3H, m, CH_3_); ^13^C NMR (DMSO-d_6_, 101 MHz) δ 187.03, 158.81, 153.08, 145.75, 137.74 (CH), 136.87, 133.72, 133.62 (CH), 131.51 (CH), 130.71, 130.64, 129.51 (CH), 128.90, 127.88 (CH), 127.82, 127.30 (CH), 62.53 (CH-OH), 21.62 (CH_3_); HRMS (ESI), m/z: 356.0721 [M + Na]^+^ (calculated for C_20_H_15_NO_2_SNa: 356.0716).

**General procedure for the synthesis of compounds 7**, **8**, **9b–d**, **10b–d.**

In a flat-bottomed flask, azomethine **3**, **4**, **5b–d** or **6b–d** (5 mmol) and triethylamine (15 mmol, 1.52 g) were dissolved in 10 mL of DMF. Then, freshly distilled 3-phenyl-2-propynal **12** (5 mmol, 0.65 g) was added. After 20 min, the reaction mixture was placed in a freezer overnight. If no precipitate was observed, a few drops of water were added to the reaction mixture and left in the freezer for a few more hours. The precipitated solid was filtered, washed with cold methanol, and air-dried. Further purification of the compounds was not required.

4-((4*H*-1,2,4-triazol-4-yl)amino)-2-phenyl-4*H*-thiopyrano[2,3-b]quinoline-3-carbaldehyde (**7**). Yield 1.58 g (82%). Colorless powder; mp 247–248 °C; IR (KBr) ν_max_ 3274.9, 1643.2, 1546.8 cm^−1^; ^1^H NMR (DMSO-d_6_, 400 MHz) δ 9.26 (1H, s, CH=O), 8.53 (1H, s, CH), 8.26 (2H, s, CH_triazole_), 8.03 (2H, dd, *J* = 8.2, 1.3 Hz, CH), 7.88 (1H, ddd, *J* = 8.4, 6.9, 1.4 Hz, CH), 7.72–7.57 (6H, m, CH), 7.36 (1H, d, *J* = 3.0 Hz, NH), 5.94 (1H, d, *J* = 2.9 Hz, CH)_;_^13^C NMR (DMSO-d_6_, 101 MHz) δ 186.48, 161.29, 154.36, 147.26, 144.05 (CH), 139.36 (CH), 133.29, 131.89, 131.84, 130.81 (CH), 129.60 (CH), 128.86, 128.22, 127.57 (CH), 127.51, 126.45, 124.84, 56.18 (CH-NH); HRMS (ESI), m/z: 408.0892 [M + Na]^+^ (calculated for C_21_H_15_N_5_OSNa: 408.0890).

4-((5-amino-1*H*-tetrazol-1-yl)amino)-2-phenyl-4*H*-thiopyrano[2,3-b]quinoline-3-carbaldehyde (**8**). Yield 1.67 g (84%). Colorless powder; mp 228–229 °C; IR (KBr) ν_max_ 3174.6, 1658.7, 1581.5 cm^−1^; ^1^H NMR (DMSO-d_6_, 400 MHz) δ 9.26 (1H, s, CH=O), 8.46 (1H, s, CH), 8.01 (2H, dt, *J* = 8.2, 1.4 Hz, CH), 7.85 (1H, ddd, *J* = 8.5, 6.8, 1.4 Hz, CH), 7.74–7.68 (2H, m, CH), 7.68–7.56 (5H, m, CH), 6.22 (2H, s, NH_2_), 5.98 (1H, d, *J* = 3.6 Hz, CH-NH_2_); ^13^C NMR (DMSO-d_6_, 101 MHz) δ 186.52, 160.82, 154.90, 154.45, 147.20, 139.53 (CH), 133.52, 131.85 (CH), 131.72, 130.80 (CH), 129.54 (CH), 128.88, 128.13, 127.53 (CH), 127.41, 126.35, 124.73, 55.08 (CH-NH); HRMS (ESI), m/z: 424.0938 [M + Na]^+^ (calculated for C_20_H_15_N_7_OSNa: 424.0951).

4-((1-ethyl-1*H*-tetrazol-5-yl)amino)-2-phenyl-4*H*-thiopyrano[2,3-b]quinoline-3-carbaldehyde (**9b**). Yield 1,62 g (78%). Colorless powder; mp 245–246 °C; IR (KBr) ν_max_ 3220.9, 1666.4, 1577.7 cm^−1^; ^1^H NMR (DMSO-d_6_, 400 MHz) δ 9.35 (1H, s, CH=O), 8.87 (1H, s, CH), 8.10 (1H, d, J = 8.2 Hz, CH), 7.99 (1H, d, J = 8.5 Hz, CH), 7.84 (1H, td, *J* = 7.2, 3.6 Hz, CH), 7.77–7.70 (2H, m, CH), 7.70–7.58 (4H, m, CH), 7.45 (1H, d, *J* = 6.5 Hz, NH), 6.46 (1H, d, *J* = 6.2 Hz, CH-NH), 4.09 (2H, q, *J* = 7.2 Hz, CH_2_), 1.20 (3H, t, *J* = 7.2 Hz, CH_3_); ^13^C NMR (DMSO-d_6_, 101 MHz) δ 186.59, 162.81, 159.67, 154.52, 153.87, 147.01, 138.49 (CH), 133.43, 131.75 (CH), 131.61 (CH), 130.91 (CH), 129.49 (CH), 129.02 (CH), 127.99 (CH), 127.74, 127.48, 127.43 (CH), 126.68, 49.33 (CH-NH), 40.49 (CH_2_), 14.44 (CH_3_); HRMS (ESI), m/z: 437.1157 [M + Na]^+^ (calculated for C_22_H_18_N_6_OSNa: 437.1155).

4-((2-ethyl-2*H*-tetrazol-5-yl)amino)-2-phenyl-4*H*-thiopyrano[2,3-b]quinoline-3-carbaldehyde (**10b**). Yield 1.84 g (89%). Colorless powder; mp 231–232 °C; IR (KBr) ν_max_ 3313.5, 1643.2, 1550.7cm^−1^; ^1^H NMR (DMSO-d_6_, 400 MHz) δ 9.32 (1H, s, CH=O), 8.78 (1H, s, CH), 8.07 (1H, d, *J* = 8.1 Hz, CH), 7.97 (1H, d, *J* = 8.5 Hz, CH), 7.85–7.72 (3H, m, CH), 7.66–7.57 (4H, m, CH), 7.52 (1H, d, *J* = 5.8 Hz, NH), 6.22 (1H, d, *J* = 5.1 Hz, CH), 4.47 (2H, qd, *J* = 7.1, 3.7 Hz), 1.45 (3H, t, *J* = 7.3 Hz); ^13^C NMR (DMSO-d_6_, 101 MHz) δ 186.47, 166.58, 166.53, 159.76, 154.23, 146.83, 138.28 (CH), 133.59, 131.49, 131.37, 130.91 (CH), 129.35 (CH), 128.86, 128.25, 127.97 (CH), 127.35, 126.86, 48.58 (CH-NH), 48.00 (CH_2_), 14.53 (CH_3_); HRMS (ESI), m/z: 437.1151 [M + Na]^+^ (calculated for C_22_H_18_N_6_OSNa: 437.1155).

2-phenyl-4-((1-propyl-1*H*-tetrazol-5-yl)amino)-4*H*-thiopyrano[2,3-b]quinoline-3-carbaldehyde (**9c**). Yield 1.82 g (85%). Colorless powder; mp 247–248 °C; IR (KBr) ν_max_ 3218.5, 1668.2, 1565.7 cm^−1^; ^1^H NMR (DMSO-d_6_, 400 MHz) δ 9.35 (1H, s, CH=O), 8.86 (1H, s, CH), 8.10 (1H, d, *J* = 8.2 Hz, CH), 7.98 (1H, d, *J* = 8.5 Hz, CH), 7.83 (1H, t, *J* = 7.7 Hz, CH), 7.72 (2H, dd, *J* = 7.2, 2.0 Hz, CH), 7.63 (4H, q, *J* = 6.9, 6.2 Hz, CH), 7.44 (1H, d, *J* = 6.5 Hz, NH), 6.46 (1H, d, *J* = 6.5 Hz, CH-NH), 4.03 (2H, td, *J* = 6.9, 4.1 Hz, CH_2_), 1.61 (2H, h, *J* = 7.2 Hz, CH_2_), 0.69 (3H, t, *J* = 7.4 Hz, CH_3_); ^13^C NMR (DMSO-d_6_, 101 MHz) δ 186.58, 159.56, 154.87, 153.82, 147.01, 138.41 (CH), 133.43, 131.74, 131.59 (CH), 130.90 (CH), 129.51 (CH), 129.01, 127.98 (CH), 127.73, 127.49, 127.42 (CH), 126.68, 49.36 (CH-NH), 46.53 (CH_2_), 22.23 (CH_2_), 10.95 (CH_3_); HRMS (ESI), m/z: 451.1317 [M + Na]^+^ (calculated for C_23_H_20_N_6_OSNa: 451.1312).

2-phenyl-4-((2-propyl-2*H*-tetrazol-5-yl)amino)-4*H*-thiopyrano[2,3-b]quinoline-3-carbaldehyde (**10c**). Yield 1.93 g (90%). Colorless powder; mp 237–238 °C; IR (KBr) ν_max_ 3305.8, 1666.4, 1566.1 cm^−1^; ^1^H NMR (DMSO-d_6_, 400 MHz) δ 9.32 (1H, s, CH=O), 8.77 (1H, s, CH), 8.09–8.03 (1H, m, CH), 7.98 (1H, dd, *J* = 8.6, 5.0 Hz, CH), 7.82 (1H, ddd, *J* = 8.5, 6.8, 1.4 Hz, CH), 7.76 (2H, dd, *J* = 7.6, 1.9 Hz, CH), 7.67–7.56 (4H, m, CH), 7.53 (1H, d, *J* = 5.9 Hz, NH), 6.22 (1H, d, *J* = 5.9 Hz, CH-NH), 4.47–4.35 (2H, m, CH_2_), 1.88 (2H, hd, *J* = 7.0, 2.5 Hz, CH_2_), 0.77 (3H, t, *J* = 7.4 Hz, CH_3_); ^13^C NMR (DMSO-d_6_, 101 MHz) δ 186.49, 166.56, 154.20, 146.82, 138.21 (CH), 133.57, 131.52, 131.39 (CH), 130.92 (CH), 129.36 (CH), 128.84 (CH), 128.21, 127.96 (CH), 127.32 (CH), 126.82, 54.19 (CH_2_), 48.60 (CH-NH), 22.46 (CH_2_), 11.19 (CH_3_); HRMS (ESI), m/z: 451.1308 [M + Na]^+^ (calculated for C_23_H_20_N_6_OSNa: 451.1312).

4-((1-butyl-1*H*-tetrazol-5-yl)amino)-2-phenyl-4*H*-thiopyrano[2,3-b]quinoline-3-carbaldehyde (**9d**). Yield 1.84 g (83%). Colorless powder; mp 220–221 °C; IR (KBr) ν_max_ 3232.5, 1662.5, 1569.9 cm^−1^; ^1^H NMR (DMSO-d_6_, 400 MHz) δ 9.35 (1H, s, CH=O), 8.84 (1H, s, CH), 8.09 (1H, d, *J* = 8.2 Hz, CH), 7.99 (1H, d, *J* = 8.5 Hz, CH), 7.83 (1H, ddd, *J* = 8.4, 6.8, 1.4 Hz, CH), 7.71 (2H, dd, *J* = 7.1, 2.2 Hz, CH), 7.68–7.58 (4H, m, CH), 7.43 (1H, d, *J* = 6.5 Hz, NH), 6.46 (1H, d, *J* = 6.4 Hz, CH-NH), 4.06 (2H, dt, *J* = 11.8, 7.1 Hz, CH_2_), 1.55 (2H, p, *J* = 7.2 Hz, CH_2_), 1.03 (2H, dtd, *J* = 13.2, 8.4, 7.9, 4.2 Hz, CH_2_), 0.71 (3H, t, *J* = 7.3 Hz, CH_3_); ^13^C NMR (DMSO-d_6_, 101 MHz) δ 186.57, 159.59, 154.83, 153.82, 147.01, 138.36 (CH), 133.43, 131.75, 131.60 (CH), 130.87 (CH), 129.51 (CH), 129.01, 127.97 (CH), 127.74, 127.47 (CH), 127.42, 126.64, 49.36 (CH-NH), 44.80 (CH_2_), 30.70 (CH_2_), 19.21 (CH_2_), 13.69 (CH_3_); HRMS (ESI), m/z: 465.1476 [M + Na]^+^ (calculated for C_24_H_22_N_6_OSNa: 465.1468).

4-((2-butyl-2*H*-tetrazol-5-yl)amino)-2-phenyl-4*H*-thiopyrano[2,3-b]quinoline-3-carbaldehyde (**10d**). Yield 1.9 g (86%). Colorless powder; mp 204–205 °C; IR (KBr) ν_max_ 3240.2, 1666.4, 1558.4 cm^−1^; ^1^H NMR (DMSO-d_6_, 400 MHz) δ 9.32 (1H, s, CH=O), 8.76 (1H, s, CH), 8.09–8.02 (1H, m, CH), 7.97 (1H, d, *J* = 8.6 Hz, CH), 7.81 (1H, ddd, *J* = 8.4, 6.8, 1.4 Hz, CH), 7.78–7.72 (2H, m, CH), 7.67–7.56 (4H, m, CH), 7.54 (1H, d, *J* = 5.9 Hz, NH), 6.20 (1H, d, *J* = 5.8 Hz, CH-NH), 4.52–4.37 (2H, m, CH_2_), 1.81 (2H, ddt, *J* = 13.6, 6.7, 4.2 Hz, CH_2_), 1.15 (2H, h, *J* = 7.5 Hz, CH_2_), 0.80 (3H, t, *J* = 7.3 Hz, CH_3_); ^13^C NMR (DMSO-d_6_, 101 MHz) δ 186.48, 166.54, 159.80, 154.23, 146.82, 138.26 (CH), 133.58, 131.51 (CH), 131.38, 130.93, 129.35 (CH), 128.84, 128.21, 127.98 (CH), 127.30 (CH), 126.78, 52.36 (CH_2_), 48.61 (CH-NH), 30.96 (CH_2_), 19.48 (CH_2_), 13.66 (CH_3_); HRMS (ESI), m/z: 465.1473 [M + Na]^+^ (calculated for C_24_H_22_N_6_OSNa: 465.1468).

### 3.3. X-ray Diffraction

The structure of the target 4-hydroxy-4*H*-thiopyrano[2,3-b]quinolines was determined using the example of compound **1**. The structure of the target tetrazole-containing 4*H*-thiopyrano[2,3-b]quinolines was determined using the example of compounds **9a** and **10a**, which have different positions of the substituent in the tetrazole ring. Suitable crystals were studied using an Agilent Technologies “SuperNova” (**9a**) and Rigaku «XtaLAB Synergy-S» (**1**, **10a**) diffractometers (mono-chromated Cu Kα radiation, λ= 1.54184 Å). The temperature was kept at 100(2) K throughout the experiment. Empirical absorption correction was applied in the CrysAlisPro (Agilent Technologies, 2014; Rigaku OD, 2021) program complex using spherical harmonics, implemented in the SCALE3 ABSPACK scaling algorithm. The structures were solved by the SHELXT [36] program, using least squares minimization in anisotropic (for non-hydrogen atoms) approximation and refining with the SHELXL package [37] incorporated in the Olex2 program package [38]. The hydrogen atoms were introduced to the geometrically calculated positions and refined by attaching themselves to the corresponding parent atoms. The disorder within one independent half of molecule **10a** was modeled by the free-populated superposition of two sets of the benzene ring coordinates corresponding to slightly different positions of the ring in space. Crystallographic data for the studied samples have been deposited at Cambridge Crystallographic Data center (**1**: CCDC 2289287; **9a**: CCDC 2271109; **10a**: CCDC–2271110).

### 3.4. Biological Activity

Influenza virus A/Puerto Rico/8/34 (H1N1) was obtained from the collection of viruses of the St. Petersburg Pasteur Institute. Before the experiment, the virus was propagated in the allantoic cavity of 10- to 12-day-old chicken embryos for 48 h at 36 °C. The infectious titer of the virus was determined in Madin-Darby Canine Kidney (MDCK) cells (ATCC-CCL-34) grown in 96-well plates in alpha-MEM medium with 10% fetal bovine serum.

#### 3.4.1. Cytotoxicity Assay

MDCK cells were seeded onto 96-well culture plates (104 cells per well) and incubated at 36 °C in 5% CO_2_ until a continuous monolayer formation. To assess the toxicity of compounds, a series of their 3-fold dilutions at concentrations of 300 to 3.7 μg/mL in Eagle’s Minimal Essential Medium (MEM) were prepared. The dilutions were added to the wells of the plates. Cells were incubated for 72 h at 36 °C in a CO_2_ incubator under 5% CO_2_. Further, a microtetrazolium (MTT) assay was performed on 96-well plates. The cells were washed 2 times with saline (0.9% NaCl), and 100 μL/well of MTT solution [3-(4,5-dimethylthiazol-2-yl)-2,5-diphenyltetrazolium bromide] at a concentration of 0.5 g/mL in MEM was added. The plates were incubated for 1 h at 36 °C, the liquid was removed, and dimethylsulfoxide (DMSO) (0.1 mL per well) was added. The optical density (OD) of the cells was measured on a Thermo Multiskan FC spectrophotometer (Thermo Fisher Scientific, Waltham, MA, USA) at a wavelength of 540 nm. Based on the obtained data, the CC50, the concentration of the compound that destroys 50% of the cells in the culture, was calculated for each specimen.

#### 3.4.2. CPE Reduction Assay

The compounds, in appropriate concentrations, were added to MDCK cells (0.1 mL per well). MDCK cells were further infected with A/Puerto Rico/8/34 (H1N1) influenza virus (m.o.i 0.01). Plates were incubated for 72 h at 36 °C at 5% CO_2_. After that, cell viability was assessed by the MTT test, as described above. The cytoprotective activity of compounds was considered as their ability to increase the values of the OD compared to the control wells (with virus only; no drugs). Based on the obtained results, the IC_50_ values, i.e., the concentration of compounds that results in 50% cell protection, were calculated using GraphPad Prism 6.01 software. IC_50_ values in μg/mL were then calculated into micromoles. For each compound, the value of the selectivity index (SI) was calculated as a ratio of CC_50_ to IC_50_.

### 3.5. Molecular Modeling

#### 3.5.1. Protein Structure Preparation

We have observed six main targets, popular in drug development against influenza virus A/H1N1/Puerto Rico/8/34: M2 channel, neuraminidase N1 (3TI6) of influenza virus, HA (hemagglutinin, 1RU7), PB2 (polymerase basic protein 2, 4U6O), and PA (polymerase acidic protein, 5FDG). NP (nucleoprotein, 5TJW) is from Influenza A virus (A/WSN/1933(H1N1)), but its homology to the NP of A/H1N1/PR is 99%.

All structures were downloaded from RCSB Protein Data bank. With the exception of M2 channel, their structure is unavailable in Protein Data Bank (for A/H1N1/Puerto Rico/8/34 strain). All protein models need to be preprocessed with use of Schrodinger Protein PrepWizard [39]. In this step, we exclude typical structure errors, such as: invalid protonation state, missing aminoacids sidechains, missing loops, and incorrect bond orders. All water molecules are deleted from protein models. In the metals, zero-order bonds were built. All metal ions crucial for protein native function were conserved.

In the case of M2 channel, the protein structure for the A/Puerto Rico/8/1934 H1N1 strain was not available in RCSB PDB. However, the protein sequence can be found in the Uniprot database: matrix protein 2 influenza A virus (strain A/Puerto Rico/8/1934 H1N1), accession code P06821. M2 protein structure was built with use of M2 channel model 2RLF [40] (H3N2) and with point mutations and geometry optimization transformed into H1N1. The resulting mutated model geometry was optimized with use of the Schrodinger Prime module.

For all observed protein models, protonation states, hydrogen bonds, and charges were calculated for pH = 7.4 with use of the PROPKA method [41]. Protein structure was refined with the use of restrained minimization (needed for elimination of local strained contacts in protein).

All manipulations with proteins and ligands were carried out in OPLS4 [42] forcefield.

#### 3.5.2. Ligand Structure Preparation

Ligand structures were prepared with the use of the LigPrep module included in the Schrodinger suite. Ionization states were predicted with the use of the Epik module. The three-dimensional structure of ligands, used for calculations, was generated in the same forcefield—OPLS4 [42]. Stereoisomers were generated on the basis of predefined chiral centers.

#### 3.5.3. Molecular Docking: GridBox Building

Before molecular docking, a grid box was built, covering the ligand-binding pocket. Gridbox size was chosen in accordance with reference ligand size, which is present in the protein model, and equals 10 Å (one side of the cube). Grid center was placed in the ligand centroid. In the M2 channel cavity, there was placement of the gridbox on selection centroid: Gly34 in chains A-D, cube side size is 10 Å. VdW scaling is 1.0 Å, partial charge cutoff—0.25 Å. No constraints were applied for all observed proteins.

The GlideGrid [43] program was used for calculations.

#### 3.5.4. Molecular Docking: Docking Procedure

Prepared ligands were docked into previously generated GridBoxes of our proteins: neuraminidase, M2 channel, hemagglutinin, nucleoprotein, PB2, and PA. For all the ligands, the standard precision mode with enhanced flexible sampling was selected. State penalty value was calculated for each ligand (if the ligand is ionizable), 15 docking solutions were generated for each structure, and strain correction terms were added (necessary for ligand torsions parametrization before final scoring). Resulting docking solutions were clustered, and the best-fitting solution was compared to the reference structure binding mode and by Glidescore/Emodel values and its components.

## 4. Conclusions

In the article, we have presented a novel and efficient method of the synthesis of 1,2,4-triazole- and tetrazole-containing 4*H*-thiopyrano[2,3-b]quinolines based on the thio-Michael/aza-Morita–Baylis–Hillman tandem reaction. This method enables the synthesis of complex molecules using simple building blocks such as 2-mercaptoquinoline-3-carbaldehyde, 4-amino-4*H*-1,2,4-triazole-3-thiol, 1-amino-1*H*-tetrazole-5-thiol, and 3-phenyl-2-propynal, thus expanding the possibilities for the generation of diverse derivatives of 4*H*-thiopyrano[2,3-b]quinolines. Moreover, it is worth noting the high potential of the obtained thiopyrano[2,3-b]quinolines, which contain a highly reactive carbonyl group and an activated double bond, for further chemical transformations.

The cytotoxic and antiviral properties of the synthesized compounds **1**, **2**, **7**, **8**, **9a–d**, and **10a–d** were tested in vitro against influenza A/Puerto Rico/8/34 (H1N1) virus in MDCK cells. The studies revealed low toxicity of these compounds, with only 2 (**10a** and **10d**) out of the 12 tested compounds showing cytotoxicity. Compounds **1**, **9a**, and **10c** exhibited the highest activity (SI = 31, >16, >38, respectively), surpassing rimantadine (SI = 5) significantly. The calculations conducted for the investigated compounds have shown that the most preferable targets are the M2 channel and polymerase basic protein 2. The interaction with the first target is energetically favorable due to the significant contribution of lipophilic interactions, which ensures binding specificity. On the other hand, for PB2, the compounds bind primarily through electrostatic interactions provided by the aromatic structure of the scaffold, as well as the electron density of the tetrazole/triazole fragments.

Through in vitro investigations and the interpretation of computer data, we have discerned that both the nature and position of the substituent within the tetrazole ring, along with the substituent in the benzene moiety of quinoline, have a substantial impact on antiviral effectiveness of studied thiopyrano[2,3-b]quinolines. Guided by this revelation, our intention is to persist in further pursuit of active compounds by modifying the nature of the mentioned substituents.

## Figures and Tables

**Table 1:** Cytotoxic and anti-viral properties of synthesized compounds **1**, **2**, **7**, **8**, **9a–d**, and **10a–d** according to in vitro tests against influenza virus A/Puerto Rico/8/34 in MDCK cells.

Compound	N	^a^ CC_50_, µM	^b^ IC_50_, µM	^c^ SI	Compound	N	^a^ CC_50_, µM	^b^ IC_50_, µM	^c^ SI
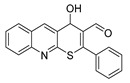	**1**	>900	30 ± 5	31	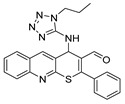	**9c**	>700	293.8 ± 30.8	>2
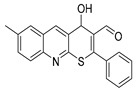	**2**	>900	293.9 ± 31.3	>3	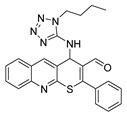	**9d**	>650	384.1 ± 42.1	>2
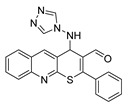	**7**	>750	161.4 ± 10.9	>5	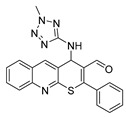	**10a**	549 ± 26.6	>249.7	<2
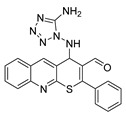	**8**	>750	190.3 ± 26.4	>4	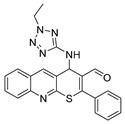	**10b**	>700	605.6 ± 62.9	>1
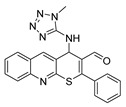	**9a**	>750	46 ± 5	>16	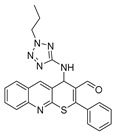	**10c**	>700	18.4 ± 2.7	>38
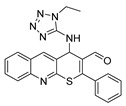	**9b**	>700	>700	1	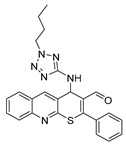	**10d**	68.9 ± 42.4	>24.9	<3
**Rimantadine**		312.3 ± 22.8	64.1 ± 7.2	5	**Oseltamivir** **carboxylate**		>100	0.17 ± 0.02	>588

Abbreviations: ^a^ CC_50_, cytotoxic concentration—the concentration resulting in 50% death of cells; ^b^ IC_50_, effective concentration—the concentration resulting in 50% inhibition of virus replication; ^c^ SI, selectivity index—ratio CC_50_/IC_50_.

**Table 2:** Binding glidescore values for the ligand–protein complexes of the investigated compounds with six targets of the influenza A/H1N1/Puerto Rico/8/34 virus. Positive control compounds for each target: oseltamivir (NA), rimantadine (M2), camphecene (HA), native ligand from model 3TG6 (NP), native ligand VX787 from 7AS2 model (PB2), and inhibitor from 5FDG model (PA).

Compound	GlideScore@Target (kcal/mol)						Activity (IC_50_)
	NA	M2	HA	NP	PB2	PA	
**1**	−4.61	−7.90	-	−4.26	−5.33	−4.87	30 ± 5
**2**	−4.25	−5.90	-	−4.21	−5.30	−5.10	293.9 ± 31.3
**7**	−3.99	−7.79	-	−3.71	−6.41	−4.71	161.4 ± 10.9
**8**	−4.45	−7.95	-	−3.65	−6.23	−4.84	190.3 ± 26.4
**9a**	−4.34	−8.34	-	−3.16	−6.37	−4.62	46 ± 5
**9b**	−3.74	−4.05	-	−3.80	−4.42	−4.73	>700
**9c**	−4.06	−3.88	-	−2.75	−5.11	−5.15	293.8 ± 30.8
**9d**	−3.87	−3.51	-	−2.32	-	−5.15	384.1 ± 42.1
**10a**	−3.95	−3.99	-	−3.28	−4.26	−5.07	>249.7
**10b**	−3.62	−5.98	-	−2.95	−5.27	−4.30	605.6 ± 62.9
**10c**	−3.40	−5.08	-	−3.92	−5.33	−4.57	18.4 ± 2.7
**10d**	−3.26	−4.90	-	−3.37	−5.00	−4.57	>24.9
Control	−5.50	−6.60	−7.10	−6.56	−6.05	−6.56	-

Cell color differentiation: orange—structures with low predicted activity; yellow—affinity to the active site below the reference by up to 1.5 kcal/mol; green—at or better than the reference level.

**Table 3:** Binding glidescore values and MM-GBSA free energy components calculated for best-fitting binding poses of observed ligands in comparison with IC_50_ values.

Compound	Glidescore, (kcal/mol)		MMGBSA ΔG, (kcal/mol)		IC_50_
	Neuraminidase	M2	M2—Lipo	M2—Strain Energy	
**1**	−4.61	−7.90	−14.07	0.96	30 ± 5
**2**	−4.25	−5.90	−15.04	2.08	293.9 ± 31.3
**9a**	−4.34	−8.34	−11.42	2.02	46 ± 5
**10a**	−3.74	−3.99	−12.81	2.58	>249.7
**Rimantadine**	-	−6.60	−13.25	0.78	64.1 ± 7.2
**Oseltamivir**	−5.50	-	-	-	0.17 ± 0.02

## Data Availability

Data are contained within the article and Supplementary Materials.

## Supplementary Materials

The following supporting information can be downloaded at: https://www.mdpi.com/article/10.3390/molecules28217427/s1, Figures S1–S17—^1^H NMR spectra of the compounds **2**, **3**, **4**, **5b–d**, **6b–d**, **7**, **8**, **9b–d**, and **10b–d**; Figures S18–S34—^13^C NMR spectra of the compounds **2**, **3**, **4**, **5b–d**, **6b–d**, **7**, **8**, **9b–d**, and **10b–d**; Figures S35–S51—mass spectra of the compounds **2**, **3**, **4**, **5b–d**, **6b–d**, **7**, **8**, **9b–d**, and **10b–d**; Figure S52—Binding poses of compounds **7**, **8**, and **9a** and native control from 4U6O pdb model in PB2 active pocket; Figure S53—an example of ^1^H NMR spectrum processing for the compound **6c** (**a**) before processing, and (**b**) after processing; Figure S54—an example of ^13^C NMR spectrum processing for the compound **6c** (**a**) before processing, and (**b**) after processing.
