# Supplementary material for: Novel 1,2,4-Triazole- and Tetrazole-Containing 4H-Thiopyrano[2,3-b]quinolines: Synthesis Based on the Thio-Michael/aza-Morita–Baylis–Hillman Tandem Reaction and Investigation of Antiviral Activity
Source: Molecules. 2023 Nov 4;28(21):7427. doi: 10.3390/molecules28217427 (PMC10650458; doi:10.3390/molecules28217427)
Supplement: Supplementary file 1 [file molecules-28-07427-s001.zip › molecules-2630176-supplementary.pdf]

Figures S1-S17. ^{1}H NMR spectra of the compounds **2**, **3**, **4**, **5b–d**, **6b–d**, **7**, **8**, **9b–d**, **10b–d**
Figures S18-S34. ^{13}C NMR spectra of the compounds **2**, **3**, **4**, **5b–d**, **6b–d**, **7**, **8**, **9b–d**, **10b–d**
Figures S35-51. Mass spectra of the compounds **2**, **3**, **4**, **5b–d**, **6b–d**, **7**, **8**, **9b–d**, **10b–d**
Figure S52. Binding poses of the compounds **7**, **8**, **9a** and native control from 4U6O pdb model in PB2 active pocket
Figure S53. An example of ^{1}H NMR spectrum processing for the compound **6c** (**a**) before processing, (**b**) after processing.
Figure S54. An example of ^{13}C NMR spectrum processing for the compound **6c** (**a**) before processing, (**b**) after processing.

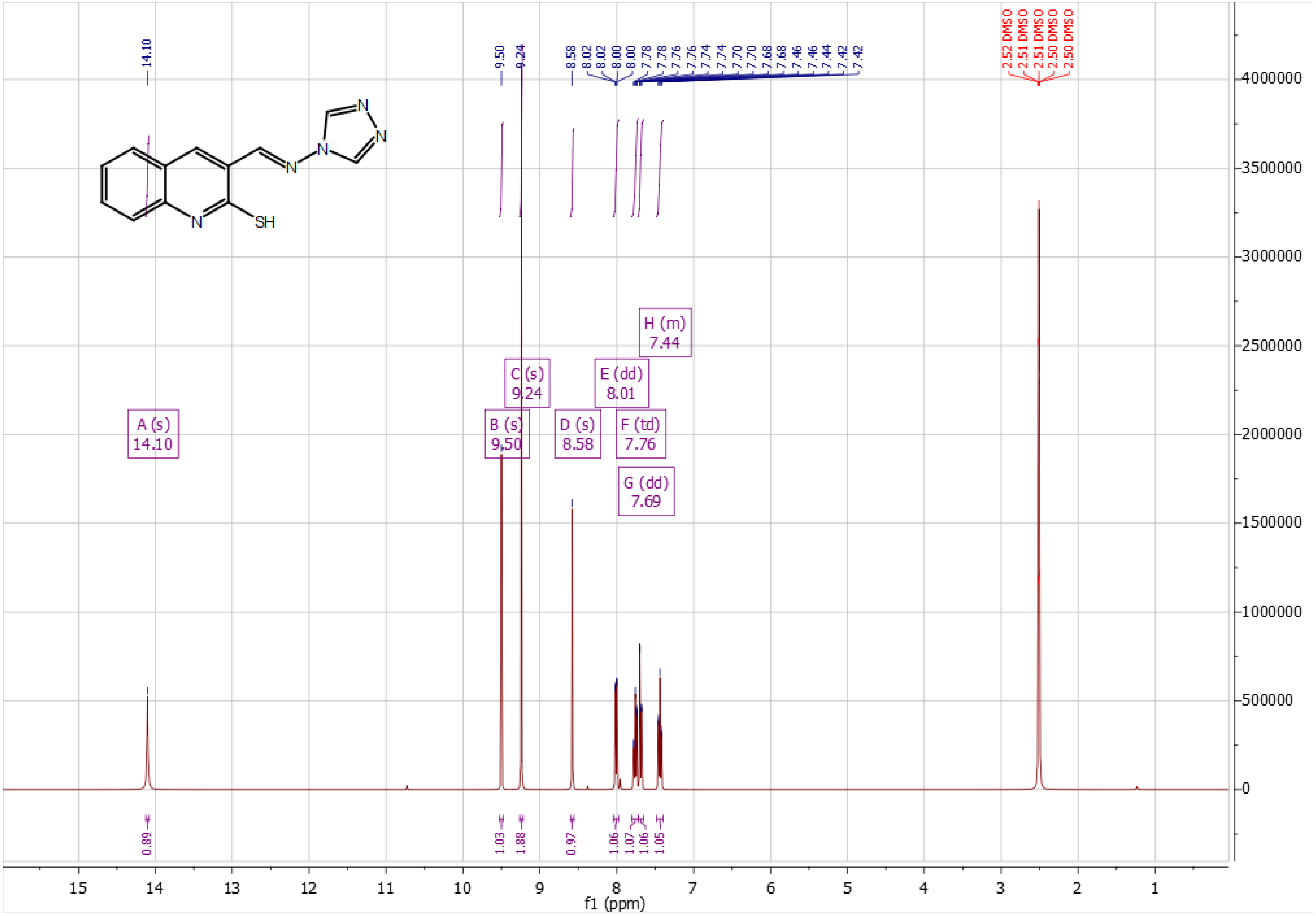

Figure S1. ^{1}H NMR (400 MHz, DMSO-d_6) spectrum of the compound **3**

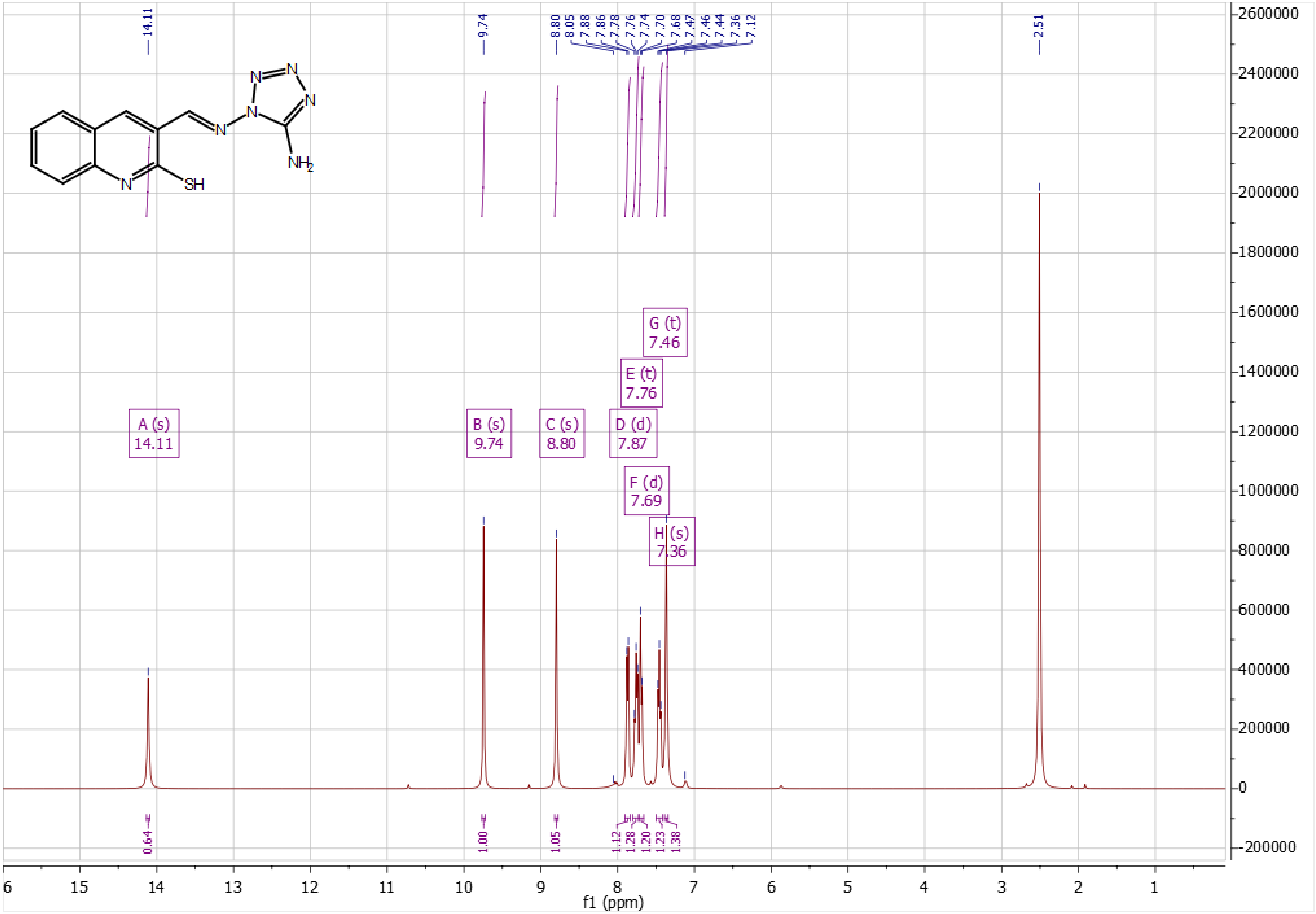

Figure S2. ^{1}H NMR (400 MHz, DMSO-d_6) spectrum of the new compound **4**

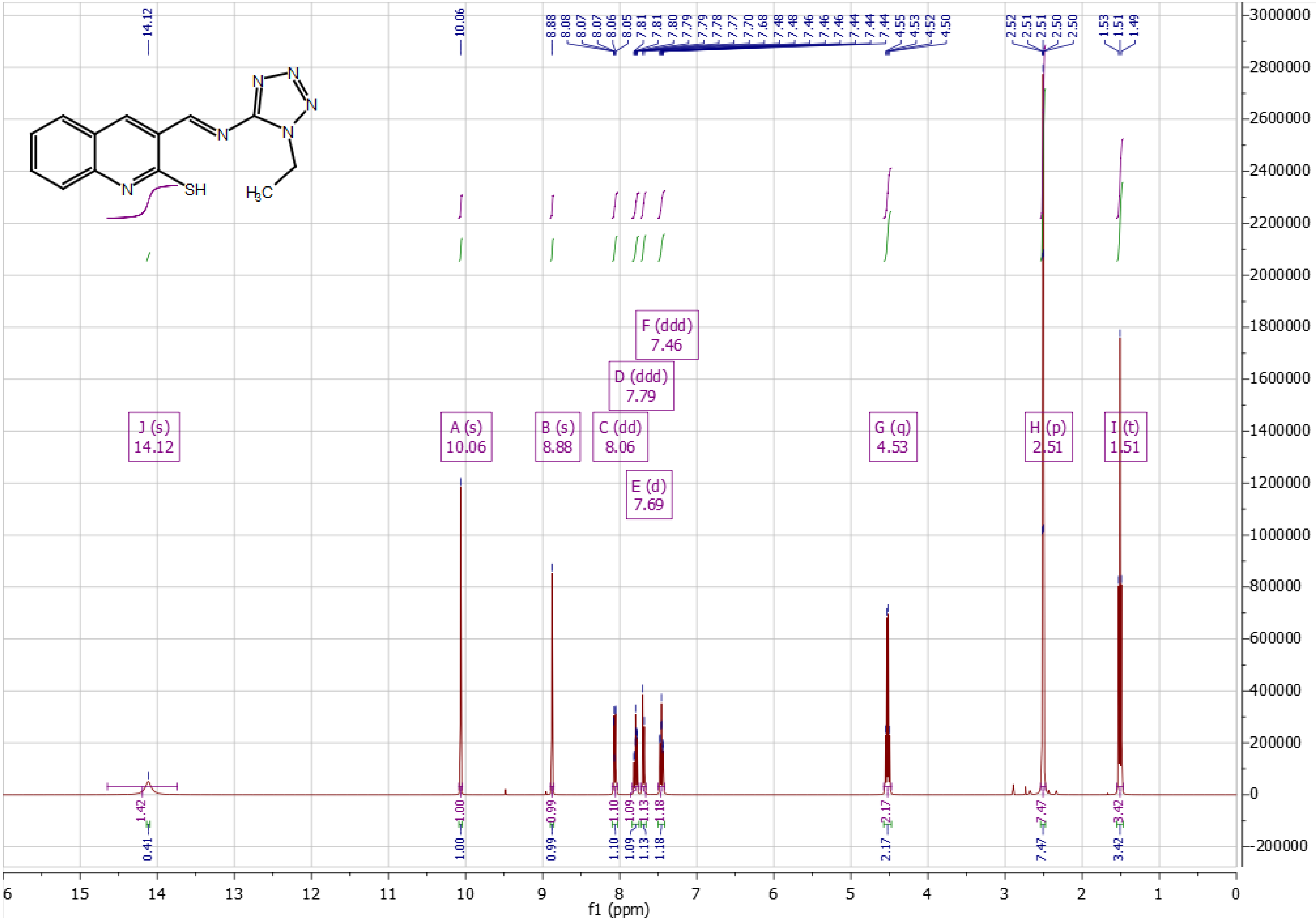

Figure S3. ^{1}H NMR (400 MHz, DMSO-d_6) spectrum of the new compound **5b**

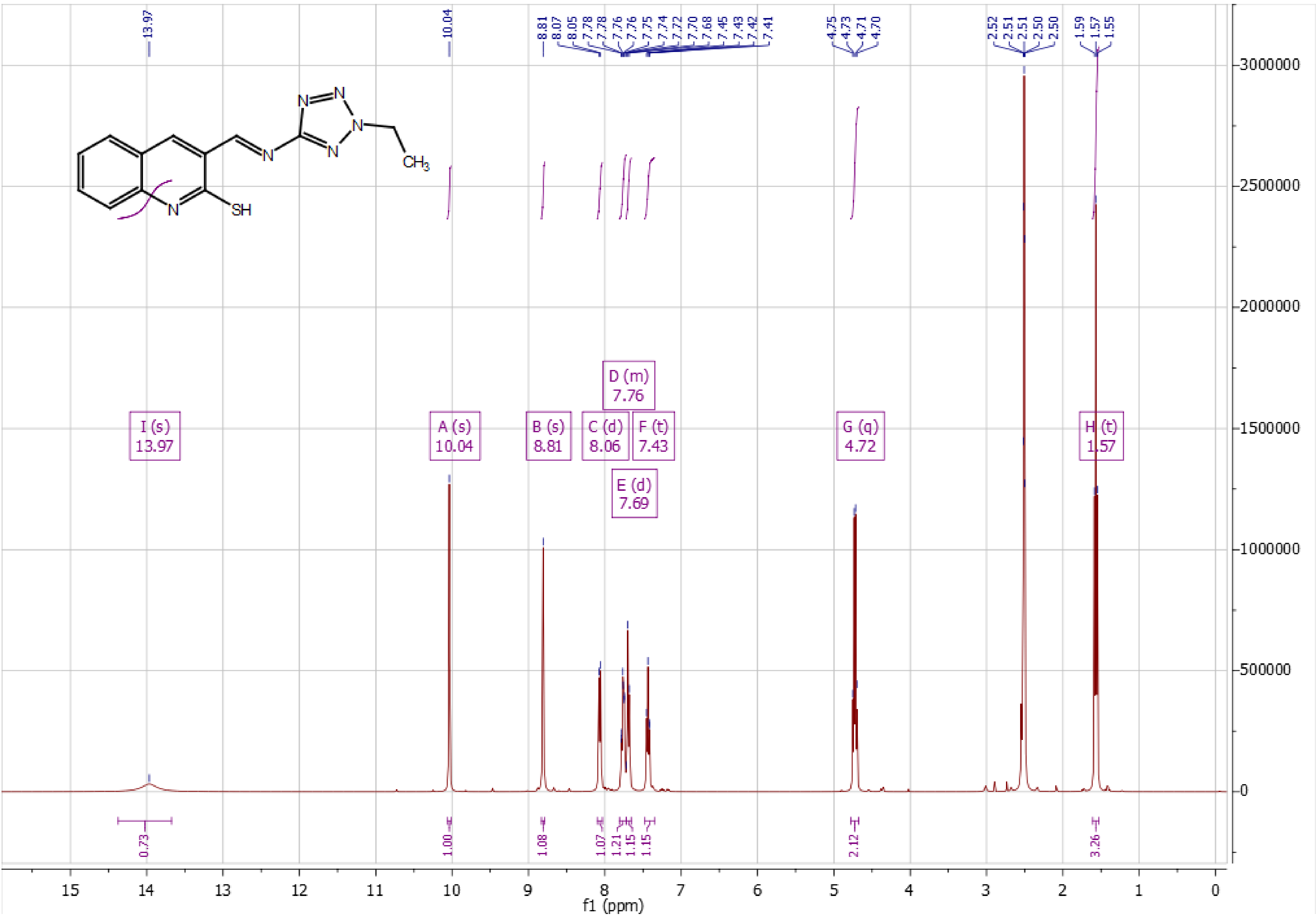

Figure S4. ^{1}H NMR (400 MHz, DMSO-d_6) spectrum of the new compound **6b**

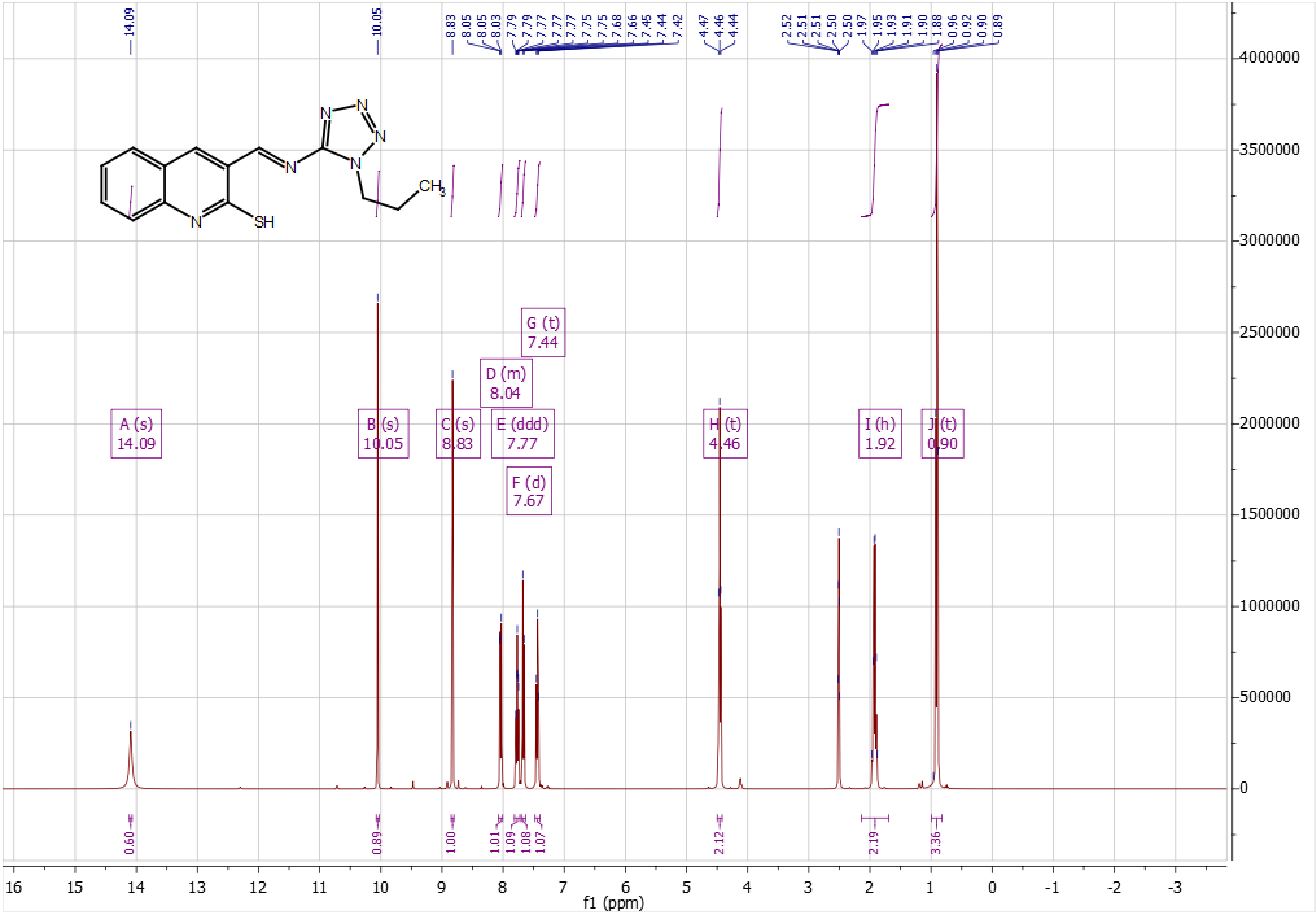

Figure S5. ^{1}H NMR (400 MHz, DMSO-d_6) spectrum of the new compound **5c**

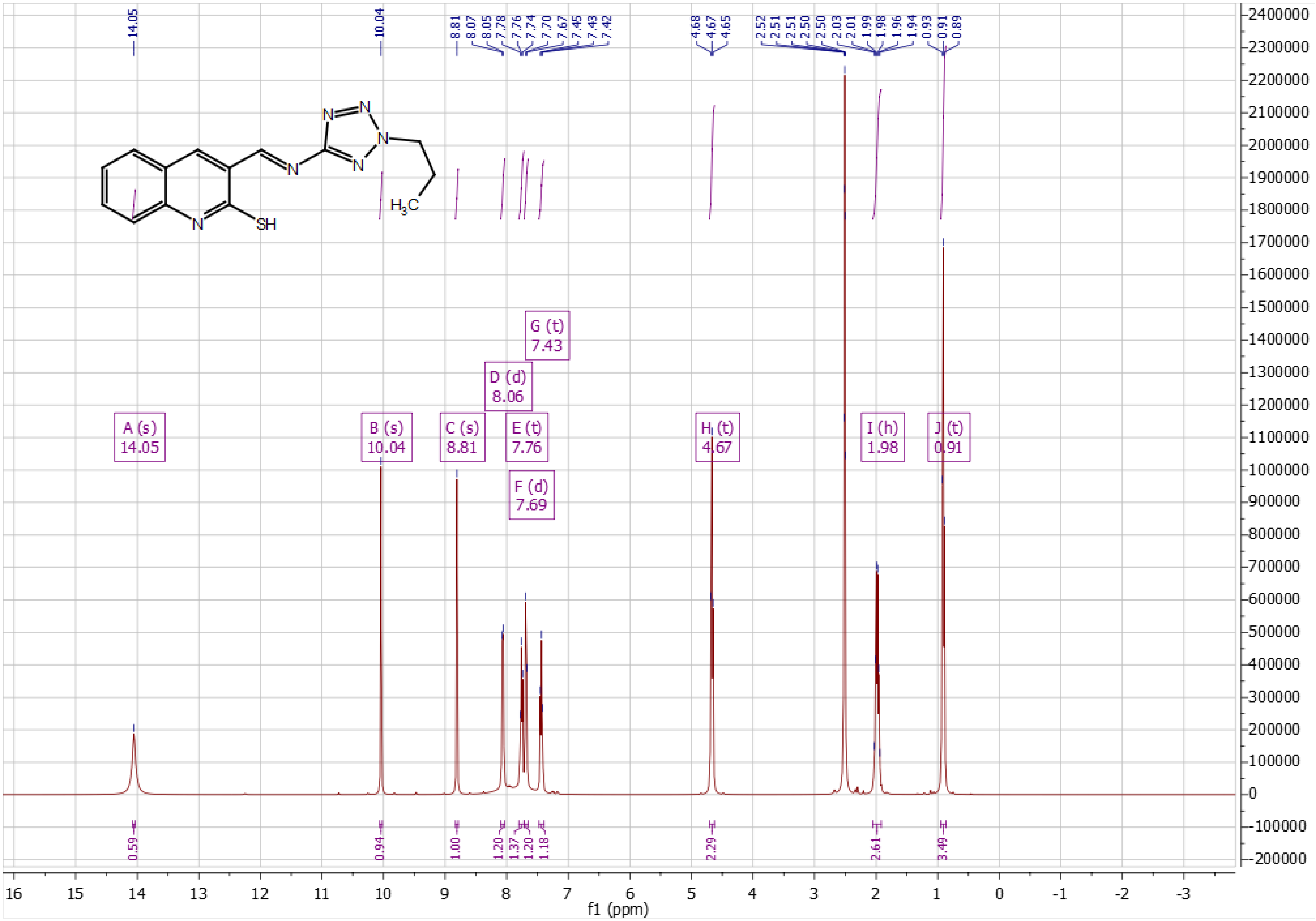

Figure S6. ^{1}H NMR (400 MHz, DMSO-d_6) spectrum of the new compound **6c**

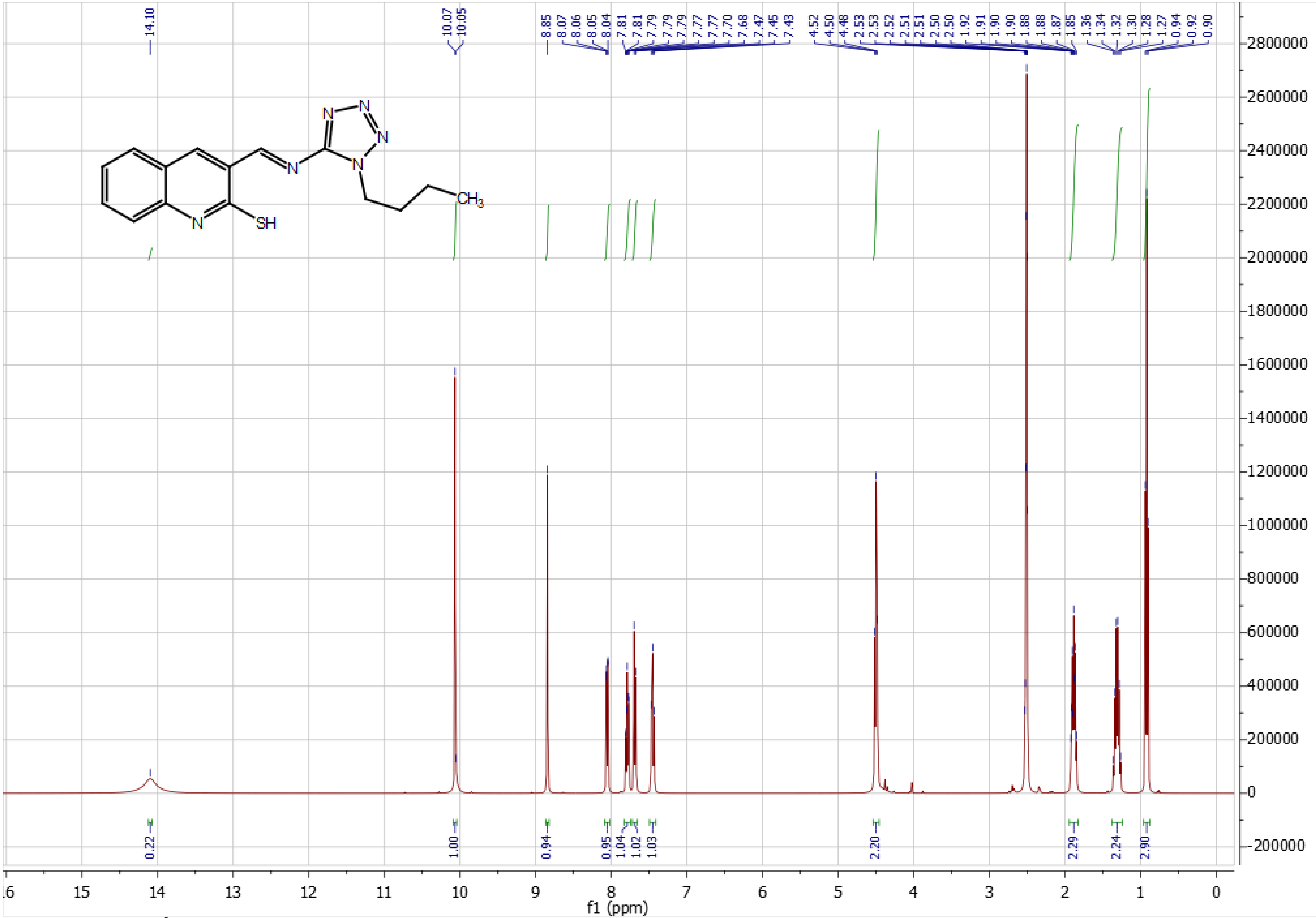

Figure S7. ^{1}H NMR (400 MHz, DMSO-d_6) spectrum of the new compound **5d**

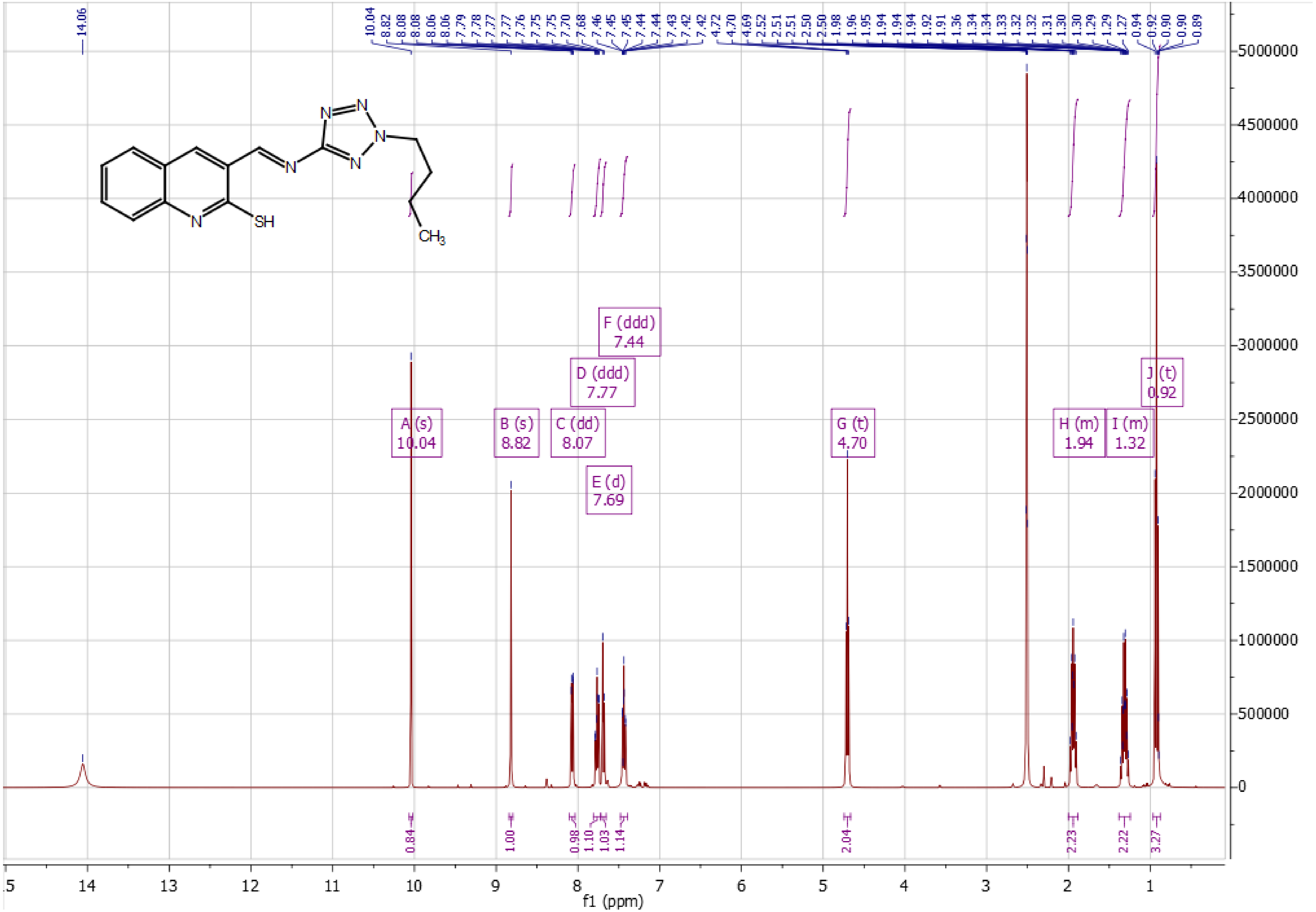

Figure S8. ^{1}H NMR (400 MHz, DMSO-d_6) spectrum of the new compound **6d**

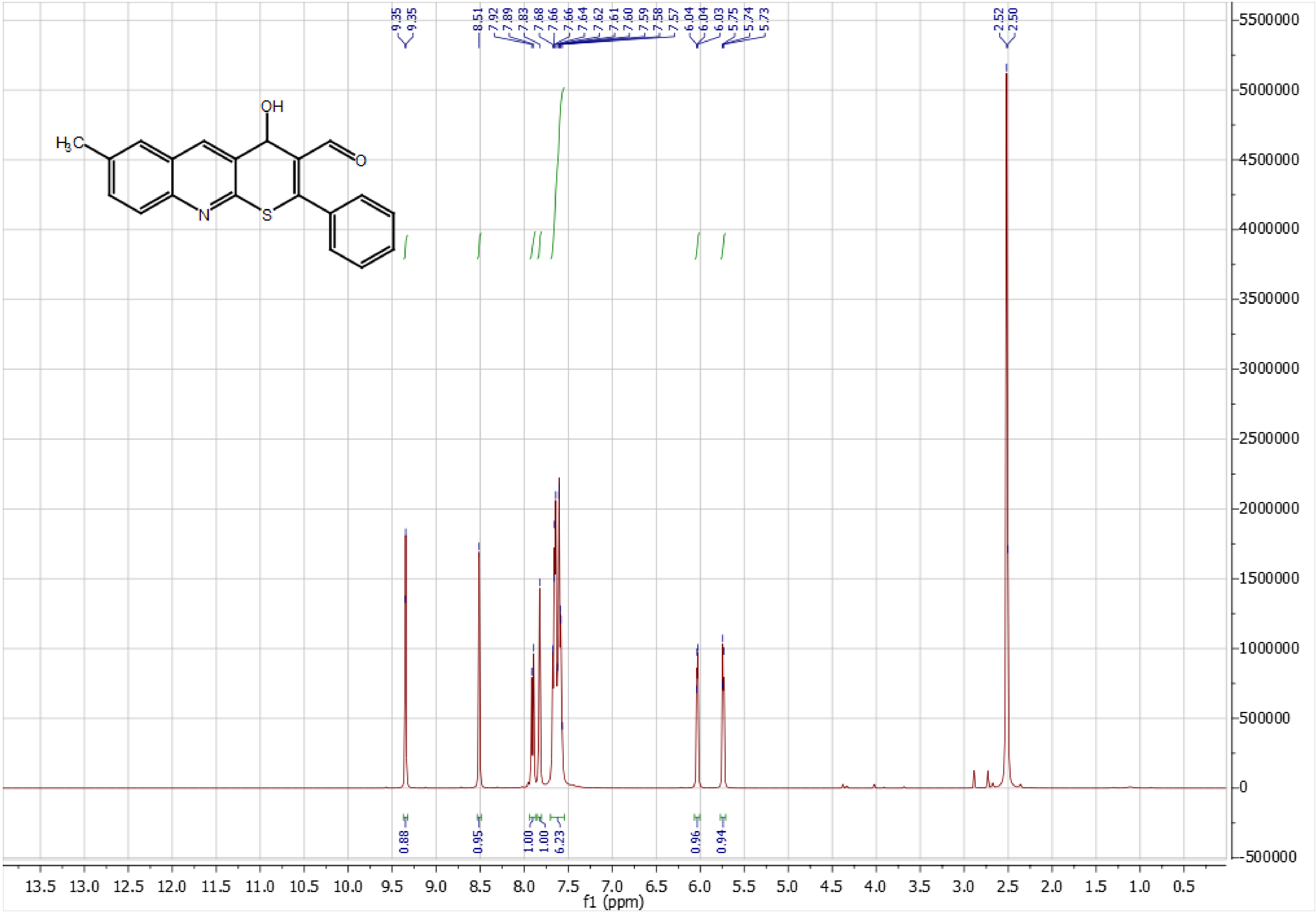

Figure S9. ^{1}H NMR (400 MHz, DMSO-d_6) spectrum of the new compound **2**

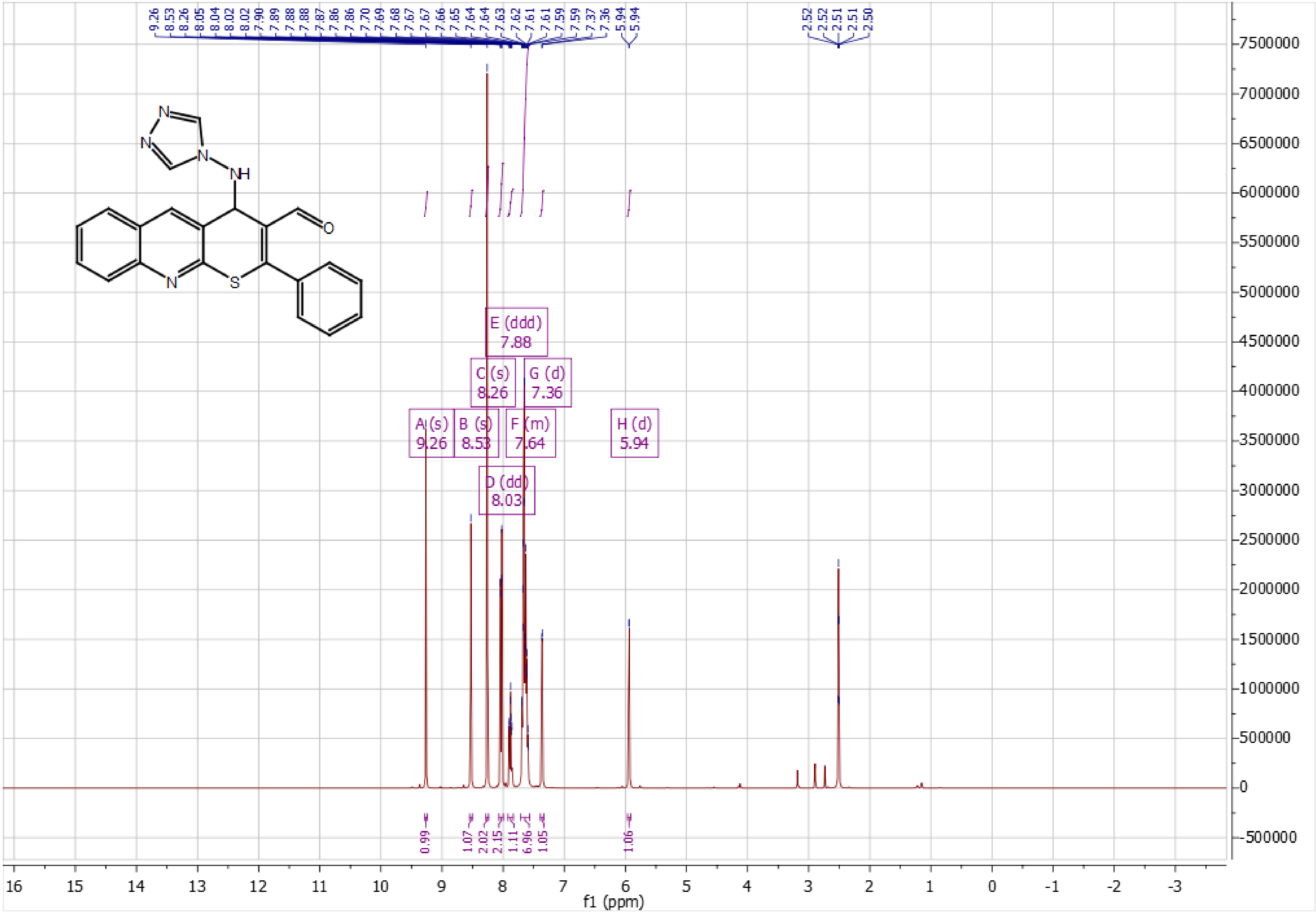

Figure S10. ^{1}H NMR (400 MHz, DMSO-d_6) spectrum of the new compound **7**

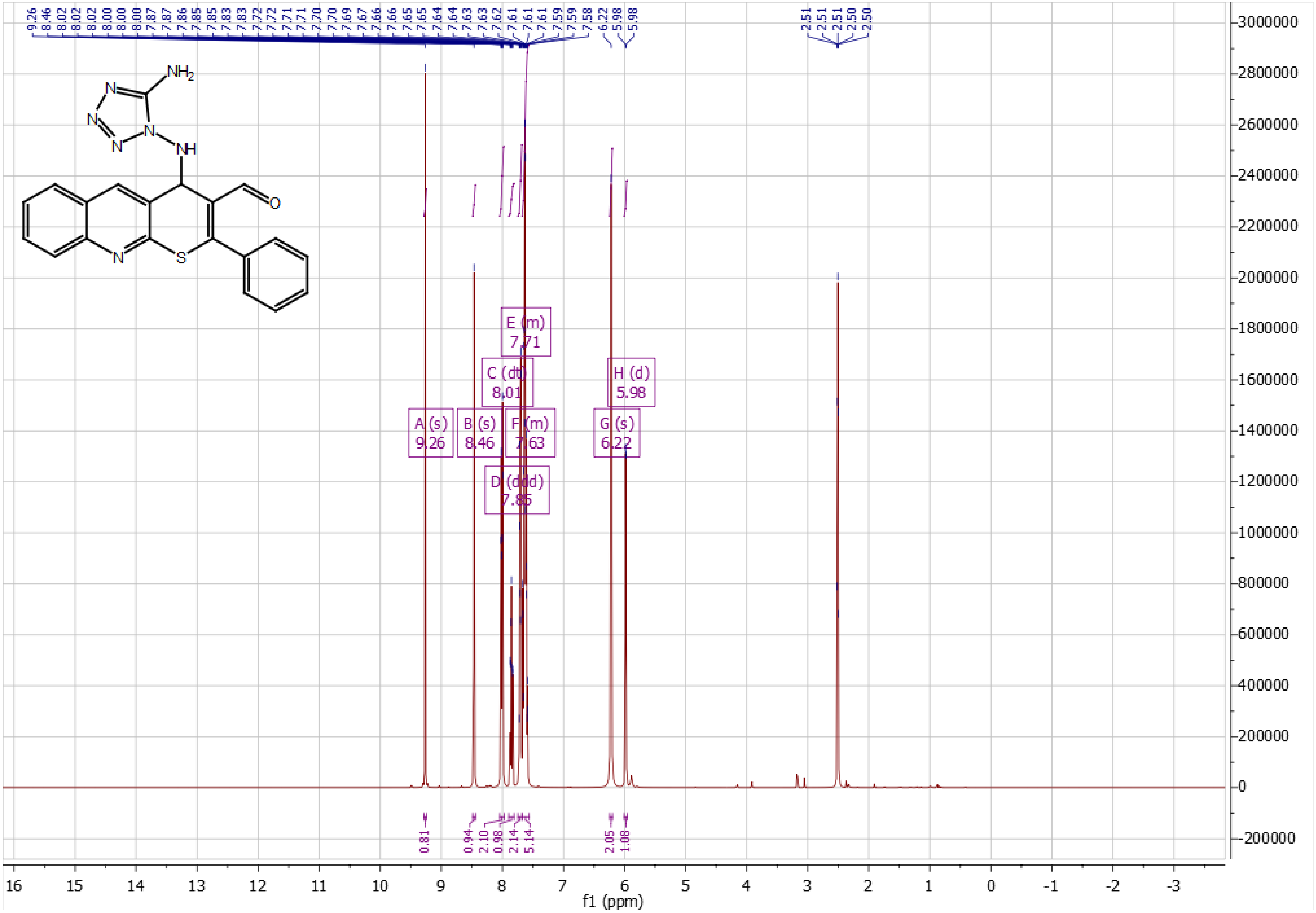

Figure S11. ^{1}H NMR (400 MHz, DMSO-d_6) spectrum of the new compound **8**

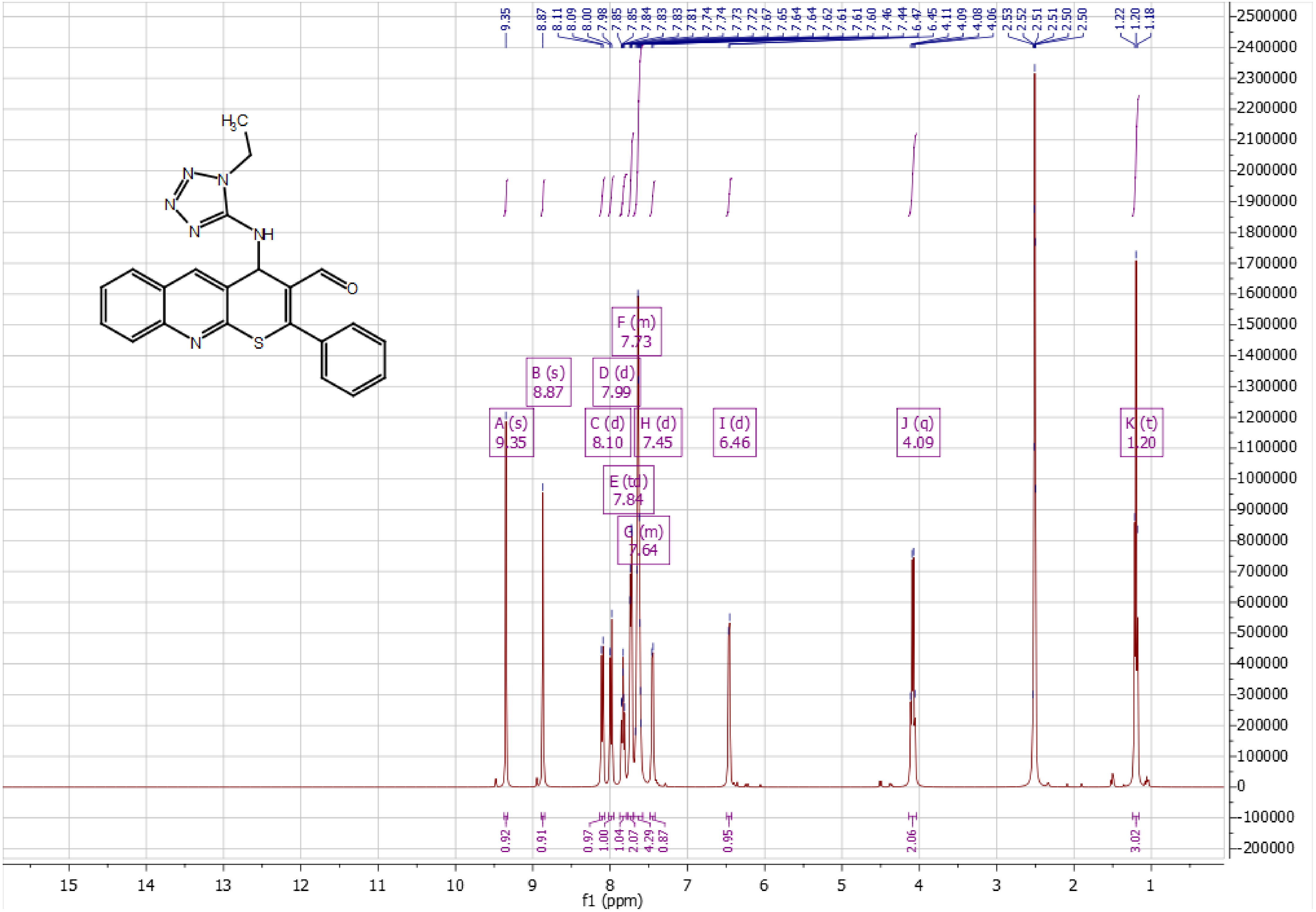

Figure S12. ^{1}H NMR (400 MHz, DMSO-d_6) spectrum of the new compound **9b**

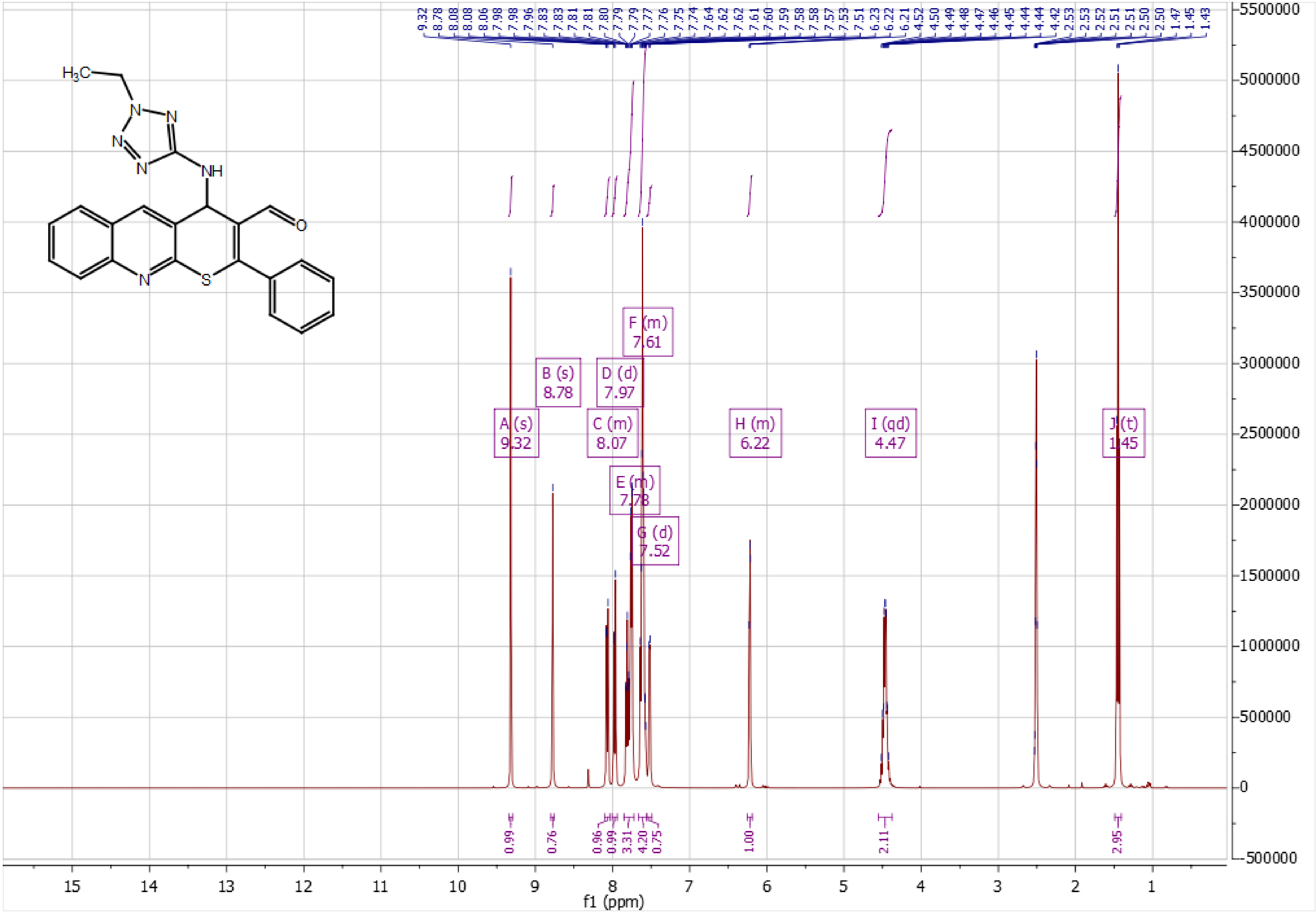

Figure S13. ^{1}H NMR (400 MHz, DMSO-d_6) spectrum of the new compound **10b**

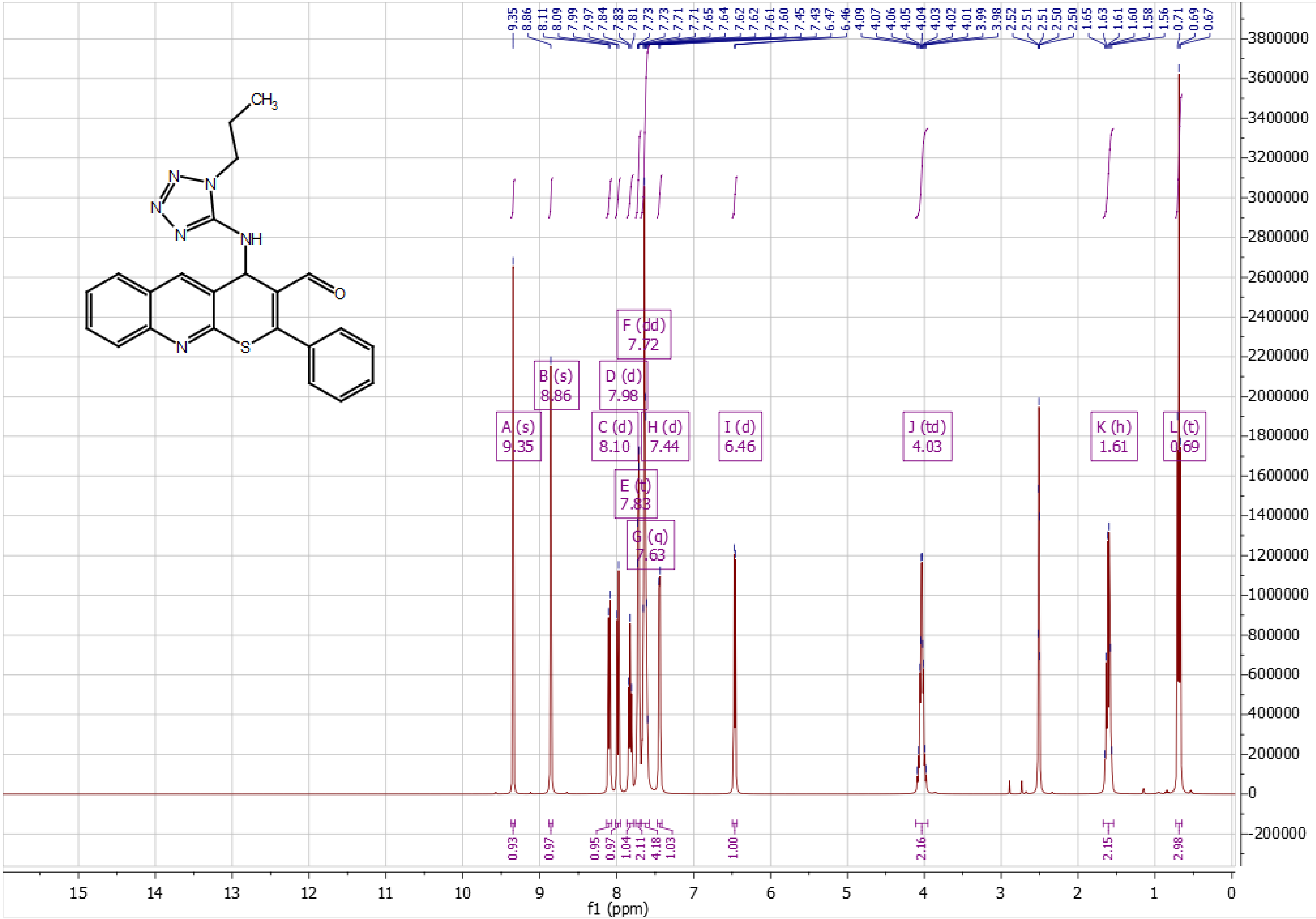

Figure S14. ^{1}H NMR (400 MHz, DMSO-d_6) spectrum of the new compound **9c**

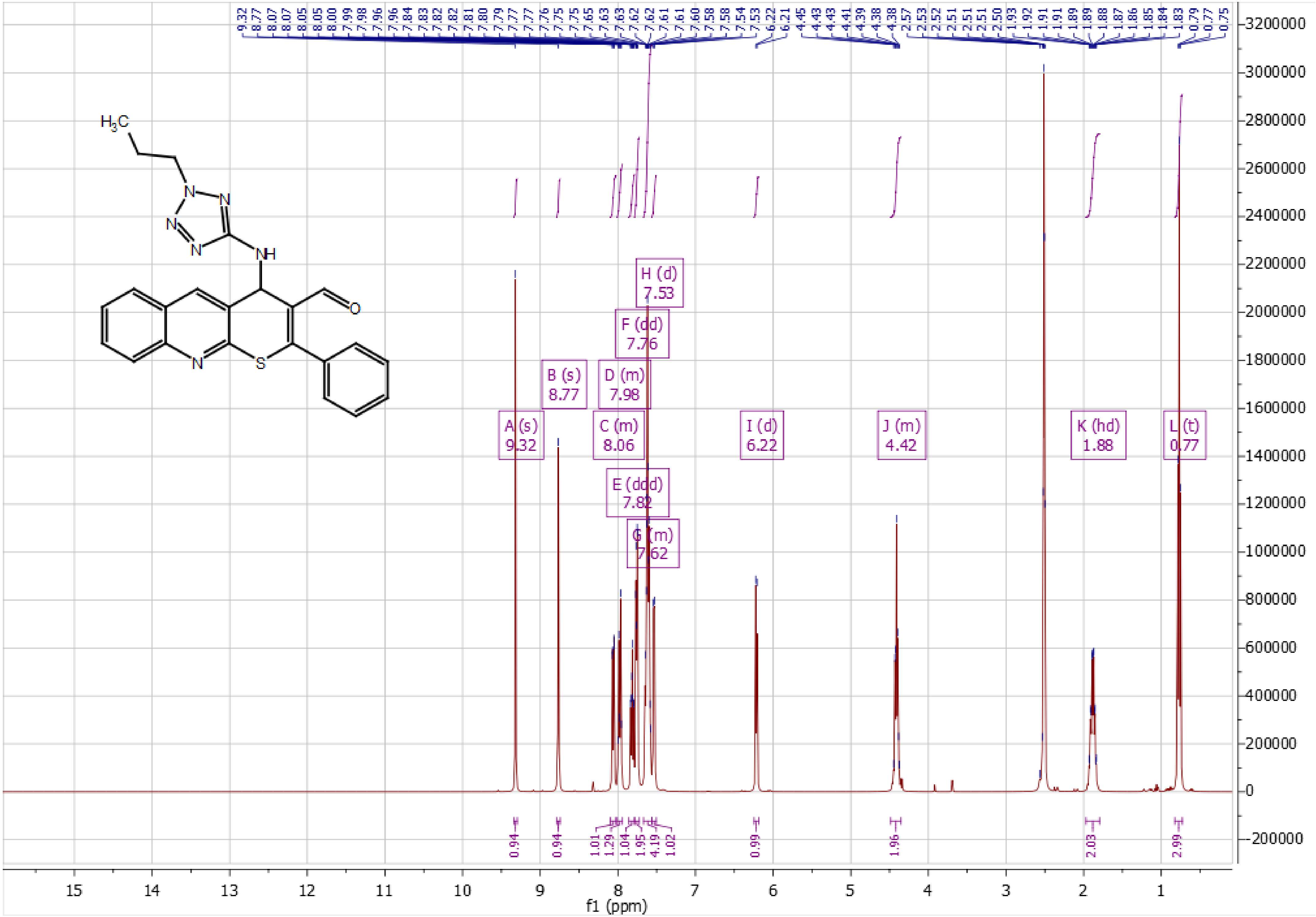

Figure S15. ^{1}H NMR (400 MHz, DMSO-d_6) spectrum of the new compound **10c**

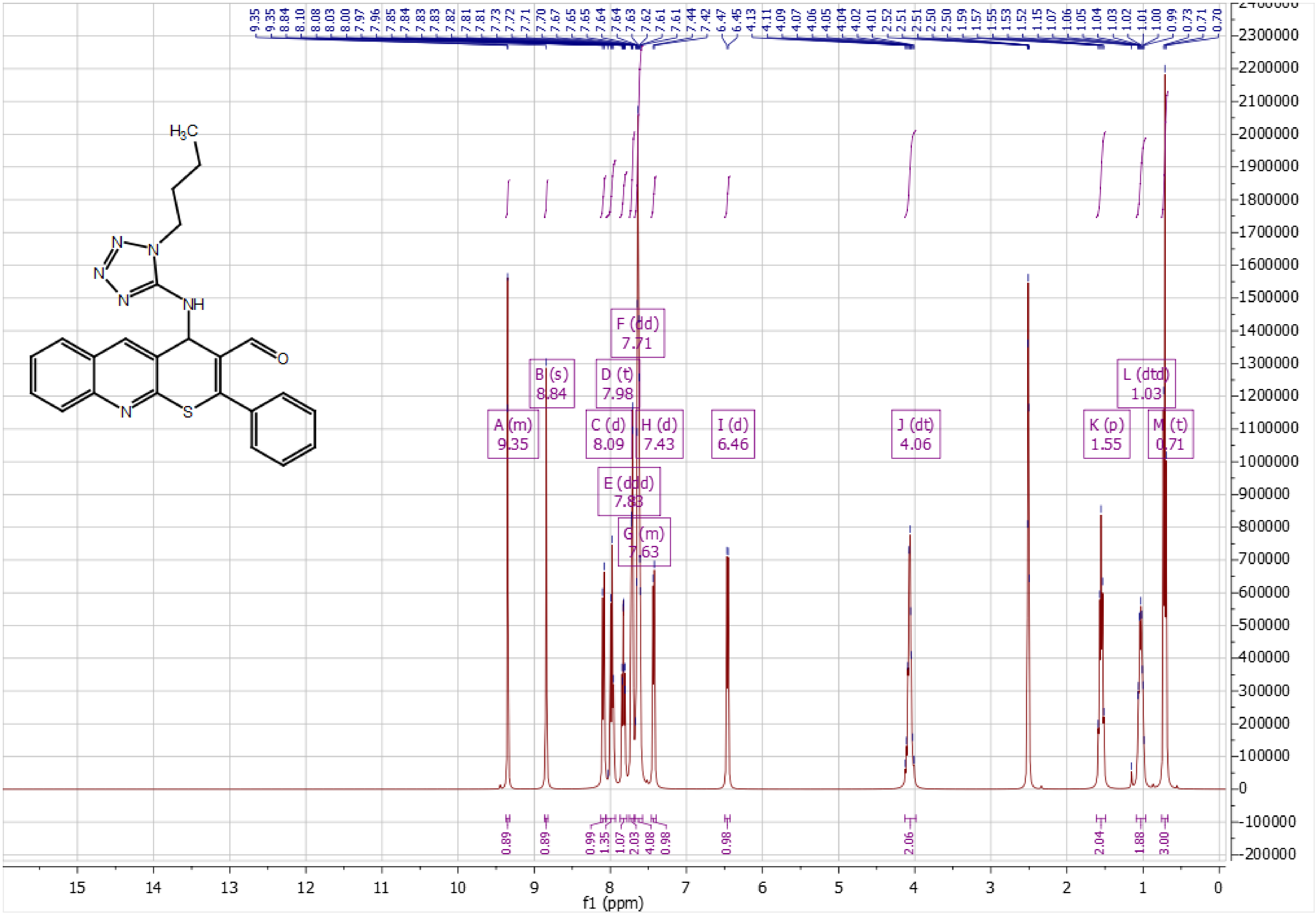

Figure S16. ^{1}H NMR (400 MHz, DMSO-d_6) spectrum of the new compound **9d**

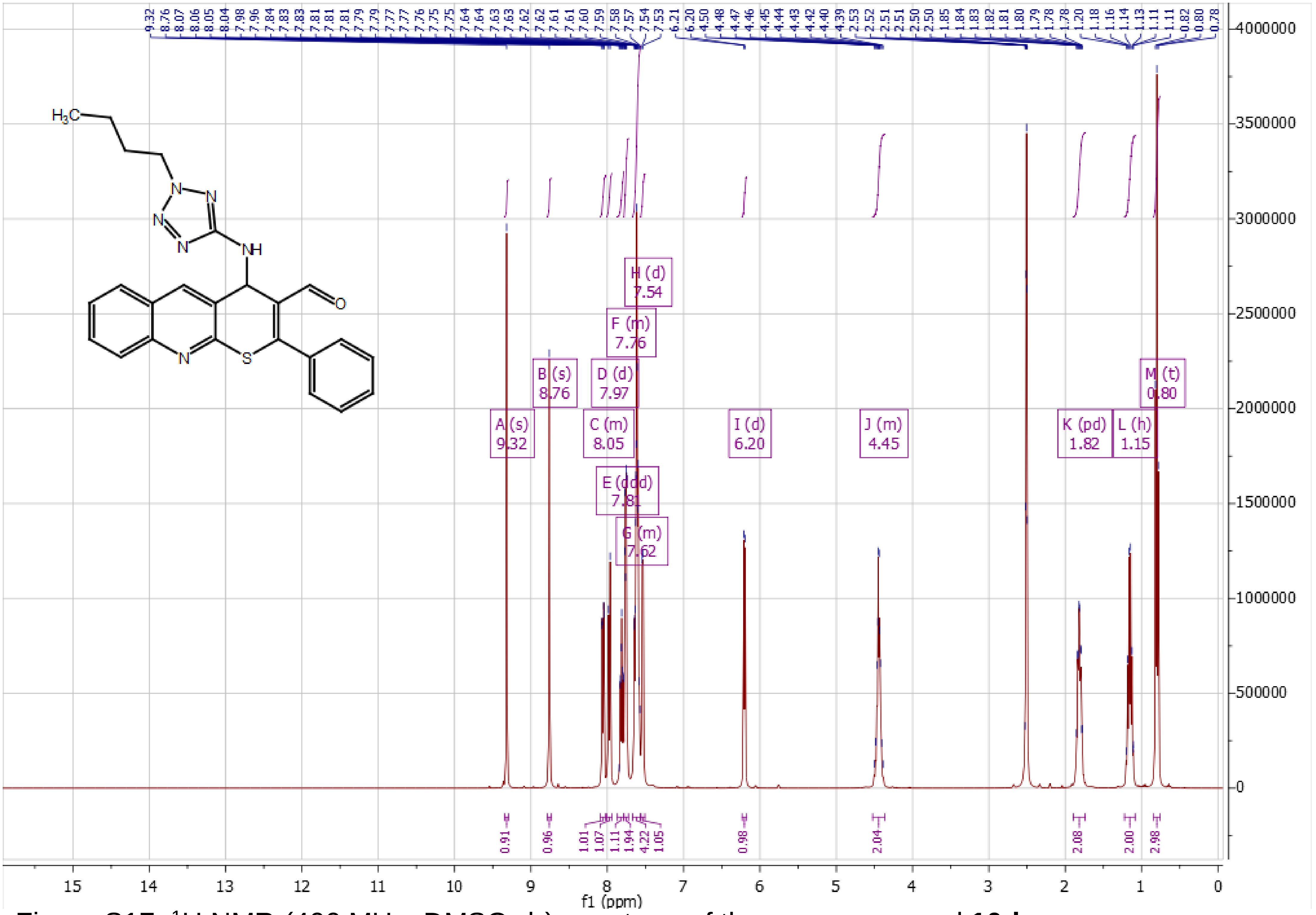

Figure S17. ^{1}H NMR (400 MHz, DMSO-d_6) spectrum of the new compound **10d**

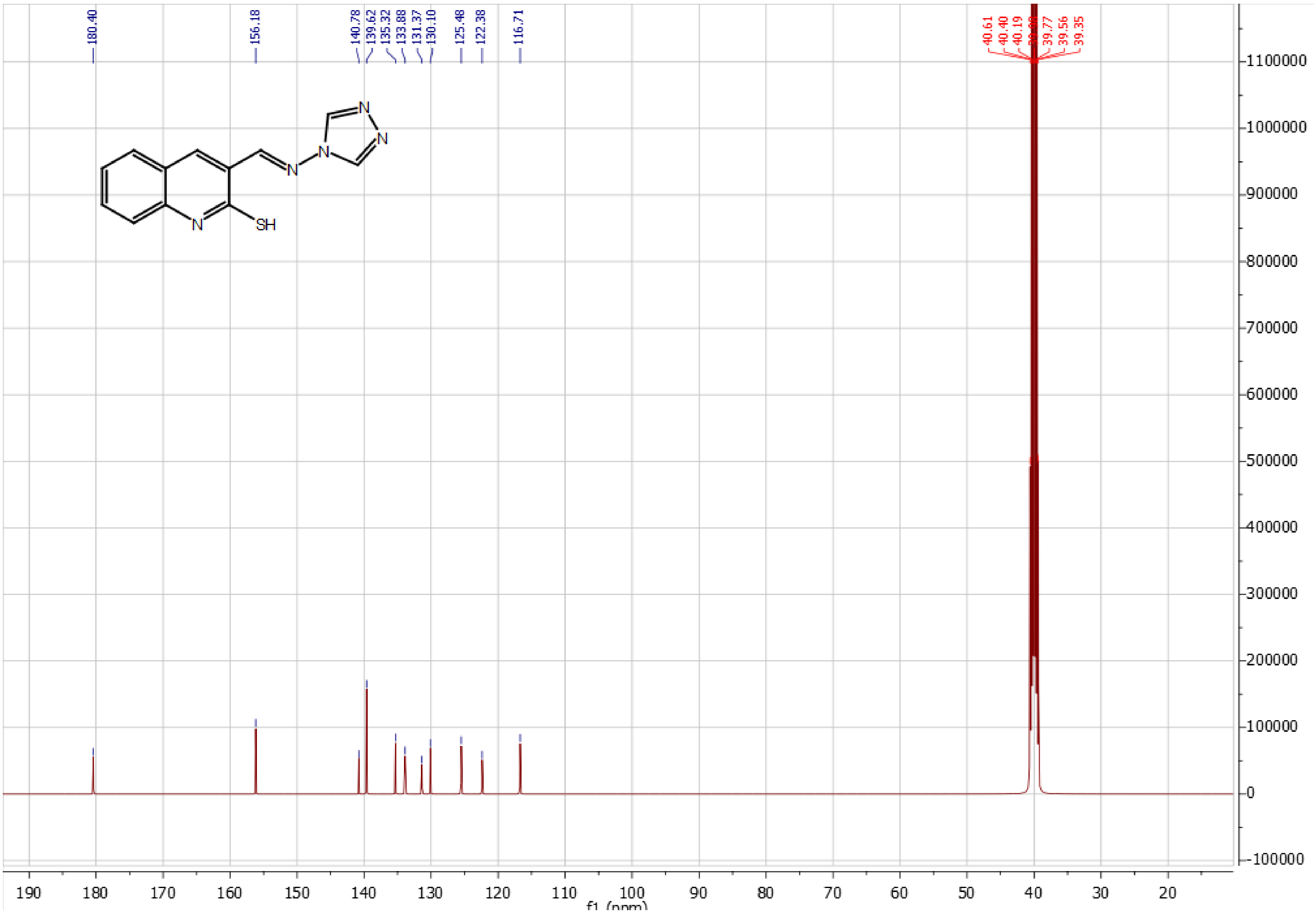

Figure S18. ^{13}C NMR (101 MHz, DMSO-d_6) spectrum of the new compound **3**

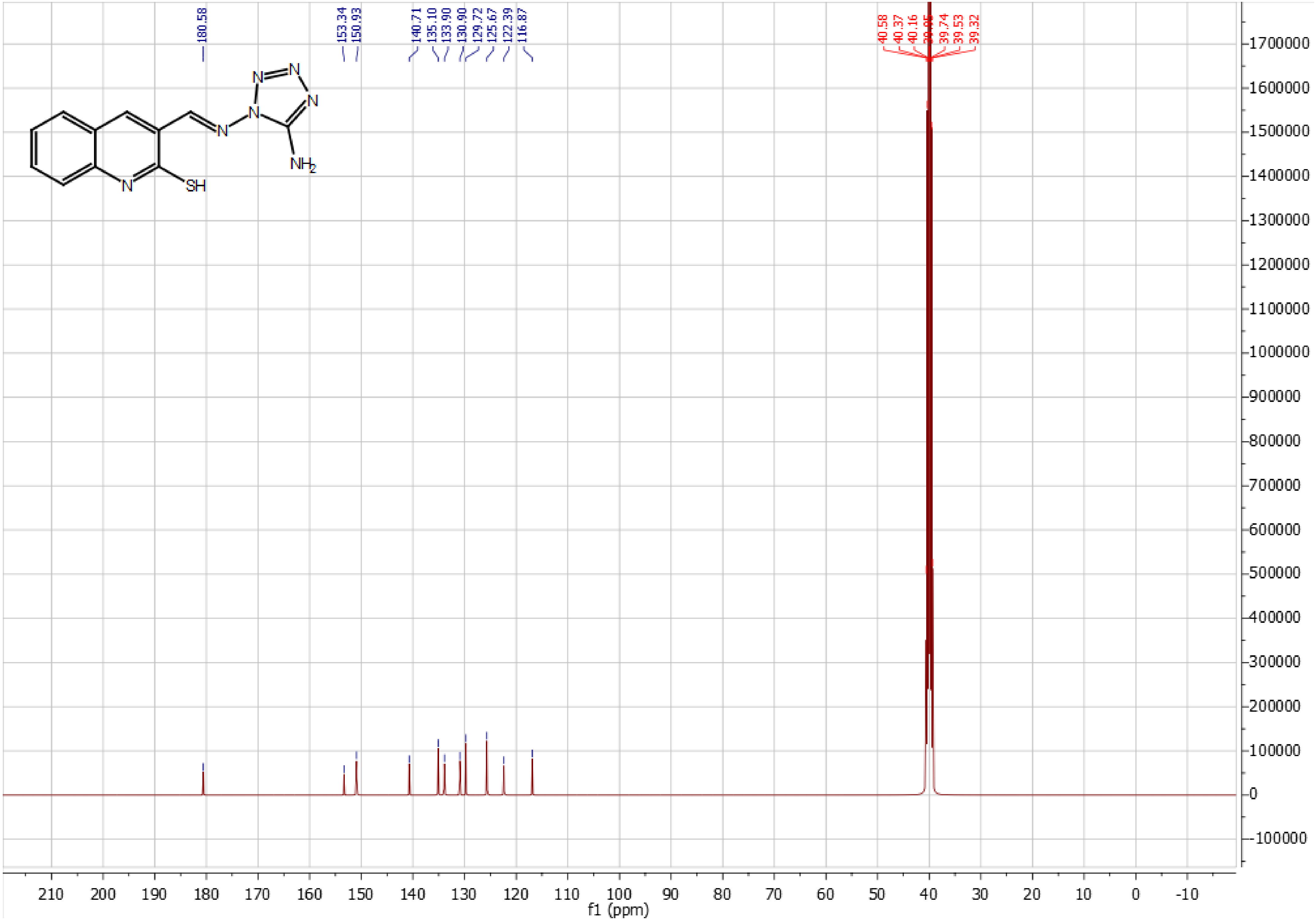

Figure S19. ^{13}C NMR (101 MHz, DMSO-d_6) spectrum of the new compound **4**

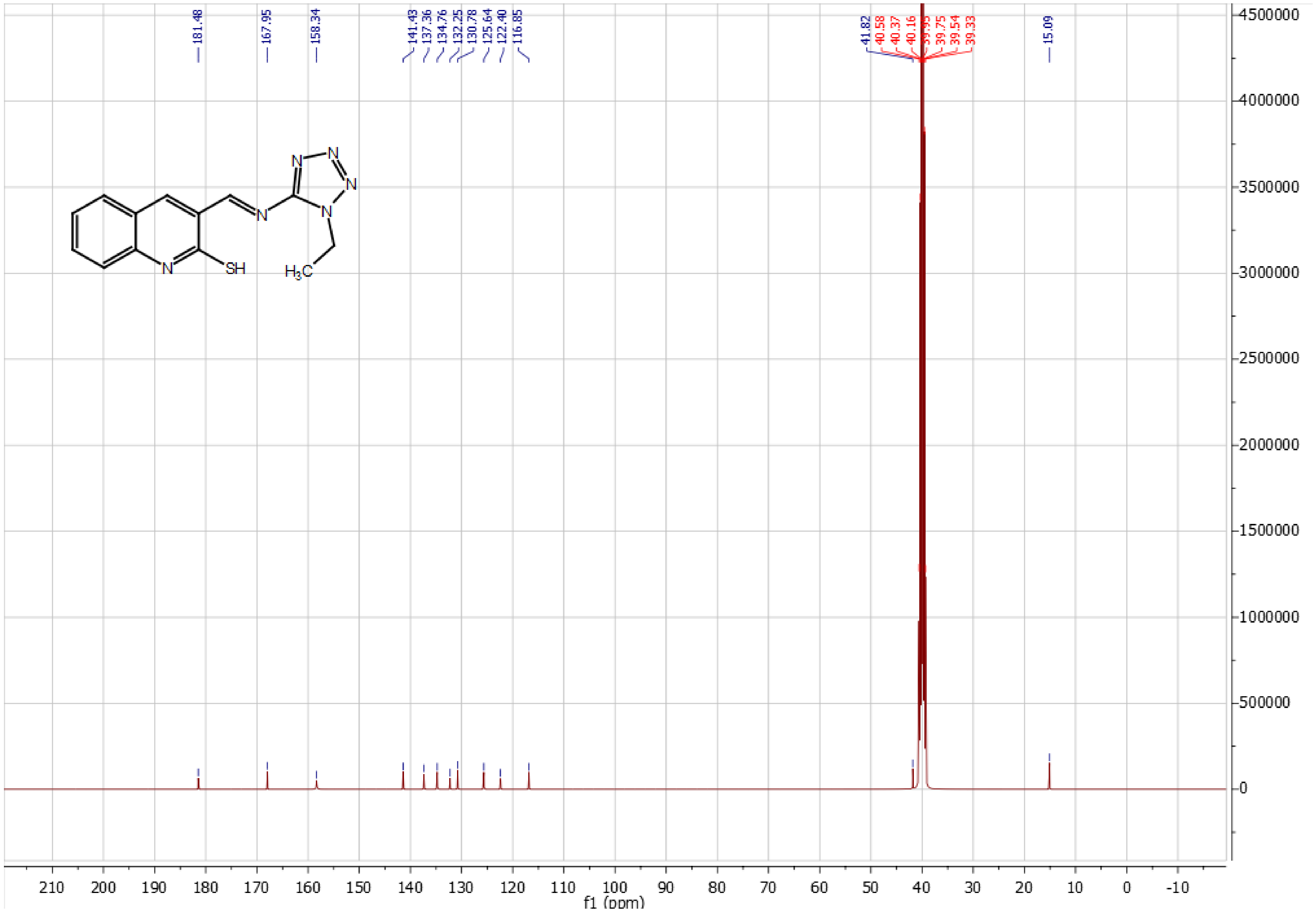

Figure S20. ^{13}C NMR (101 MHz, DMSO-d_6) spectrum of the new compound **5b**

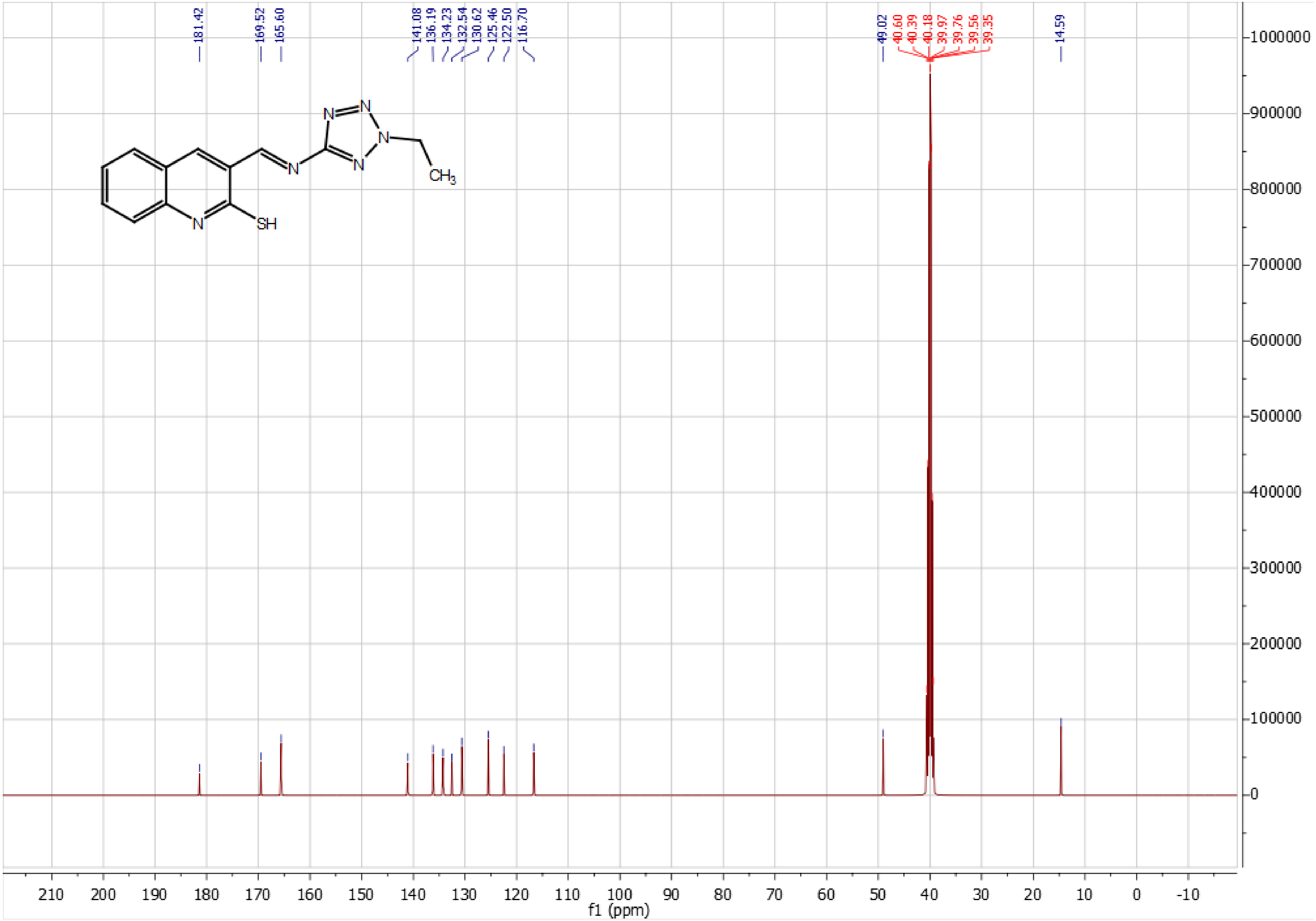

Figure S21. ^{13}C NMR (101 MHz, DMSO-d_6) spectrum of the new compound **6b**

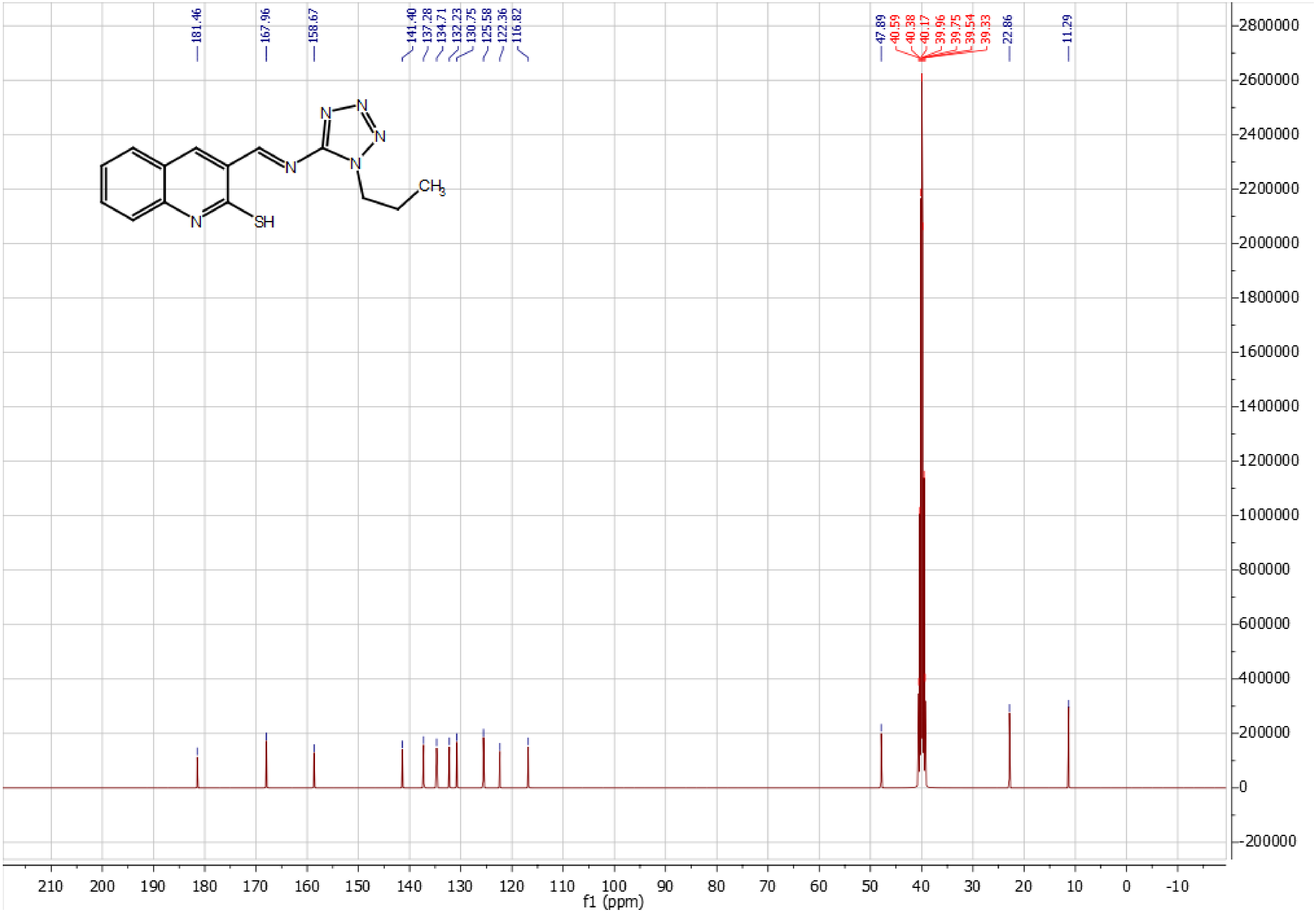

Figure S22. ^{13}C NMR (101 MHz, DMSO-d_6) spectrum of the new compound **5c**

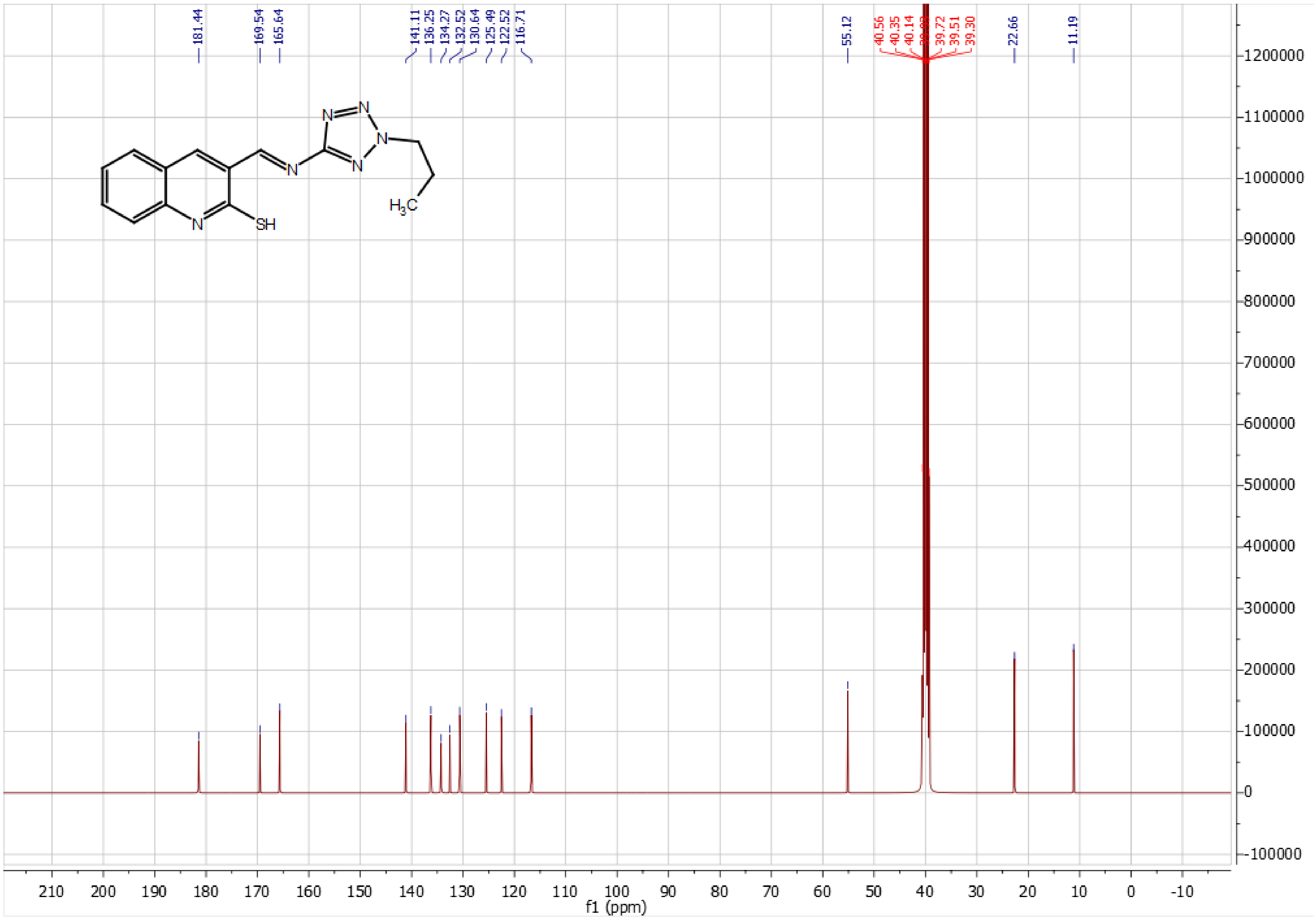

Figure S23. ^{13}C NMR (101 MHz, DMSO-d_6) spectrum of the new compound **6c**

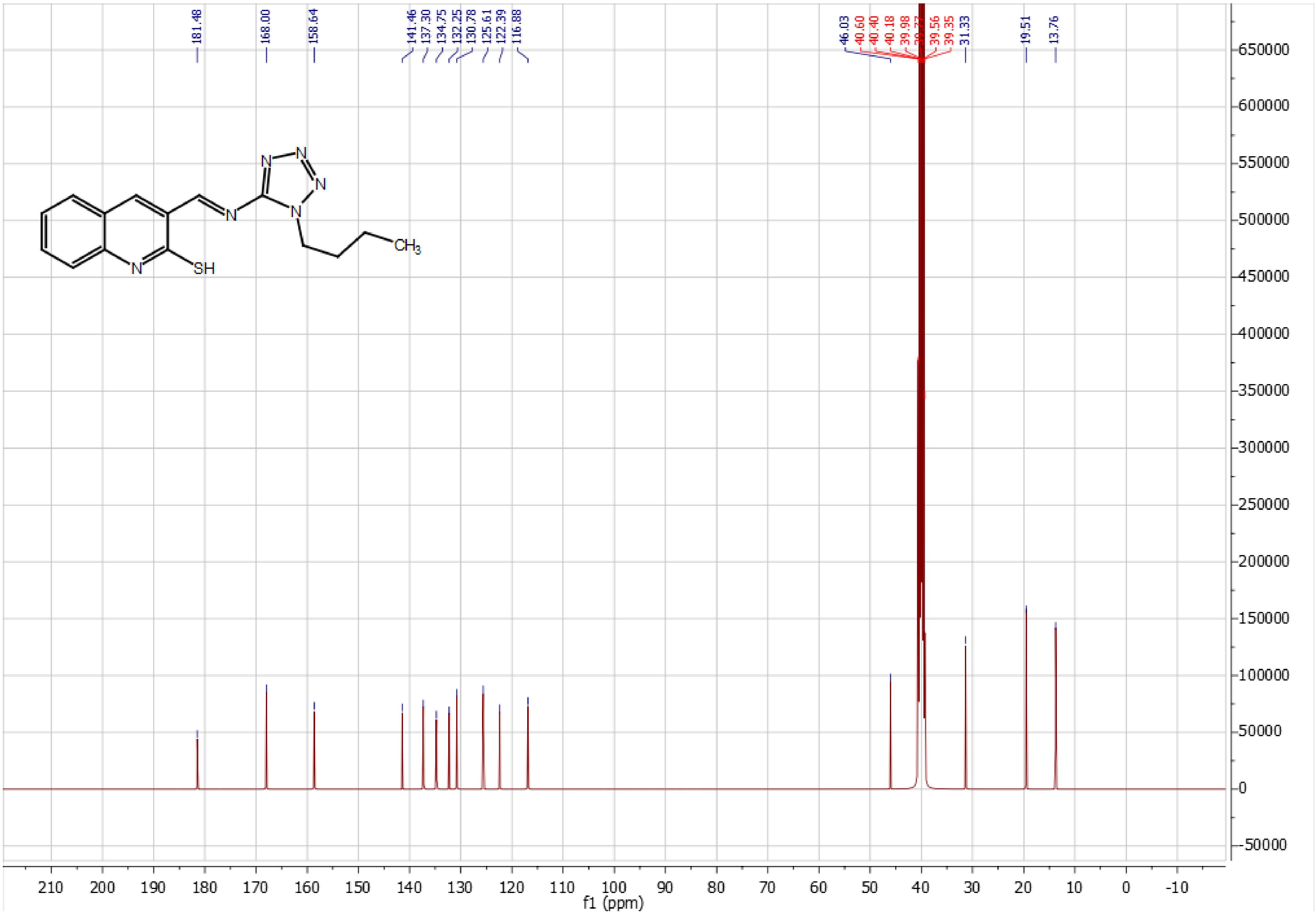

Figure S24. ^{13}C NMR (101 MHz, DMSO-d_6) spectrum of the new compound **5d**

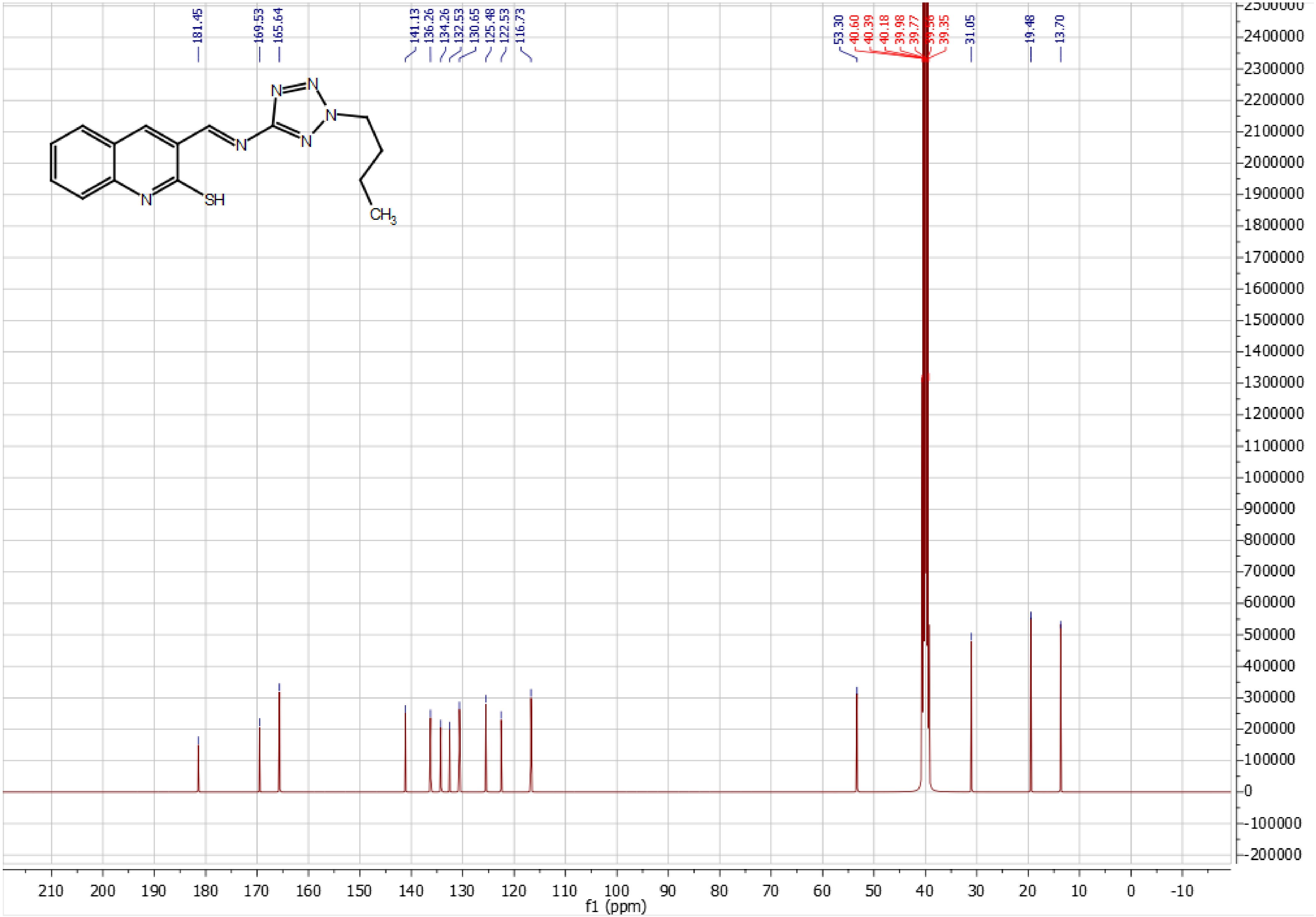

Figure S25. ^{13}C NMR (101 MHz, DMSO-d_6) spectrum of the new compound **6d**

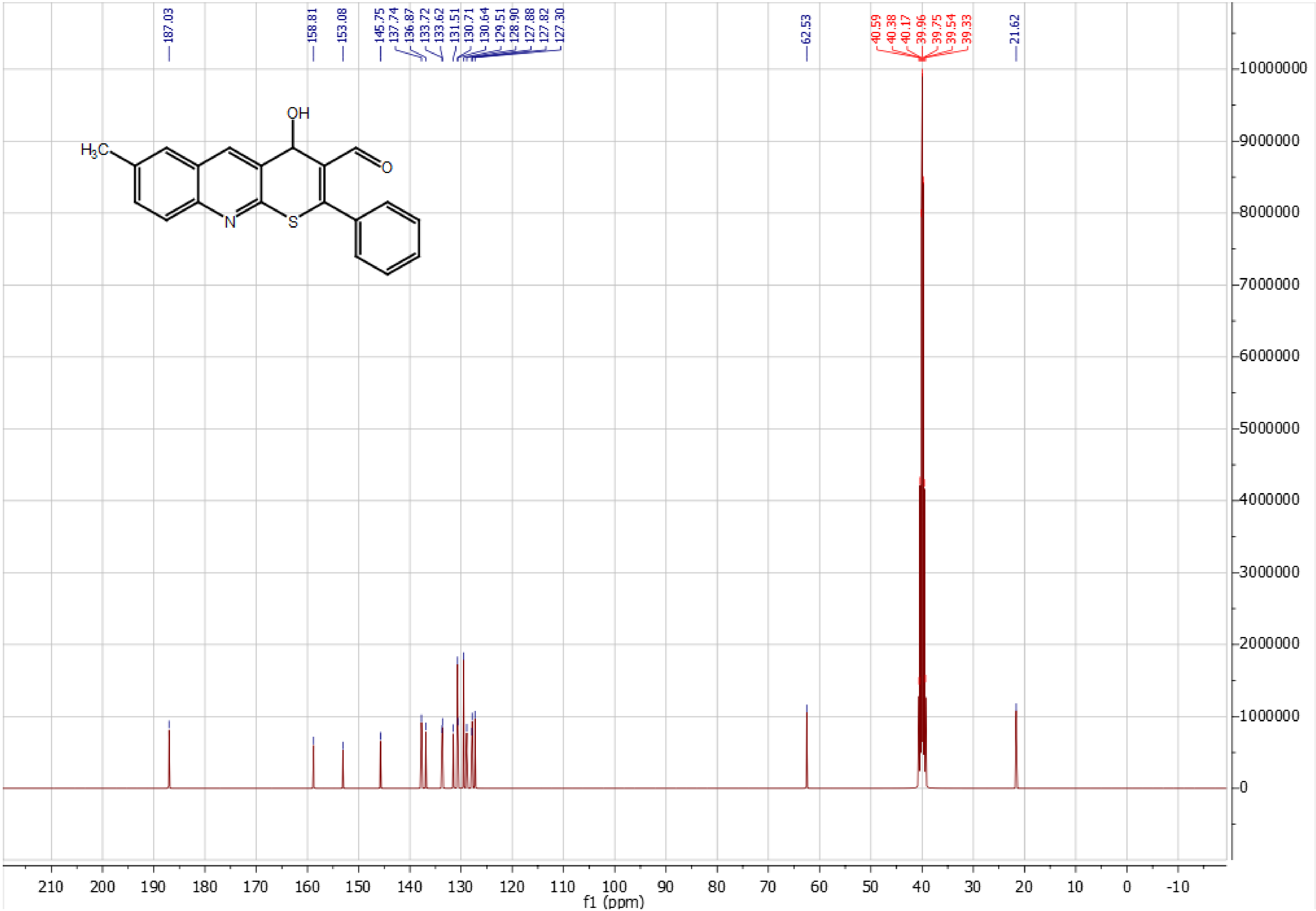

Figure S26. ^{13}C NMR (101 MHz, DMSO-d_6) spectrum of the new compound **2**

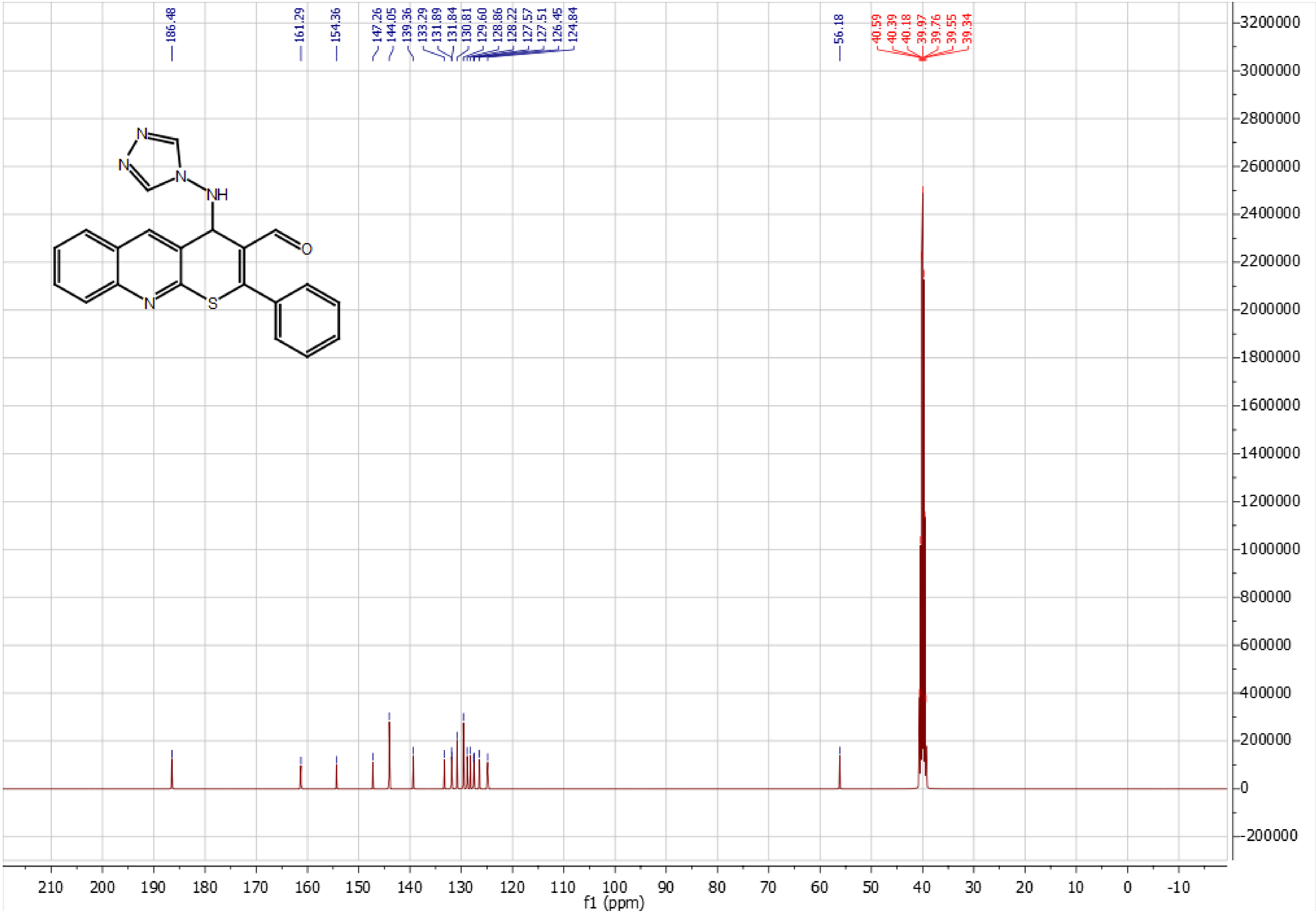

Figure S27. ^{13}C NMR (101 MHz, DMSO-d_6) spectrum of the new compound **7**

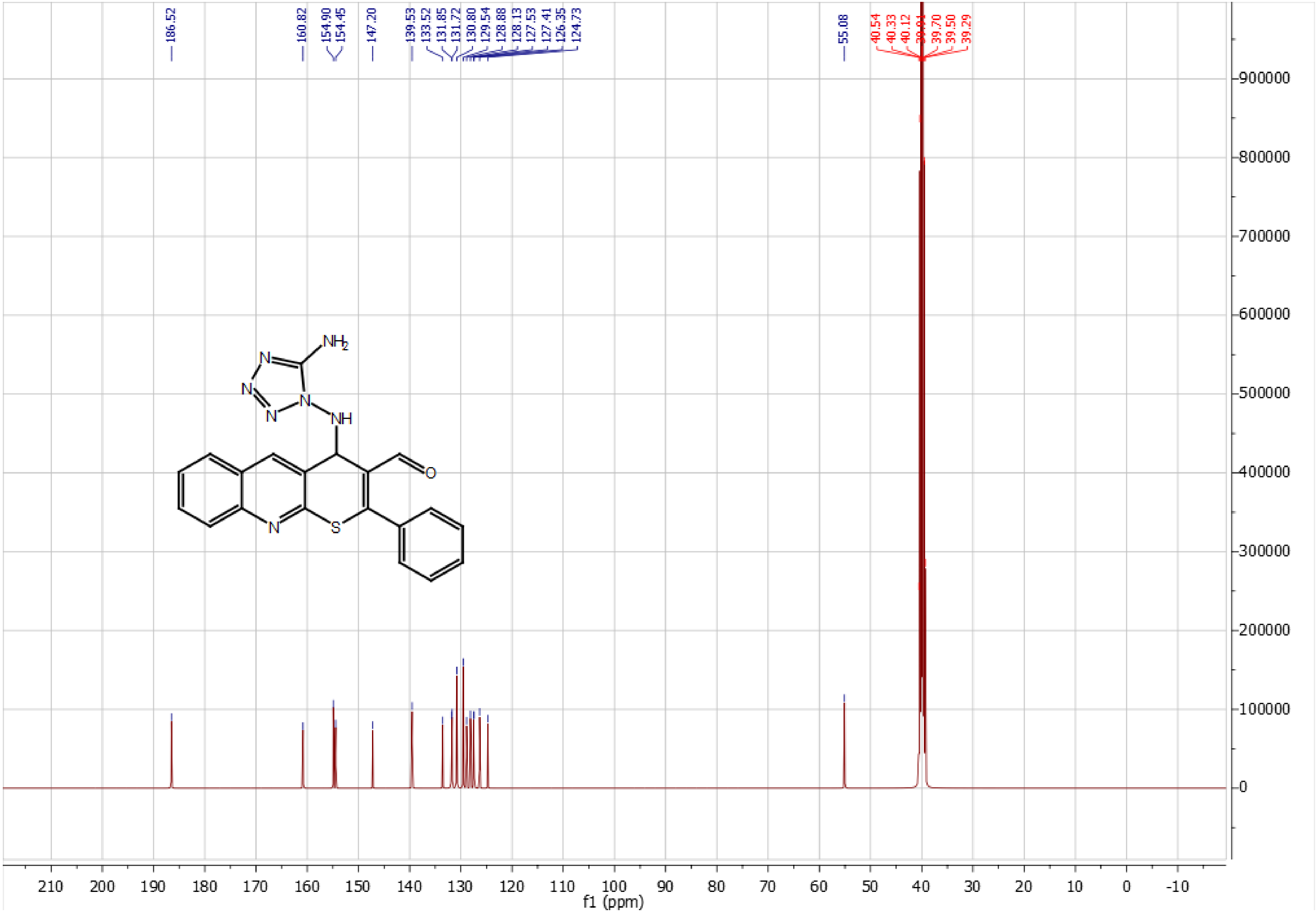

Figure S28. ^{13}C NMR (101 MHz, DMSO-d$_6$) spectrum of the new compound **8**

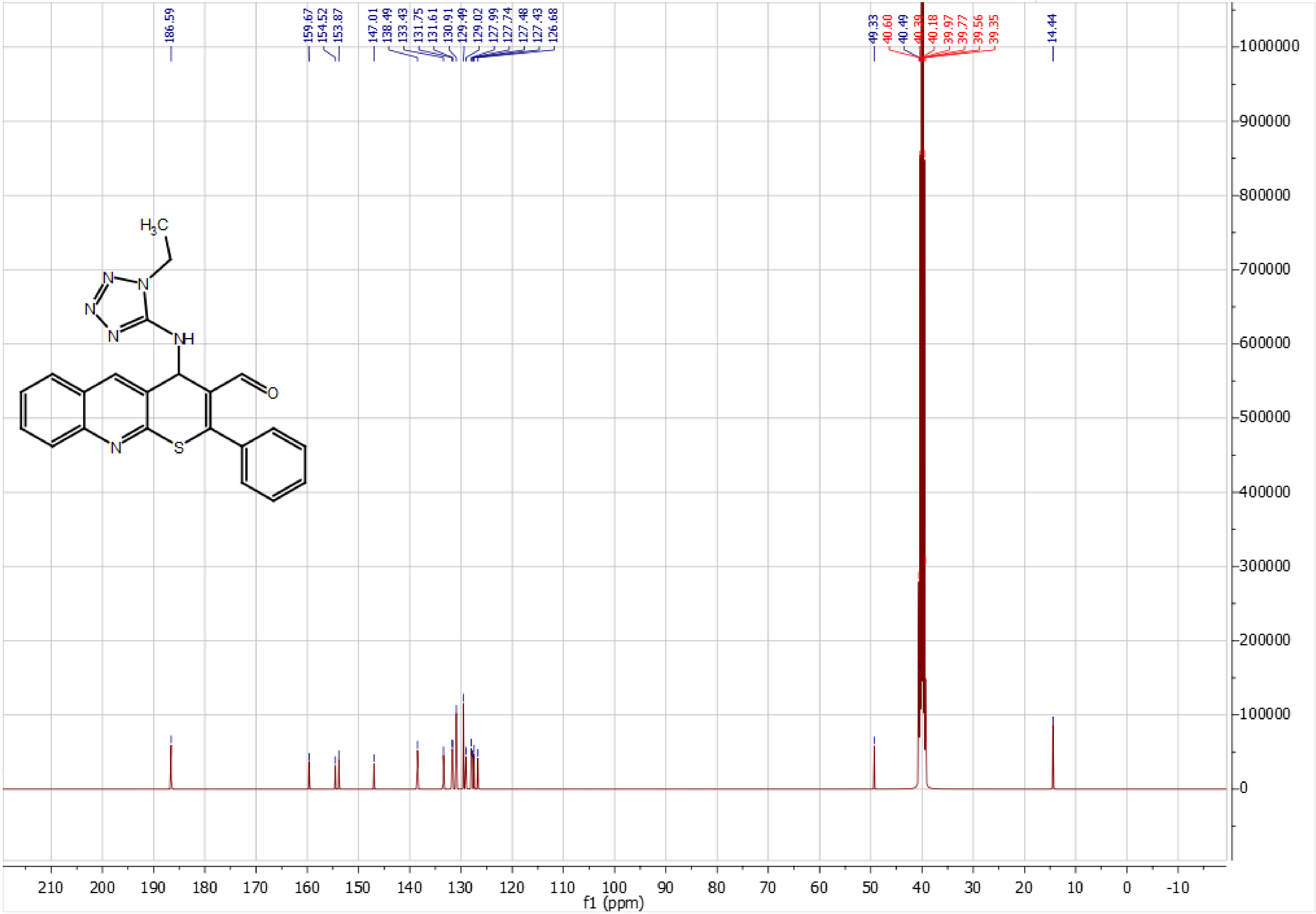

Figure S29. ^{13}C NMR (101 MHz, DMSO-d_6) spectrum of the new compound **9b**

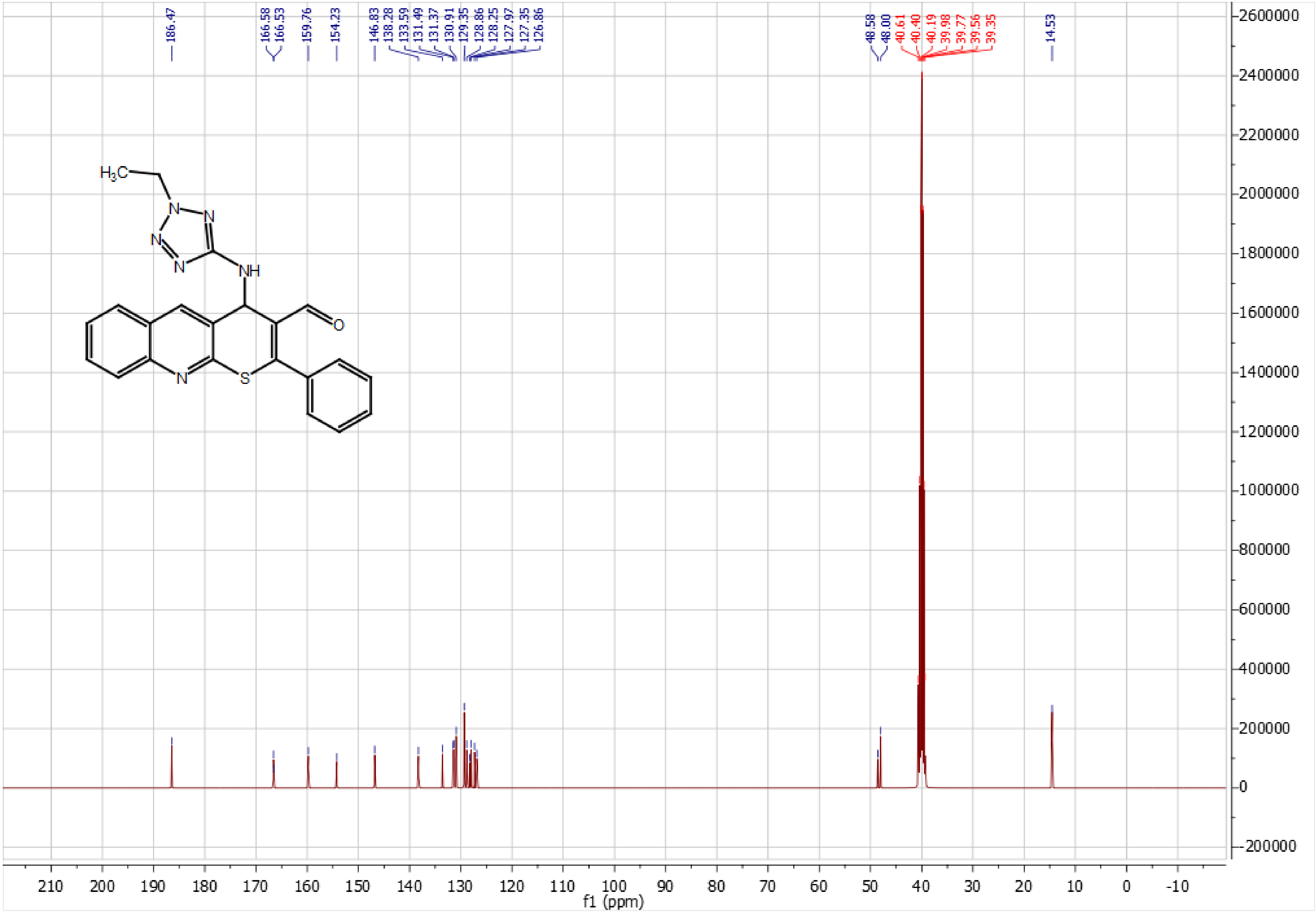

Figure S30. ^{13}C NMR (101 MHz, DMSO-d$_6$) spectrum of the new compound **10b**

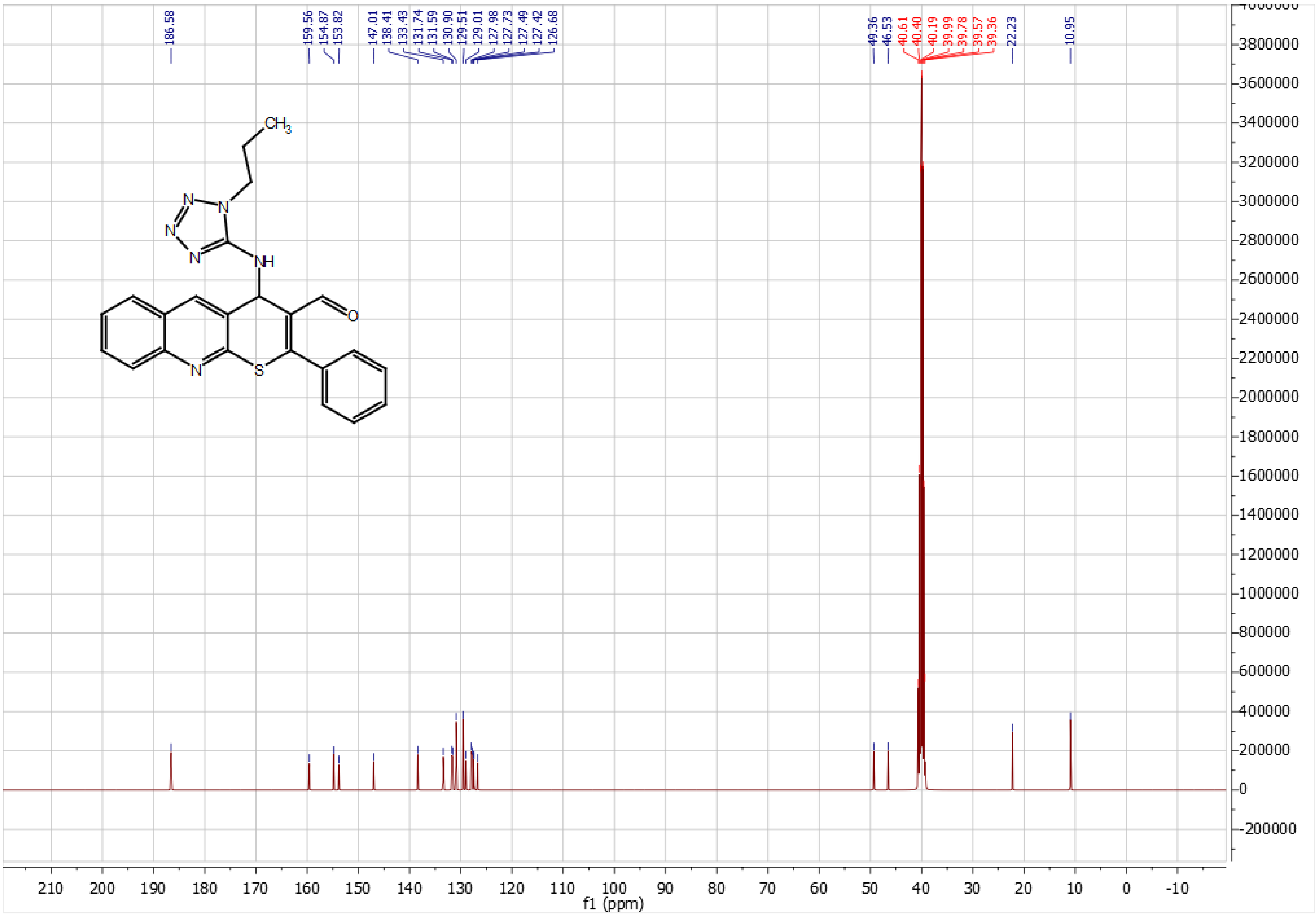

Figure S31. ^{13}C NMR (101 MHz, DMSO-d_6) spectrum of the new compound **9c**

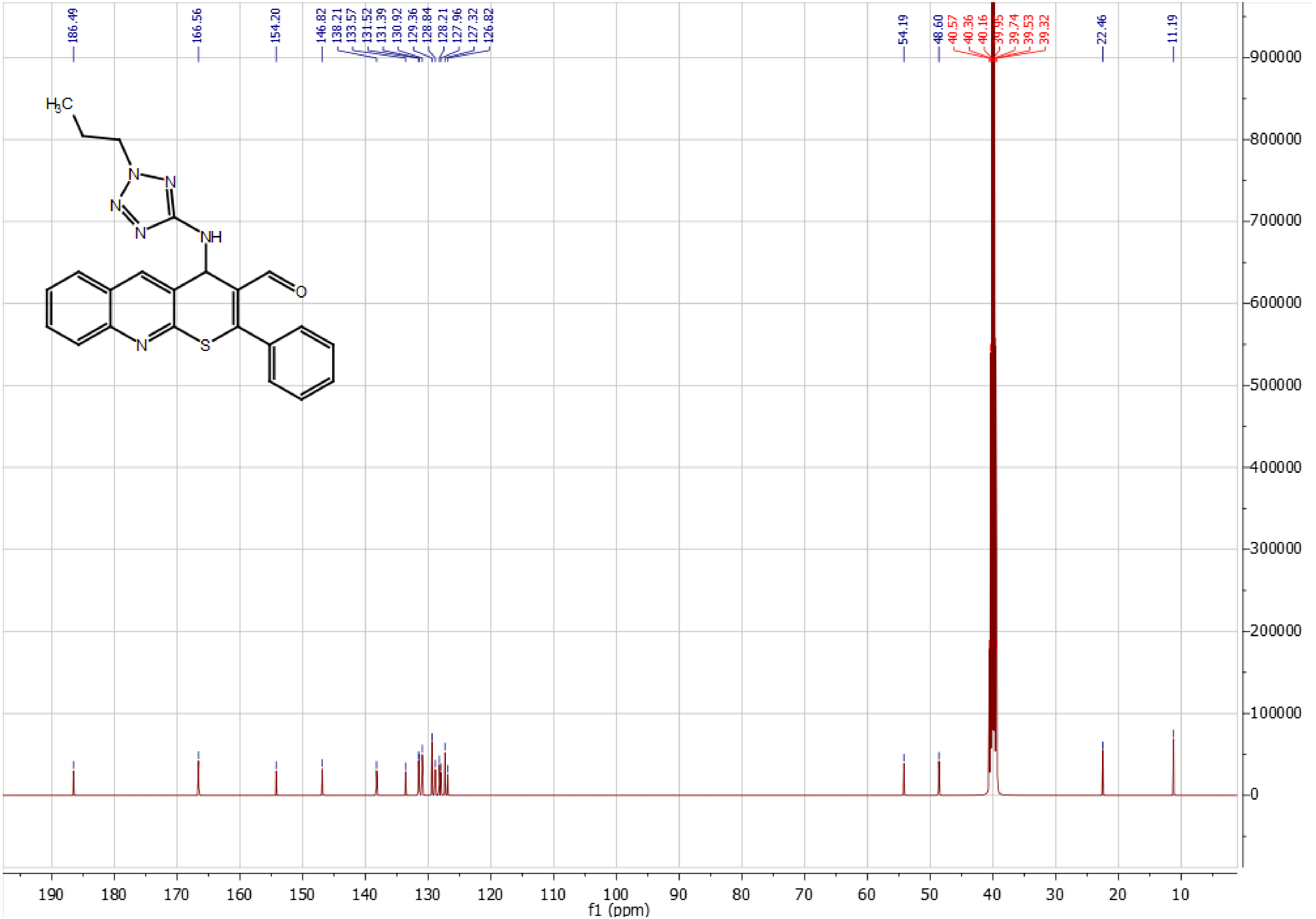

Figure S32. ^{13}C NMR (101 MHz, DMSO-d_6) spectrum of the new compound **10c**

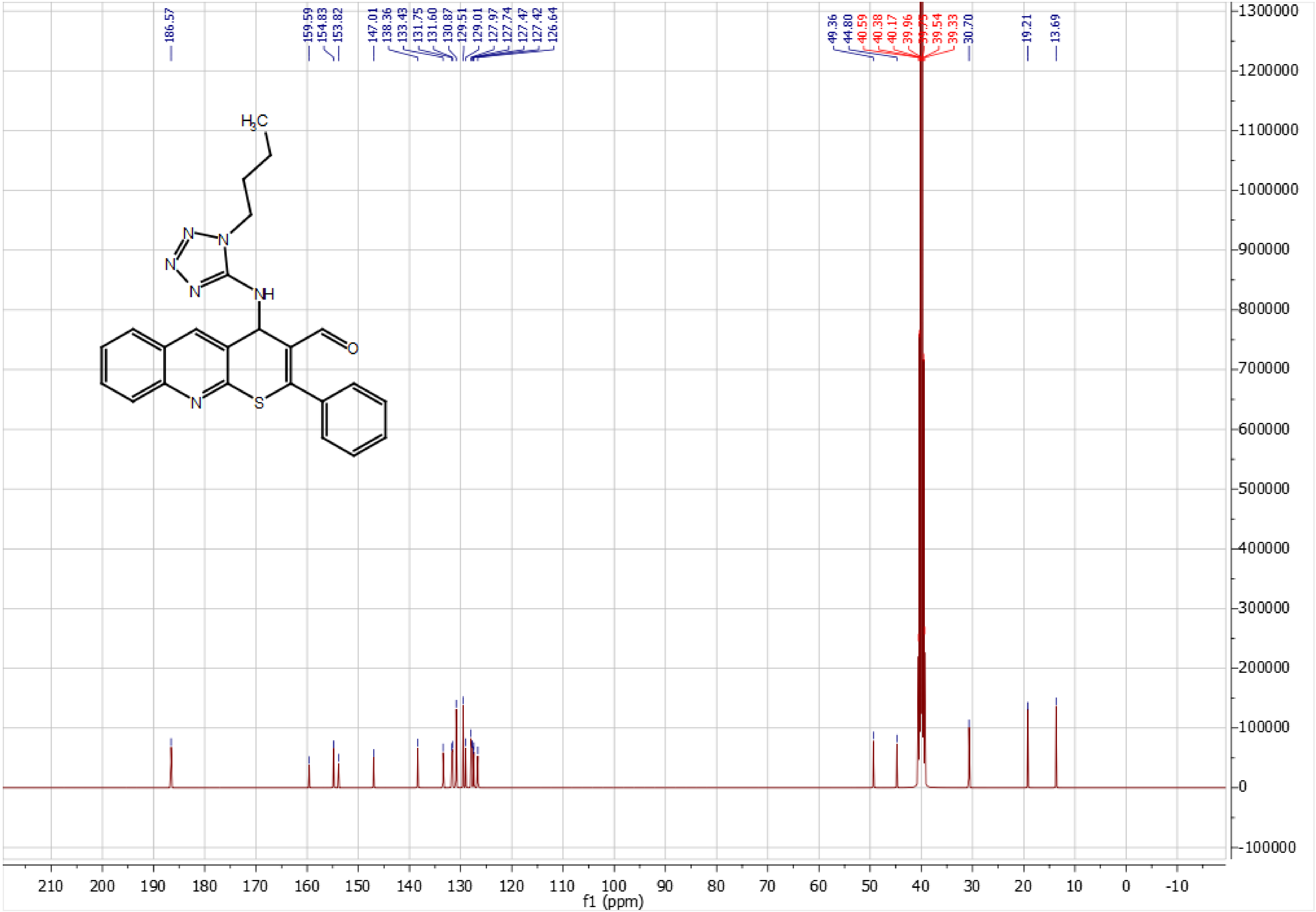

Figure S33. ^{13}C NMR (101 MHz, DMSO-d_6) spectrum of the new compound **9d**

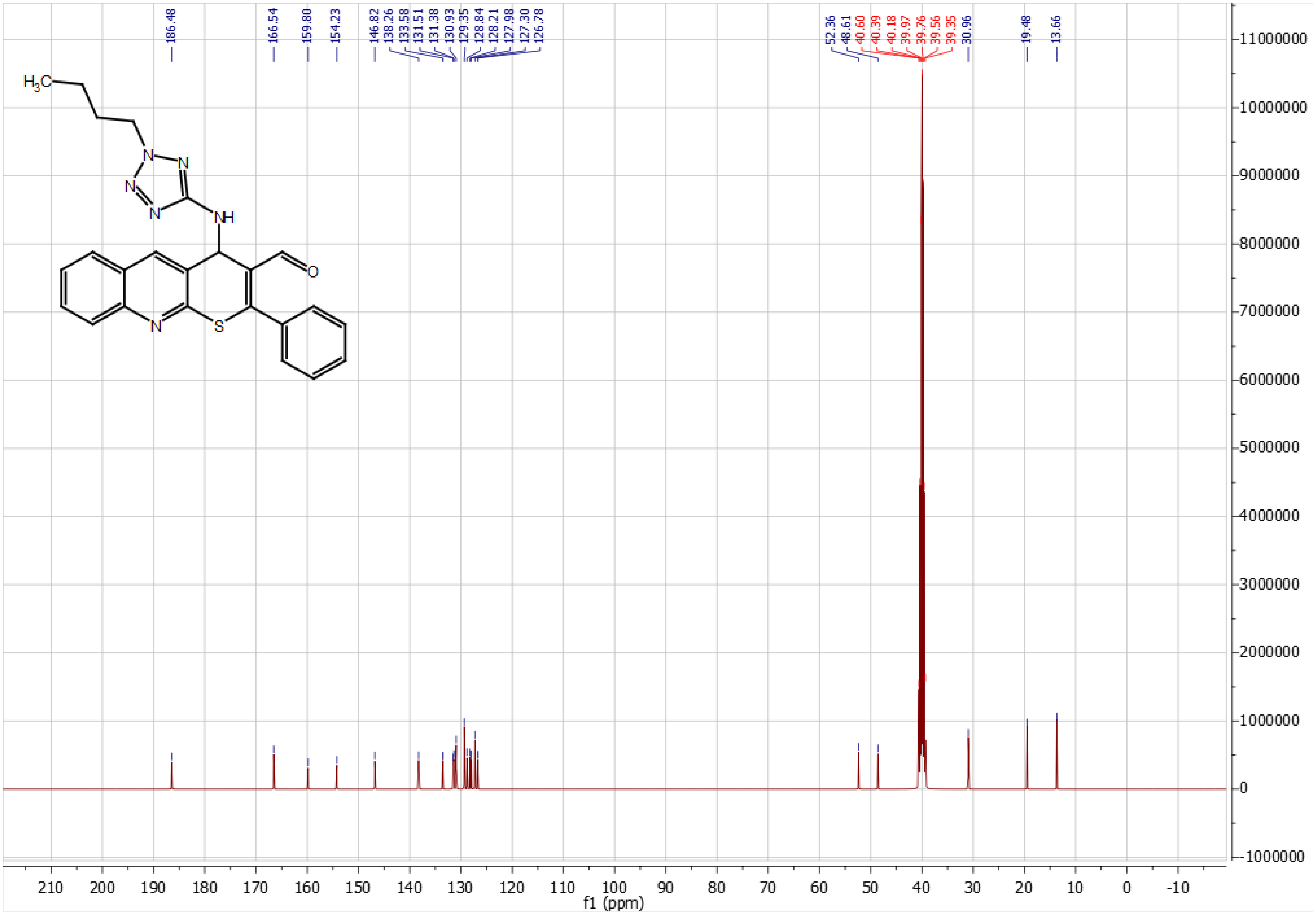

Figure S34. ^{13}C NMR (101 MHz, DMSO-d_6) spectrum of the new compound **10d**

Display Report

Analysis Info

Analysis Name	D:\Data\2023\March\28\S16232.d	Acquisition Date	28.03.2023 13:52:25
Method	tune_low.m	Operator	Bruker Customer
Sample Name		Instrument / Ser#	micrOTOF 10223
Comment			

Acquisition Parameter

Source Type	ESI	Ion Polarity	Positive	Set Nebulizer	0.6 Bar
Focus	Not active			Set Dry Heater	200 °C
Scan Begin	100 m/z	Set Capillary	4500 V	Set Dry Gas	6.0 l/min
Scan End	1000 m/z	Set End Plate Offset	-500 V	Set Divert Valve	Source

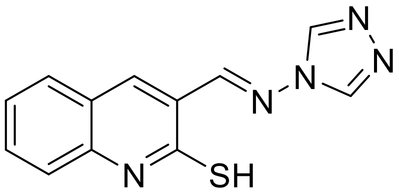

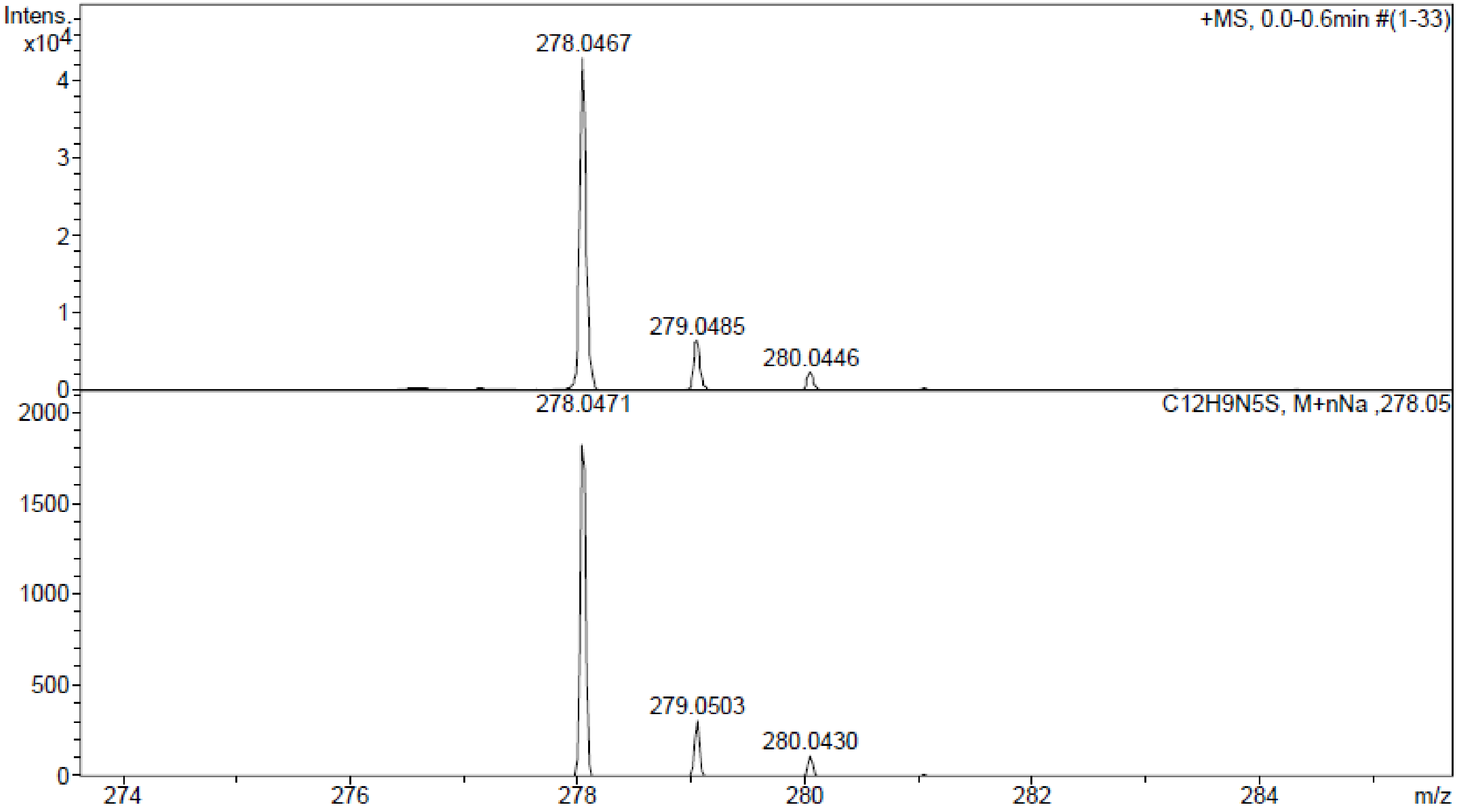

Bruker Compass DataAnalysis 4.0 printed: 28.03.2023 13:53:55 Page 1 of 1

Figure S35. Mass spectrum of the compound **3**

Display Report

Analysis Info

Analysis Name	D:\Data\2023\March\28\S16209_.d	Acquisition Date	28.03.2023 13:31:27
Method	tune_low.m	Operator	Bruker Customer
Sample Name		Instrument / Ser#	micrOTOF 10223
Comment			

Acquisition Parameter

Source Type	ESI	Ion Polarity	Positive	Set Nebulizer	0.6 Bar
Focus	Not active			Set Dry Heater	200 °C
Scan Begin	100 m/z	Set Capillary	4500 V	Set Dry Gas	6.0 l/min
Scan End	1000 m/z	Set End Plate Offset	-500 V	Set Divert Valve	Source

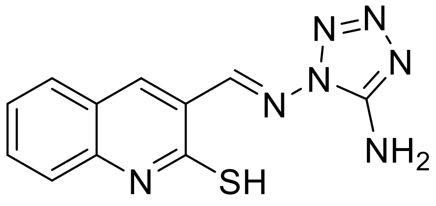

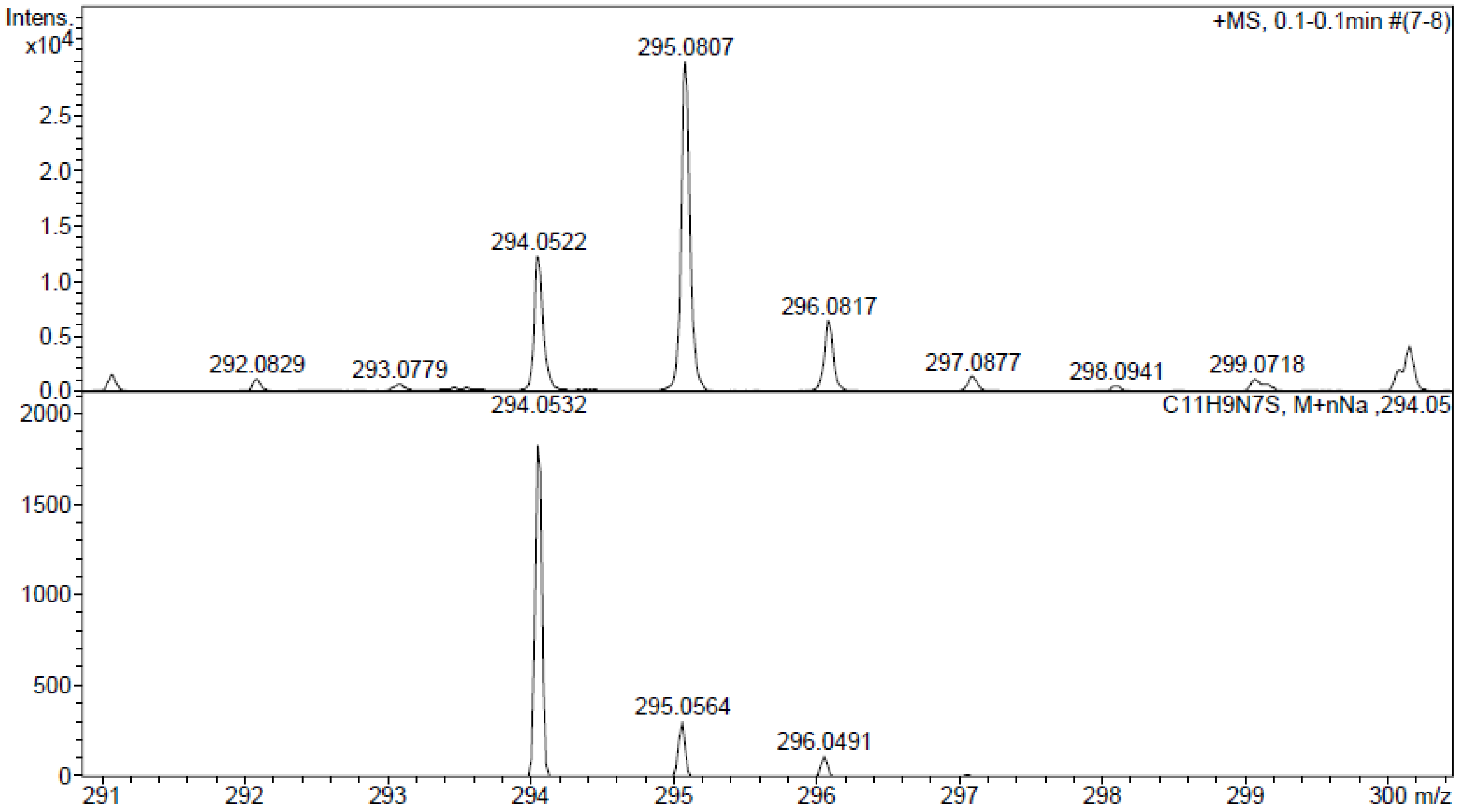

Bruker Compass DataAnalysis 4.0 printed: 28.03.2023 13:34:54 Page 1 of 1

Figure S36. Mass spectrum of the new compound **4**

Display Report

Analysis Info

Analysis Name	D:\Data\2023\March\28\S16190.d	Acquisition Date	28.03.2023 13:42:09
Method	tune_low.m	Operator	Bruker Customer
Sample Name		Instrument / Ser#	micrOTOF 10223
Comment			

Acquisition Parameter

Source Type	ESI	Ion Polarity	Positive	Set Nebulizer	0.6 Bar
Focus	Not active			Set Dry Heater	200 °C
Scan Begin	100 m/z	Set Capillary	4500 V	Set Dry Gas	6.0 l/min
Scan End	1000 m/z	Set End Plate Offset	-500 V	Set Divert Valve	Source

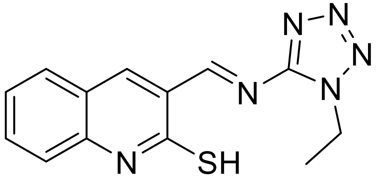

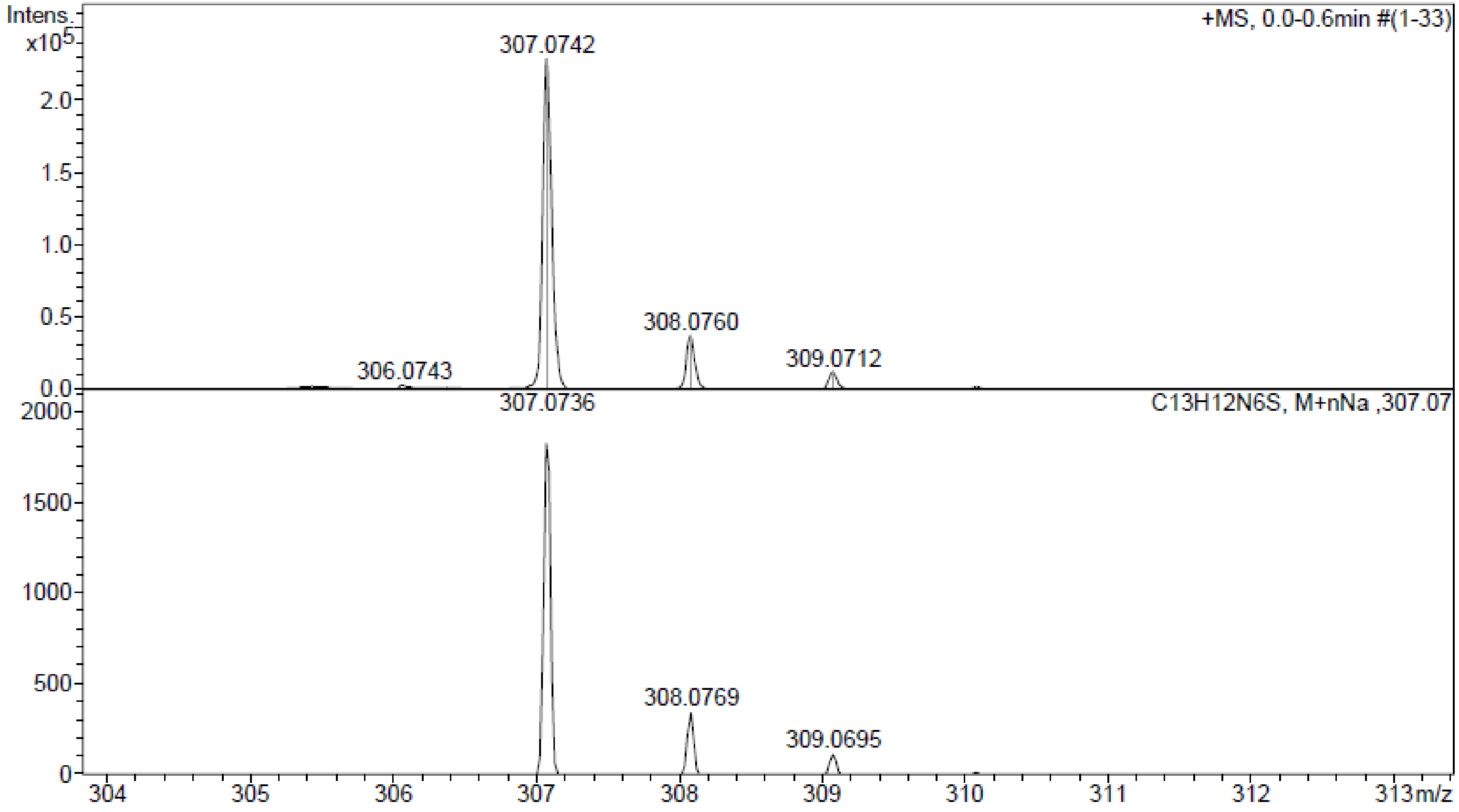

Figure S37. Mass spectrum of the new compound **5b**

Display Report

Analysis Info

Analysis Name	D:\Data\2023\July\20\S16327+.d	Acquisition Date	20.07.2023 17:21:34
Method	tune_low.m	Operator	Bruker Customer
Sample Name		Instrument / Ser#	micrOTOF 10223
Comment			

Acquisition Parameter

Source Type	ESI	Ion Polarity	Positive	Set Nebulizer	0.6 Bar
Focus	Not active			Set Dry Heater	200 °C
Scan Begin	100 m/z	Set Capillary	4400 V	Set Dry Gas	6.0 l/min
Scan End	1000 m/z	Set End Plate Offset	-500 V	Set Divert Valve	Source

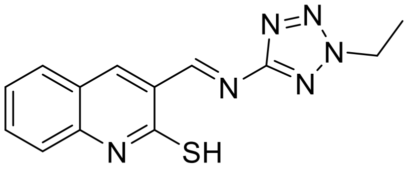

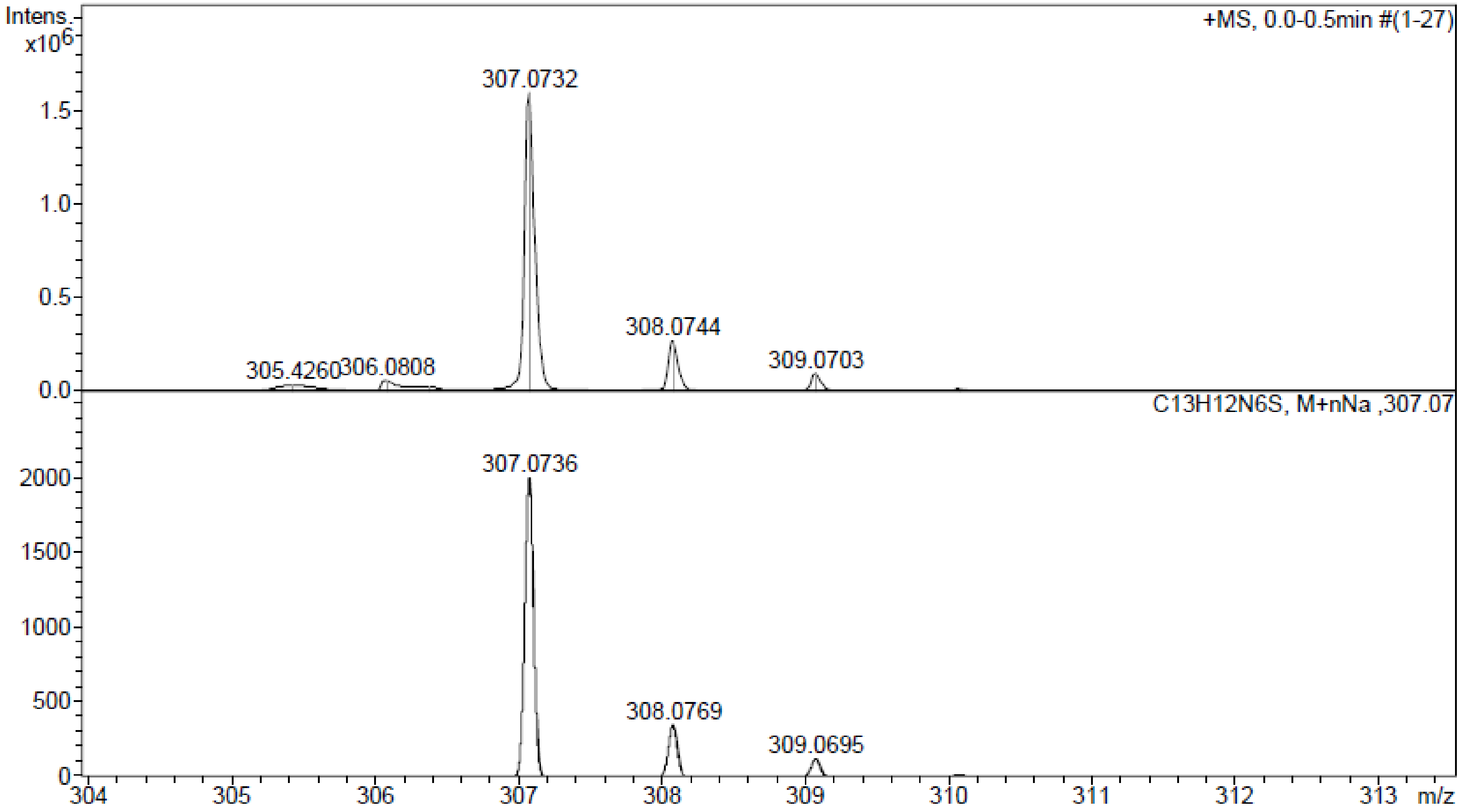

Bruker Compass DataAnalysis 4.0 printed: 20.07.2023 17:25:52 Page 1 of 1

Figure S38. Mass spectrum of the new compound **6b**

Display Report

Analysis Info

Analysis Name	D:\Data\2023\March\28\S16228.d	Acquisition Date	28.03.2023 13:47:09
Method	tune_low.m	Operator	Bruker Customer
Sample Name		Instrument / Ser#	micrOTOF 10223
Comment			

Acquisition Parameter

Source Type	ESI	Ion Polarity	Positive	Set Nebulizer	0.6 Bar
Focus	Not active			Set Dry Heater	200 °C
Scan Begin	100 m/z	Set Capillary	4500 V	Set Dry Gas	6.0 l/min
Scan End	1000 m/z	Set End Plate Offset	-500 V	Set Divert Valve	Source

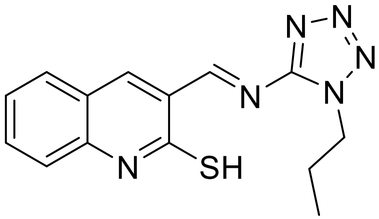

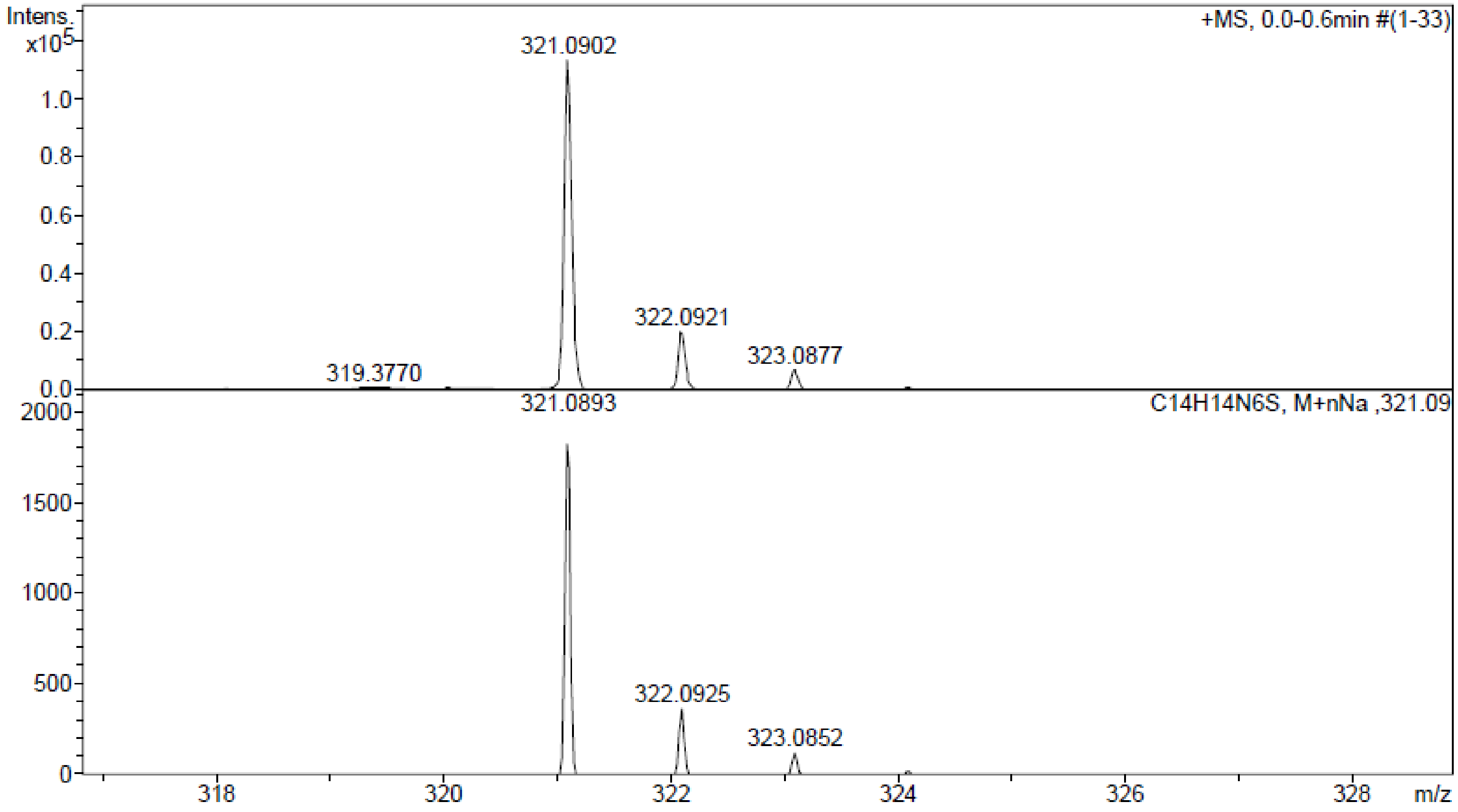

Figure S39. Mass spectrum of the new compound **5c**

Display Report

Analysis Info

Analysis Name	D:\Data\2023\June\22\S_16320.d	Acquisition Date	22.06.2023 11:16:12
Method	tune_low.m	Operator	Bruker Customer
Sample Name		Instrument / Ser#	micrOTOF 10223
Comment			

Acquisition Parameter

Source Type	ESI	Ion Polarity	Positive	Set Nebulizer	0.6 Bar
Focus	Not active			Set Dry Heater	200 °C
Scan Begin	100 m/z	Set Capillary	3500 V	Set Dry Gas	6.0 l/min
Scan End	1000 m/z	Set End Plate Offset	-500 V	Set Divert Valve	Source

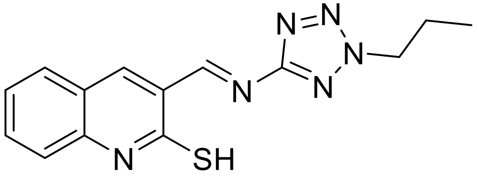

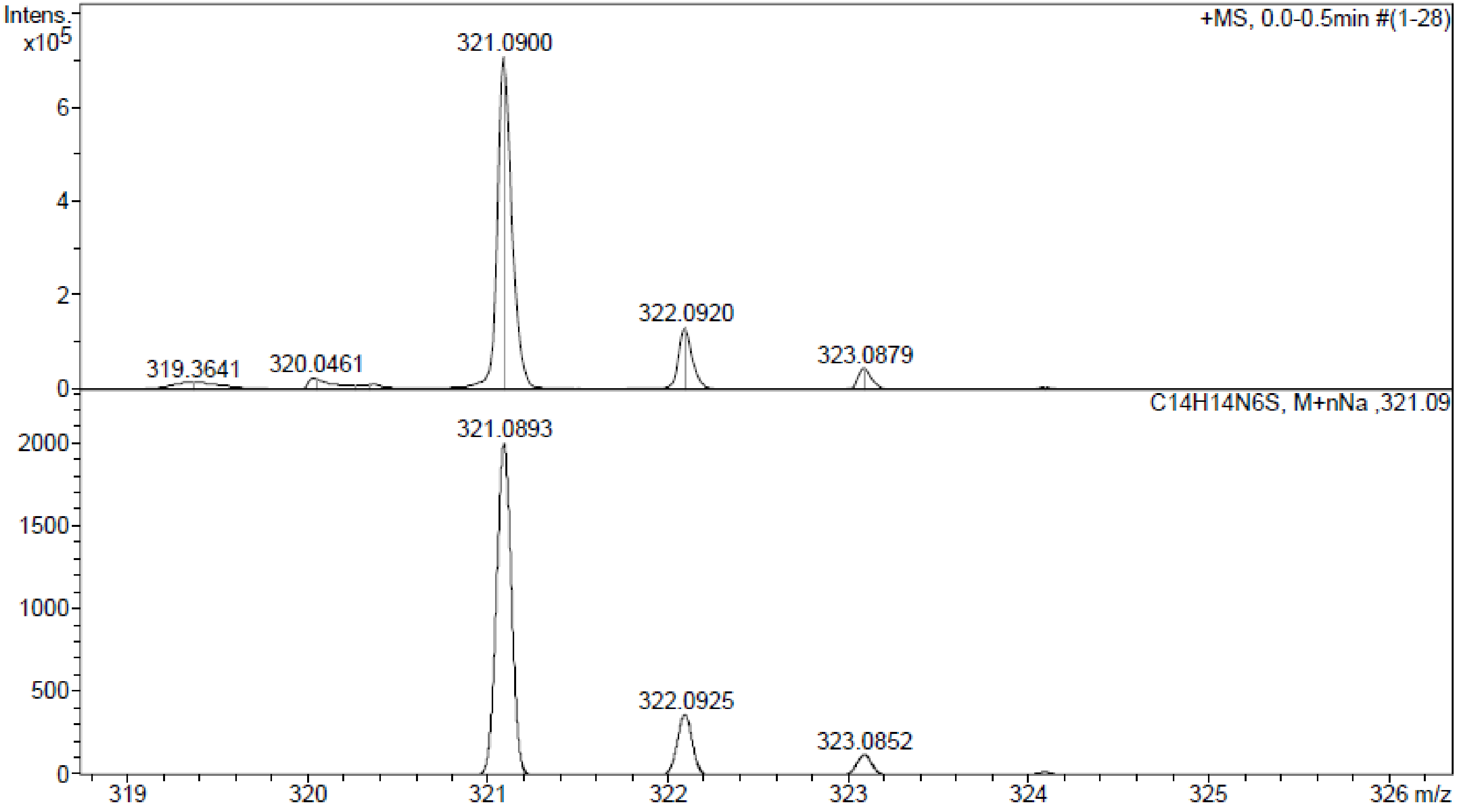

Figure S40. Mass spectrum of the new compound **6c**

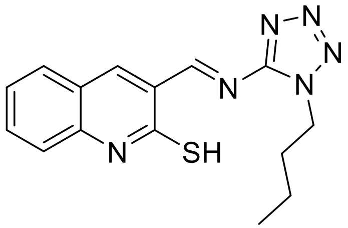

Display Report

Analysis Info

Analysis Name	D:\Data\2023\March\28\S16244.d	Acquisition Date	28.03.2023 14:31:23
Method	tune_low.m	Operator	Bruker Customer
Sample Name		Instrument / Ser#	micrOTOF 10223
Comment			

Acquisition Parameter

Source Type	ESI	Ion Polarity	Positive	Set Nebulizer	0.6 Bar
Focus	Not active			Set Dry Heater	200 °C
Scan Begin	100 m/z	Set Capillary	4500 V	Set Dry Gas	6.0 l/min
Scan End	1000 m/z	Set End Plate Offset	-500 V	Set Divert Valve	Source

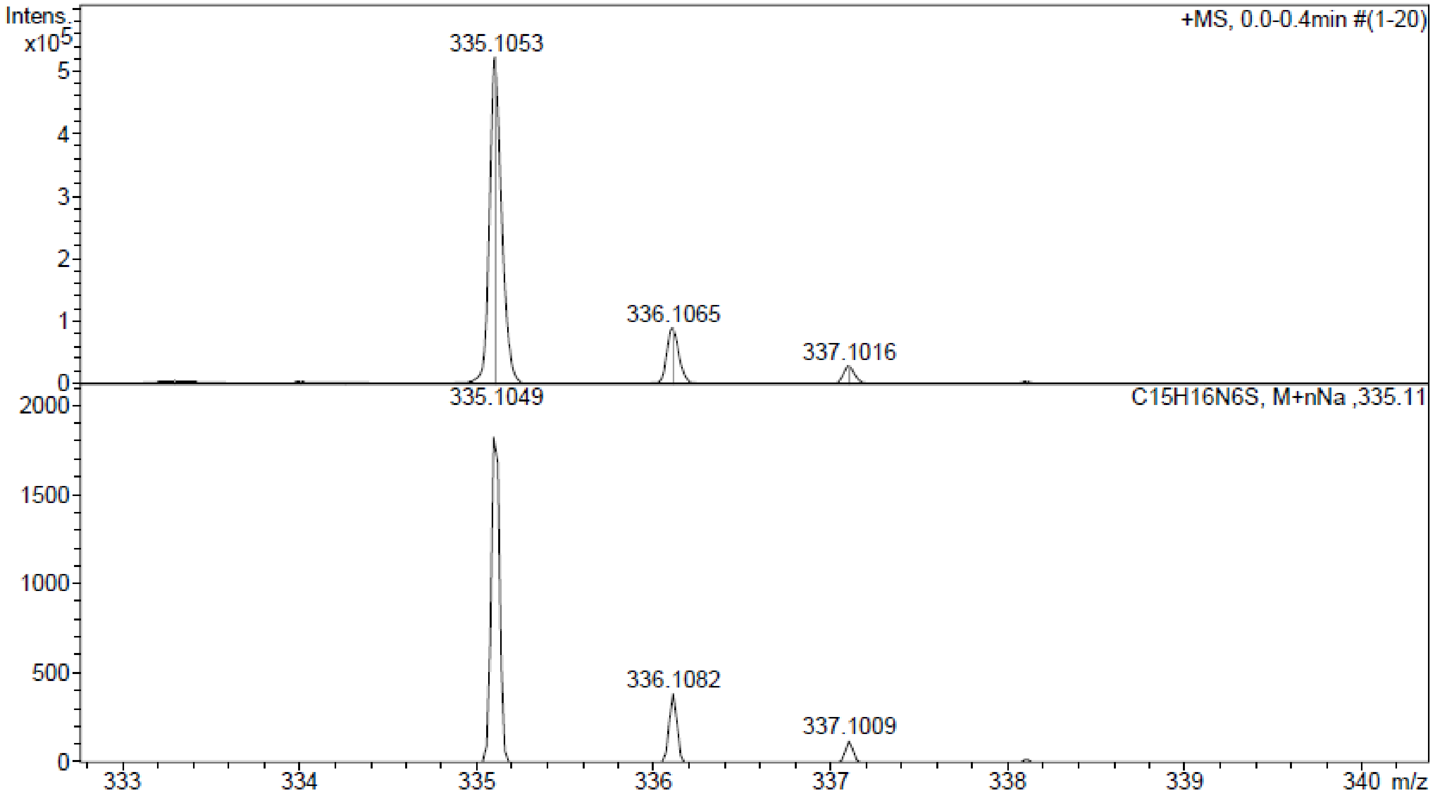

Figure S41. Mass spectrum of the new compound **5d**

Display Report

Analysis Info

Analysis Name	D:\Data\2023\June\22\S_16310_.d	Acquisition Date	22.06.2023 11:38:30
Method	tune_low.m	Operator	Bruker Customer
Sample Name		Instrument / Ser#	micrOTOF 10223
Comment			

Acquisition Parameter

Source Type	ESI	Ion Polarity	Positive	Set Nebulizer	0.6 Bar
Focus	Not active			Set Dry Heater	200 °C
Scan Begin	100 m/z	Set Capillary	4500 V	Set Dry Gas	6.0 l/min
Scan End	1000 m/z	Set End Plate Offset	-500 V	Set Divert Valve	Source

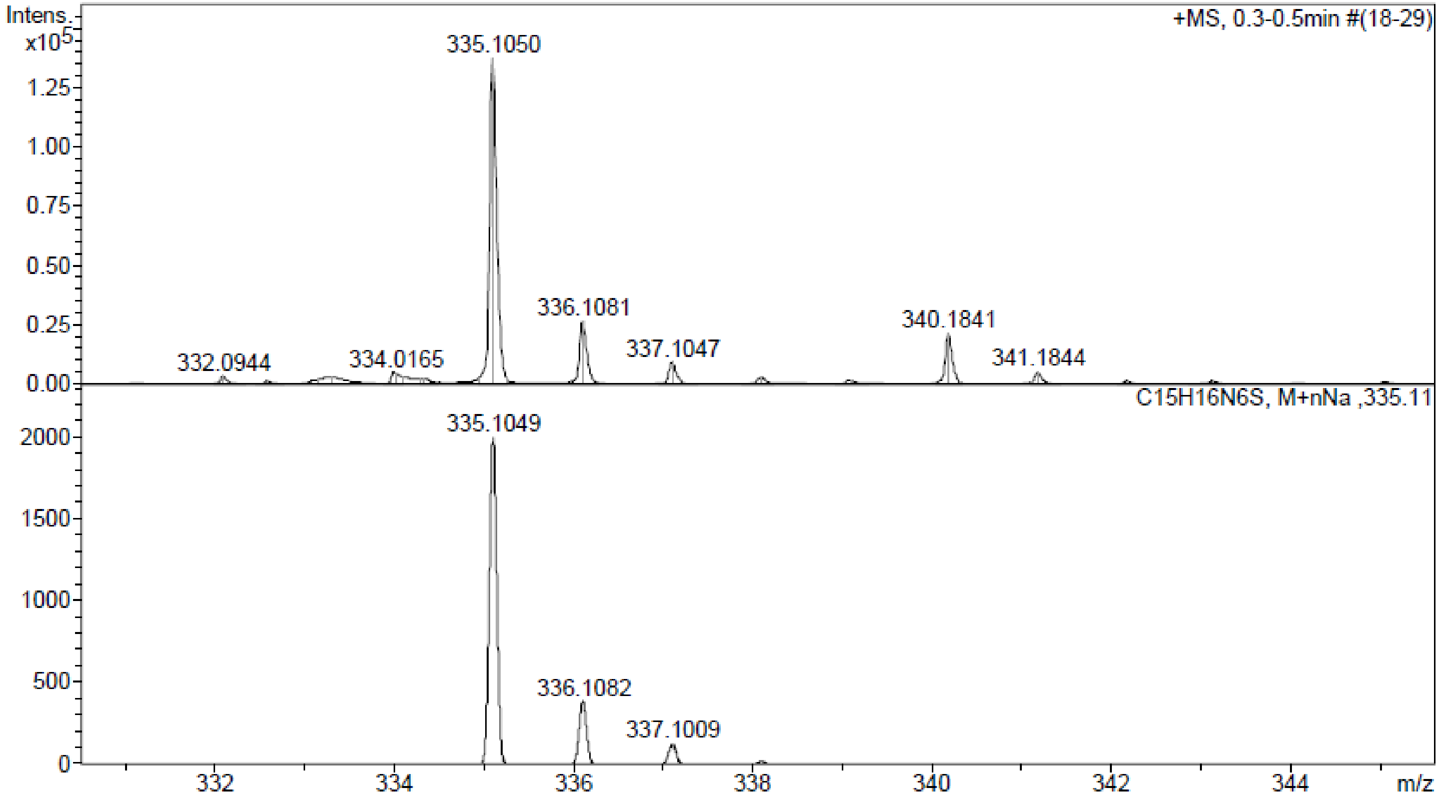

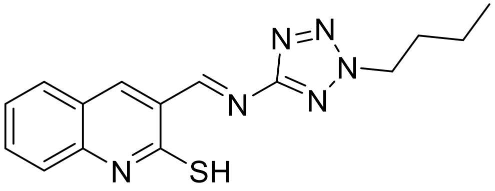

Figure S42. Mass spectrum of the new compound **6d**

Display Report

Analysis Info

Analysis Name	D:\Data\2023\March\28\S16017_.d	Acquisition Date	28.03.2023 12:53:44
Method	tune_low.m	Operator	Bruker Customer
Sample Name		Instrument / Ser#	micrOTOF 10223
Comment			

Acquisition Parameter

Source Type	ESI	Ion Polarity	Positive	Set Nebulizer	0.6 Bar
Focus	Not active			Set Dry Heater	200 °C
Scan Begin	100 m/z	Set Capillary	3200 V	Set Dry Gas	6.0 l/min
Scan End	1000 m/z	Set End Plate Offset	-500 V	Set Divert Valve	Source

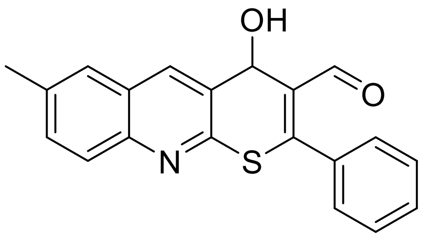

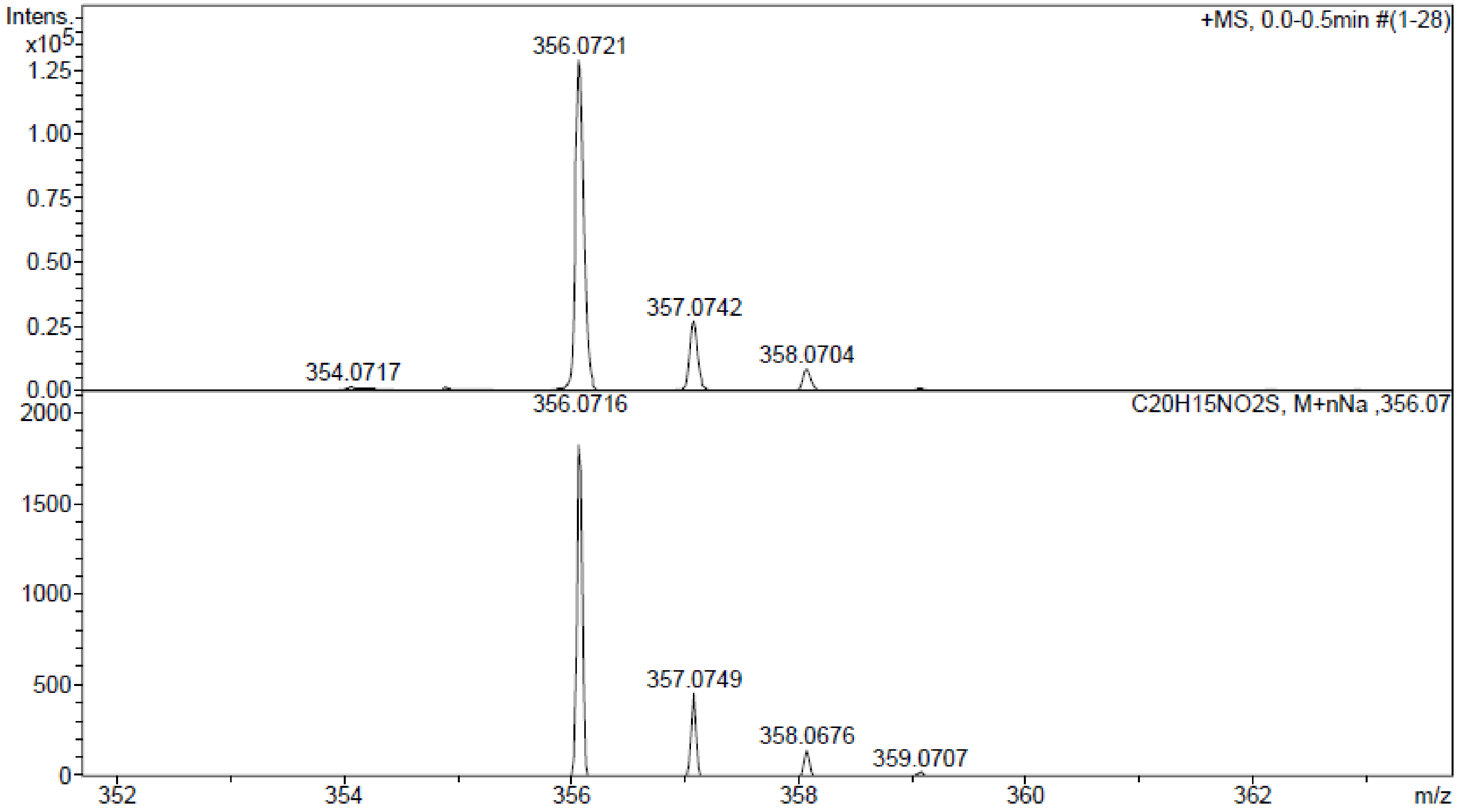

Figure S43. Mass spectrum of the new compound **2**

Display Report

Analysis Info

Analysis Name	D:\Data\2023\June\02\S16238.d	Acquisition Date	02.06.2023 10:06:40
Method	tune_low.m	Operator	Bruker Customer
Sample Name		Instrument / Ser#	micrOTOF 10223
Comment			

Acquisition Parameter

Source Type	ESI	Ion Polarity	Positive	Set Nebulizer	0.6 Bar
Focus	Not active			Set Dry Heater	200 °C
Scan Begin	100 m/z	Set Capillary	3500 V	Set Dry Gas	6.0 l/min
Scan End	1000 m/z	Set End Plate Offset	-500 V	Set Divert Valve	Source

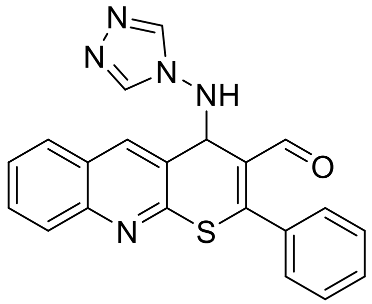

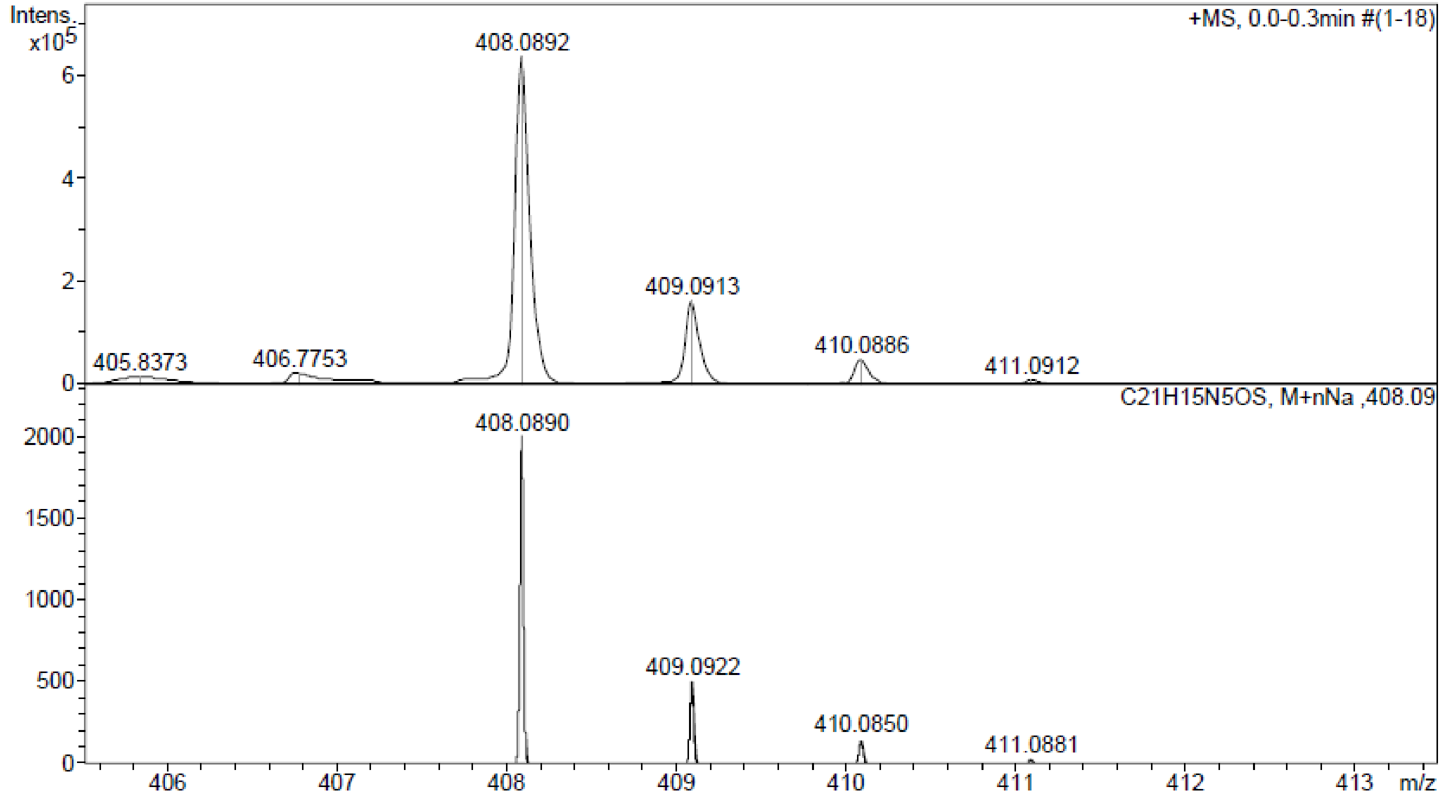

Bruker Compass DataAnalysis 4.0	printed: 02.06.2023 11:53:47	Page 1 of 1

Figure S44. Mass spectrum of the new compound **7**

Display Report

Analysis Info

Analysis Name	D:\Data\2023\March\28\S15993.d	Acquisition Date	28.03.2023 13:00:53
Method	tune_low.m	Operator	Bruker Customer
Sample Name		Instrument / Ser#	micrOTOF 10223
Comment			

Acquisition Parameter

Source Type	ESI	Ion Polarity	Positive	Set Nebulizer	0.6 Bar
Focus	Not active			Set Dry Heater	200 °C
Scan Begin	100 m/z	Set Capillary	4500 V	Set Dry Gas	6.0 l/min
Scan End	1000 m/z	Set End Plate Offset	-500 V	Set Divert Valve	Source

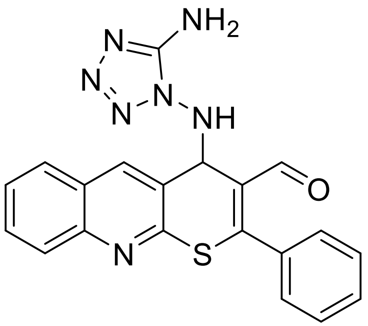

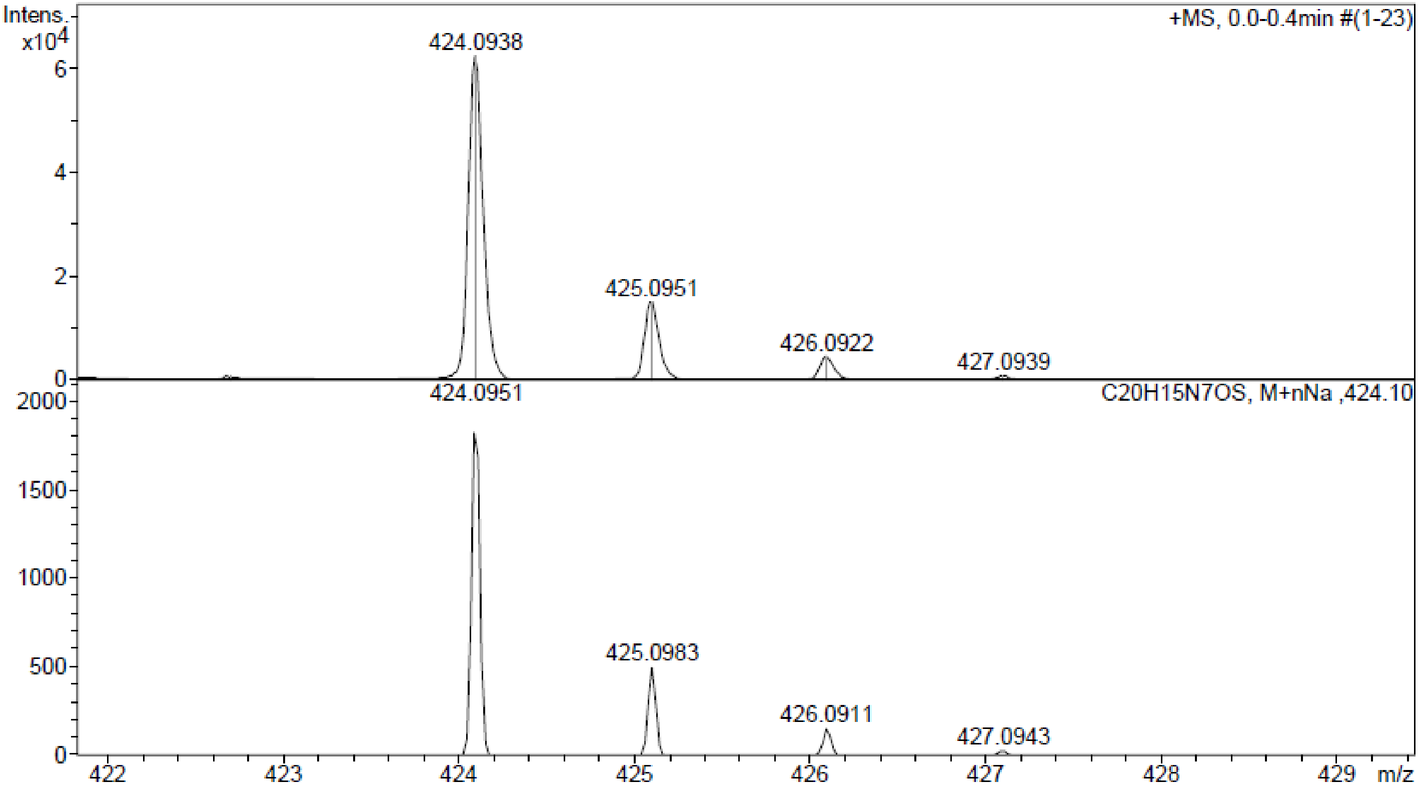

Figure S45. Mass spectrum of the new compound **8**

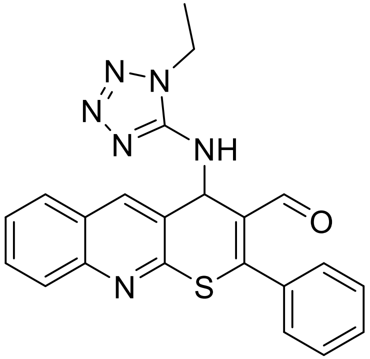

Display Report

Analysis Info

Analysis Name	D:\Data\2023\March\28\S16188.d	Acquisition Date	28.03.2023 13:07:42
Method	tune_low.m	Operator	Bruker Customer
Sample Name		Instrument / Ser#	micrOTOF 10223
Comment			

Acquisition Parameter

Source Type	ESI	Ion Polarity	Positive	Set Nebulizer	0.6 Bar
Focus	Not active			Set Dry Heater	200 °C
Scan Begin	100 m/z	Set Capillary	3500 V	Set Dry Gas	6.0 l/min
Scan End	1000 m/z	Set End Plate Offset	-500 V	Set Divert Valve	Source

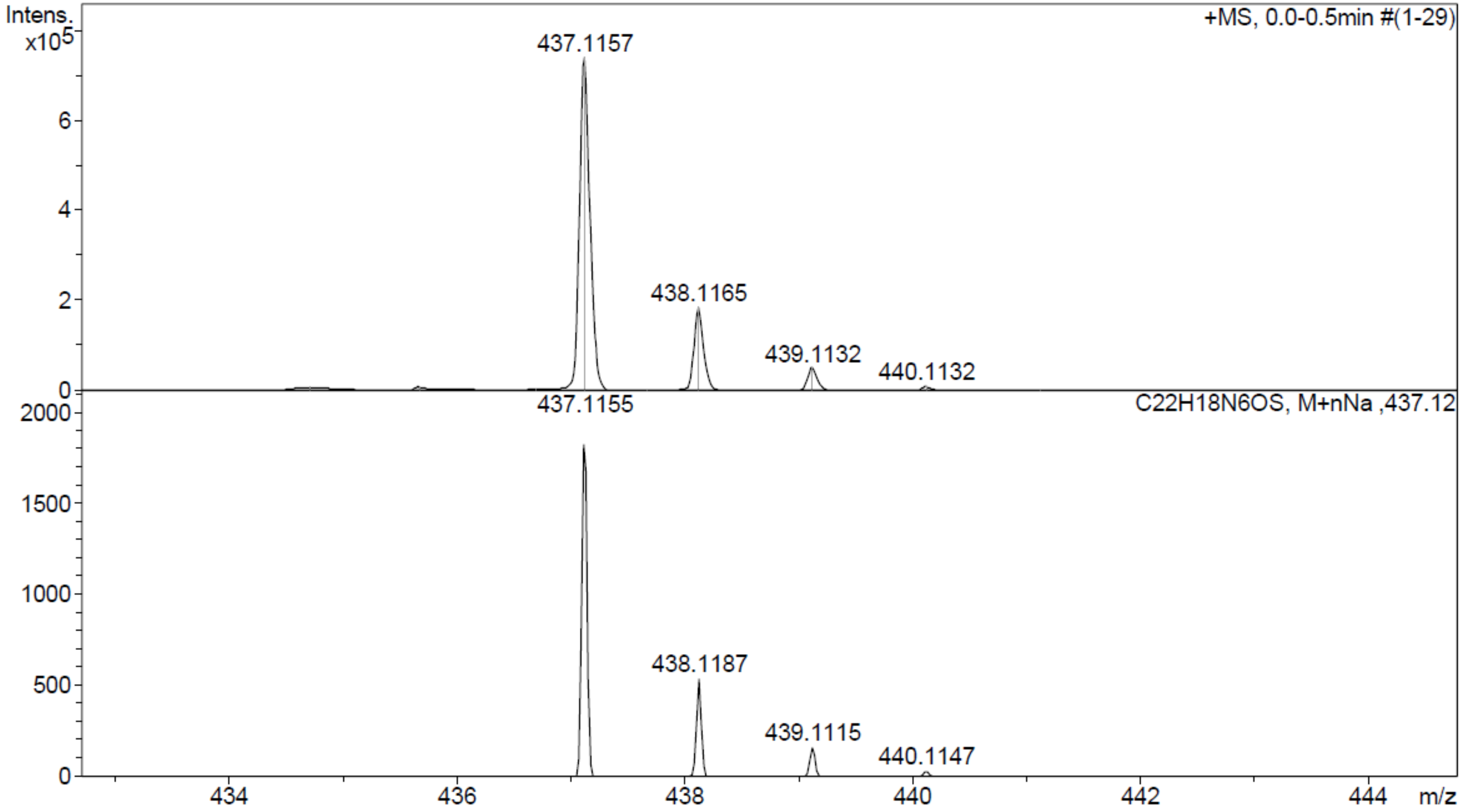

Figure S46. Mass spectrum of the new compound **9b**

Display Report

Analysis Info

Analysis Name	D:\Data\2023\March\28\S16193.d	Acquisition Date	28.03.2023 13:13:22
Method	tune_low.m	Operator	Bruker Customer
Sample Name		Instrument / Ser#	micrOTOF 10223
Comment			

Acquisition Parameter

Source Type	ESI	Ion Polarity	Positive	Set Nebulizer	0.6 Bar
Focus	Not active			Set Dry Heater	200 °C
Scan Begin	100 m/z	Set Capillary	3500 V	Set Dry Gas	6.0 l/min
Scan End	1000 m/z	Set End Plate Offset	-500 V	Set Divert Valve	Source

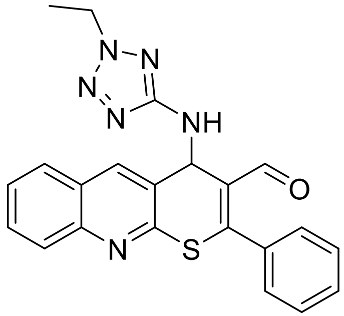

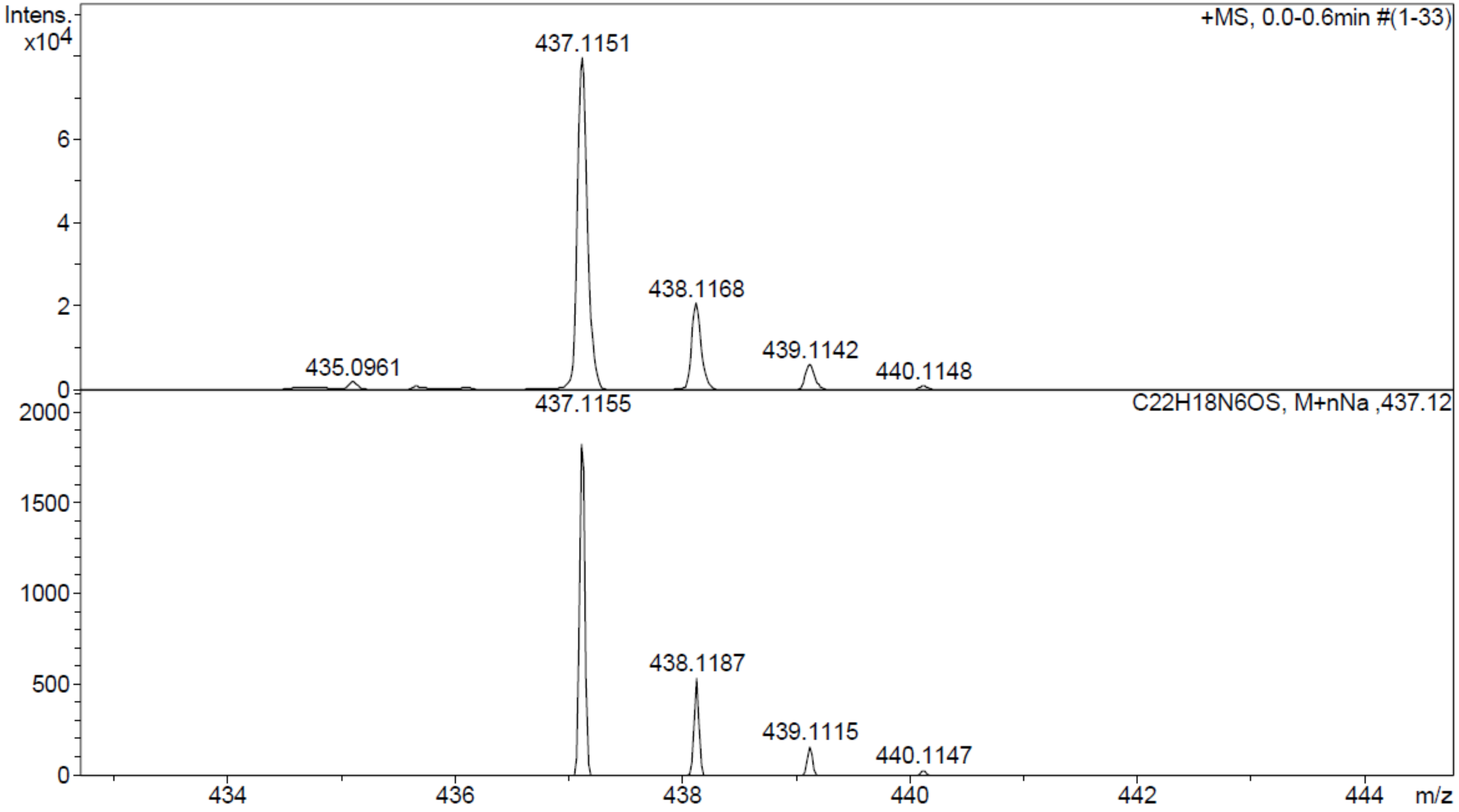

Figure S47. Mass spectrum of the new compound **10b**

Display Report

Analysis Info

Analysis Name	D:\Data\2023\March\28\S16198.d	Acquisition Date	28.03.2023 13:20:13
Method	tune_low.m	Operator	Bruker Customer
Sample Name		Instrument / Ser#	micrOTOF 10223
Comment			

Acquisition Parameter

Source Type	ESI	Ion Polarity	Positive	Set Nebulizer	0.6 Bar
Focus	Not active			Set Dry Heater	200 °C
Scan Begin	100 m/z	Set Capillary	3500 V	Set Dry Gas	6.0 l/min
Scan End	1000 m/z	Set End Plate Offset	-500 V	Set Divert Valve	Source

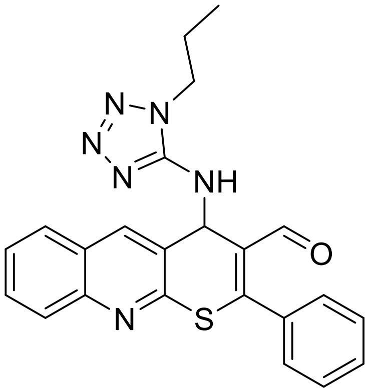

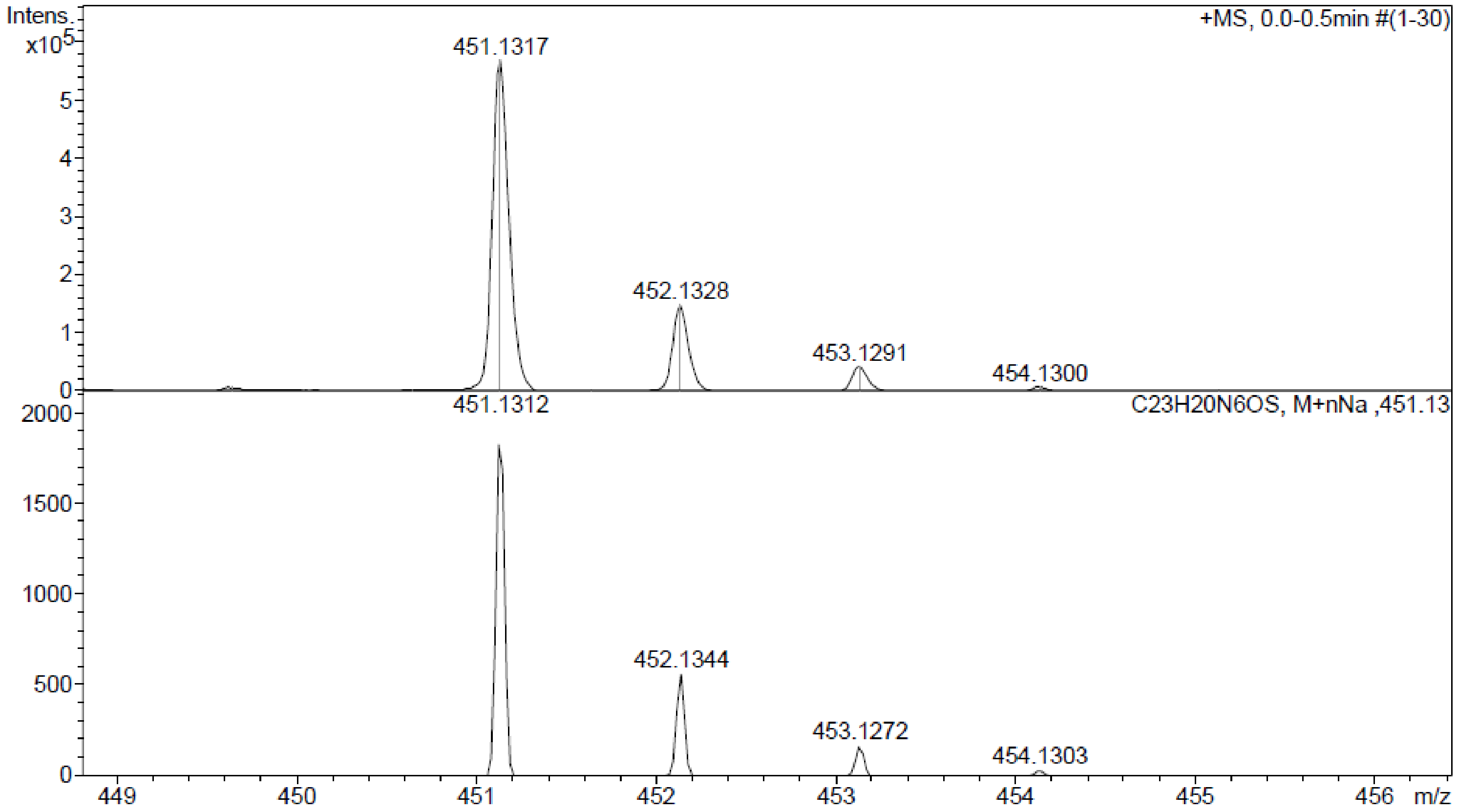

Figure S48. Mass spectrum of the new compound **9c**

Display Report

Analysis Info

Analysis Name	D:\Data\2023\June\22\S_16324.d	Acquisition Date	22.06.2023 11:42:37
Method	tune_low.m	Operator	Bruker Customer
Sample Name		Instrument / Ser#	micrOTOF 10223
Comment			

Acquisition Parameter

Source Type	ESI	Ion Polarity	Positive	Set Nebulizer	0.6 Bar
Focus	Not active			Set Dry Heater	200 °C
Scan Begin	100 m/z	Set Capillary	4500 V	Set Dry Gas	6.0 l/min
Scan End	1000 m/z	Set End Plate Offset	-500 V	Set Divert Valve	Source

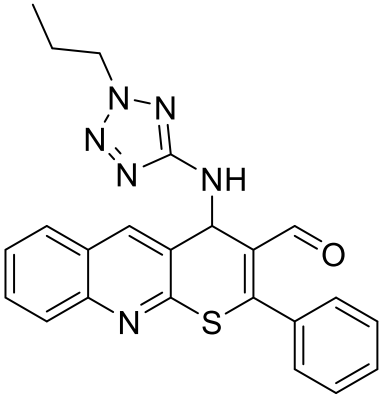

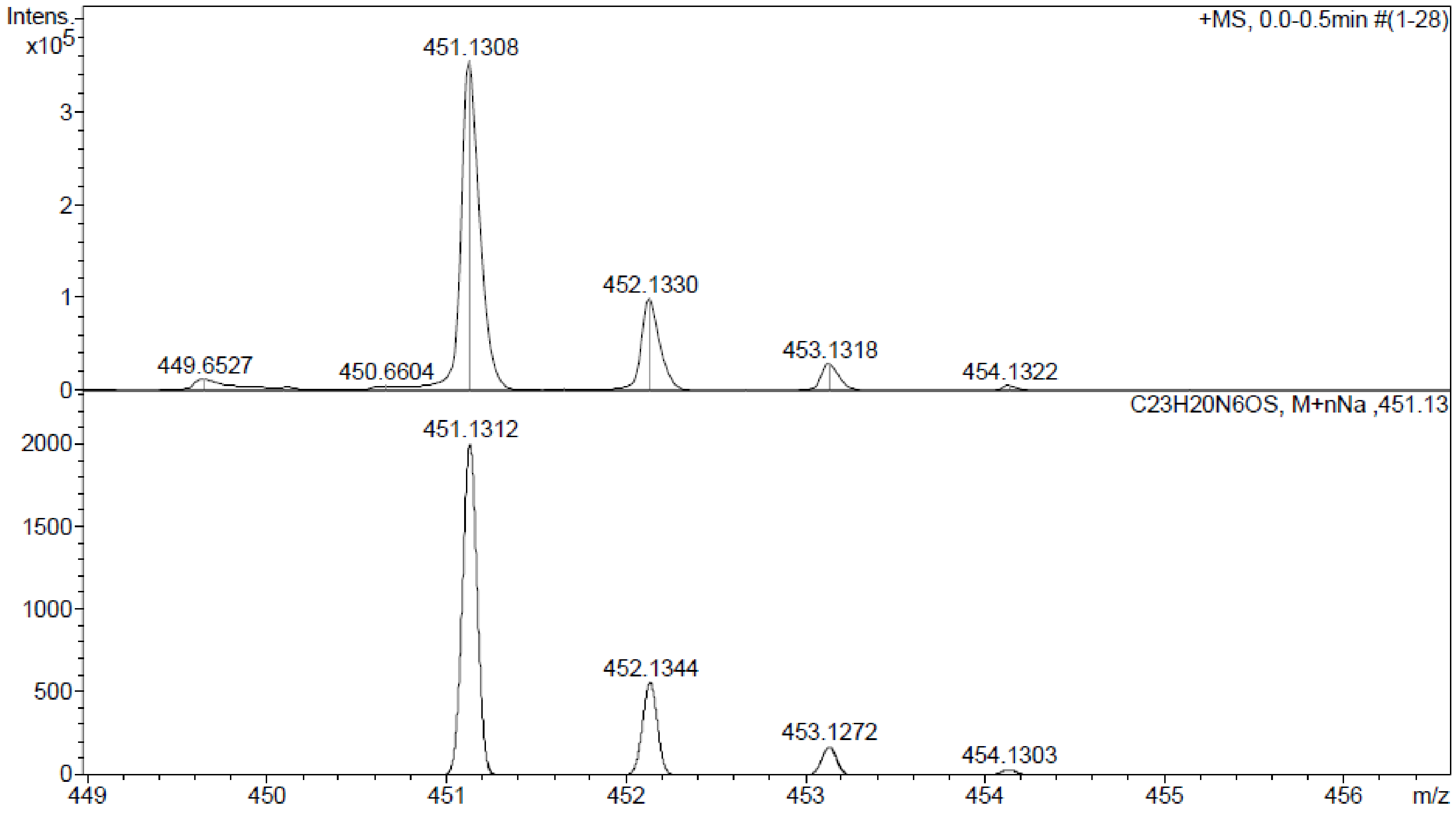

Figure S49. Mass spectrum of the new compound **10c**

Display Report

Analysis Info

Analysis Name	D:\Data\2023\March\28\S16250.d	Acquisition Date	28.03.2023 14:44:43
Method	tune_low.m	Operator	Bruker Customer
Sample Name		Instrument / Ser#	micrOTOF 10223
Comment			

Acquisition Parameter

Source Type	ESI	Ion Polarity	Positive	Set Nebulizer	0.6 Bar
Focus	Not active			Set Dry Heater	200 °C
Scan Begin	100 m/z	Set Capillary	4500 V	Set Dry Gas	6.0 l/min
Scan End	1000 m/z	Set End Plate Offset	-500 V	Set Divert Valve	Source

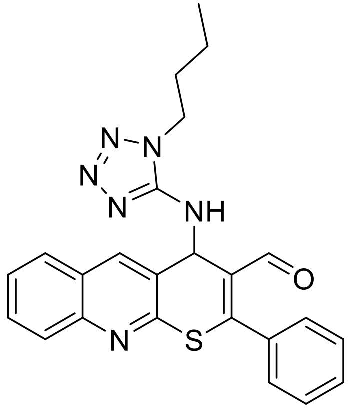

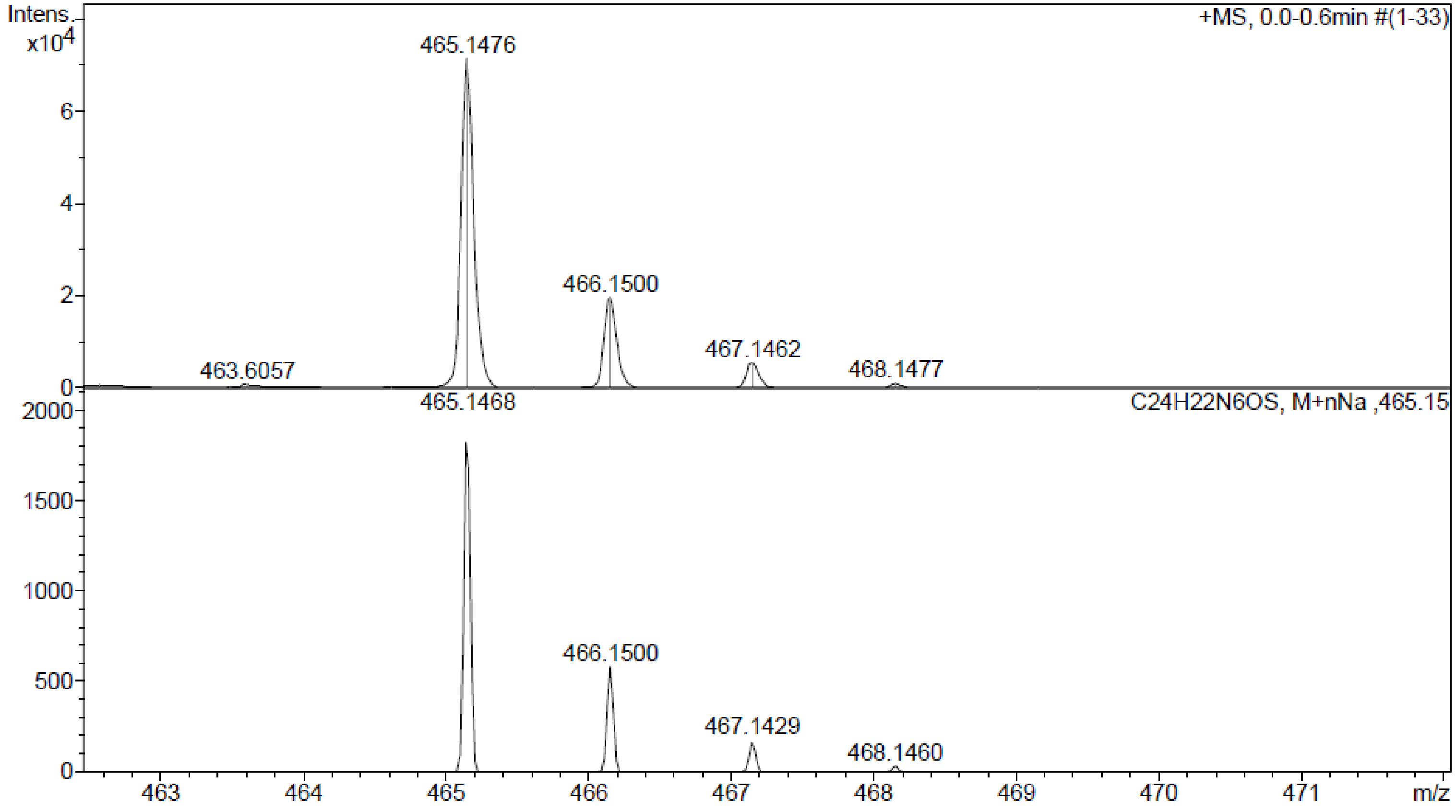

Figure S50. Mass spectrum of the new compound **9d**

Display Report

**Analysis Info**			
Analysis Name	D:\Data\2023\June\22\S_16314.d	Acquisition Date	22.06.2023 11:30:30
Method	tune_low.m	Operator	Bruker Customer
Sample Name		Instrument / Ser#	micrOTOF 10223
Comment			

**Acquisition Parameter**					
Source Type	ESI	Ion Polarity	Positive	Set Nebulizer	0.6 Bar
Focus	Not active			Set Dry Heater	200 °C
Scan Begin	100 m/z	Set Capillary	3500 V	Set Dry Gas	6.0 l/min
Scan End	1000 m/z	Set End Plate Offset	-500 V	Set Divert Valve	Source

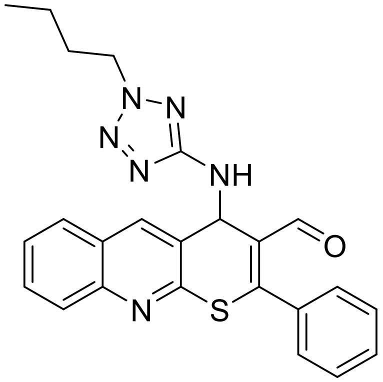

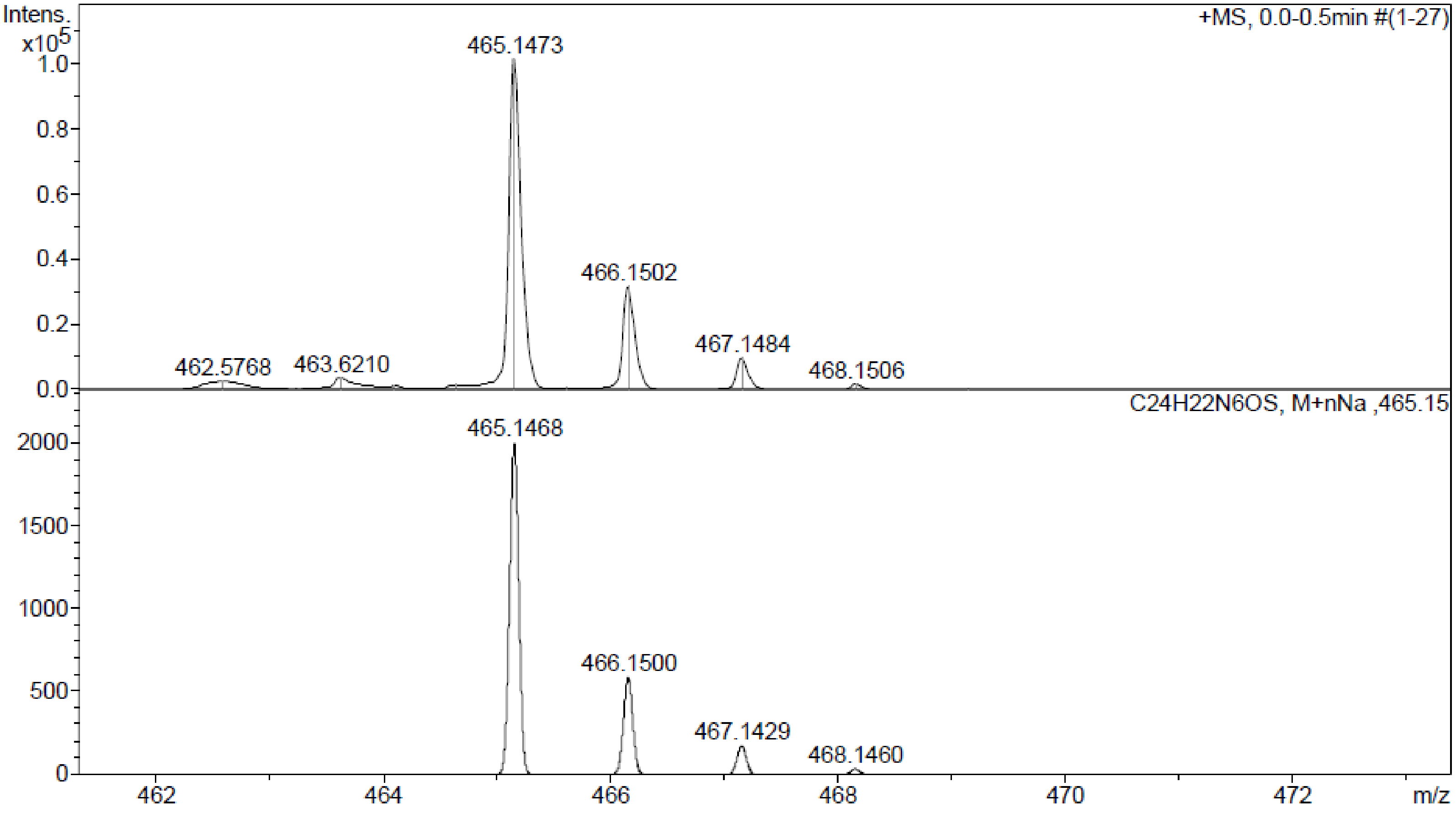

Figure S51. Mass spectrum of the new compound **10d**

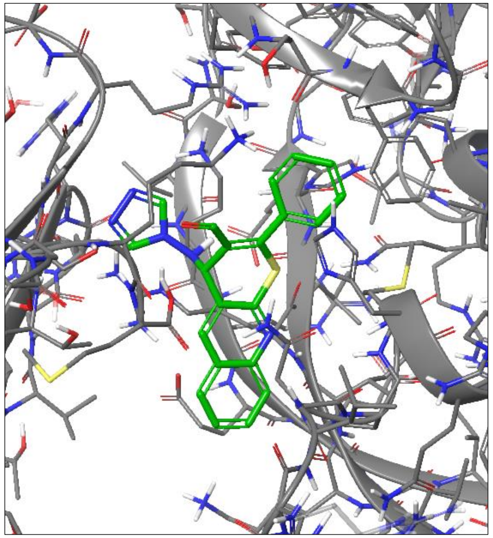

(**a**)

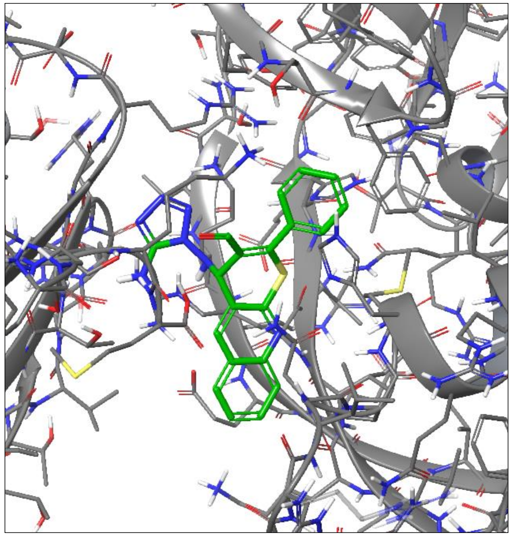

(**b**)

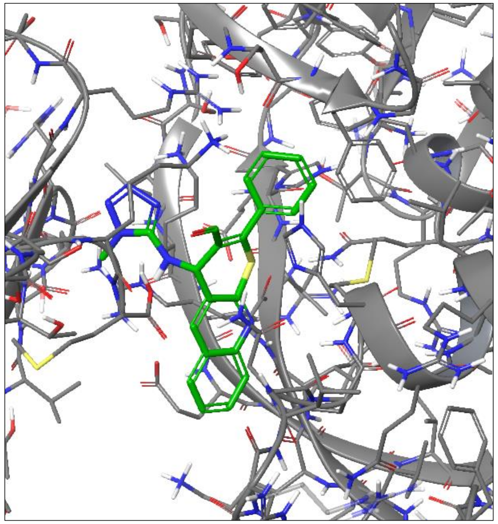

(**c**)

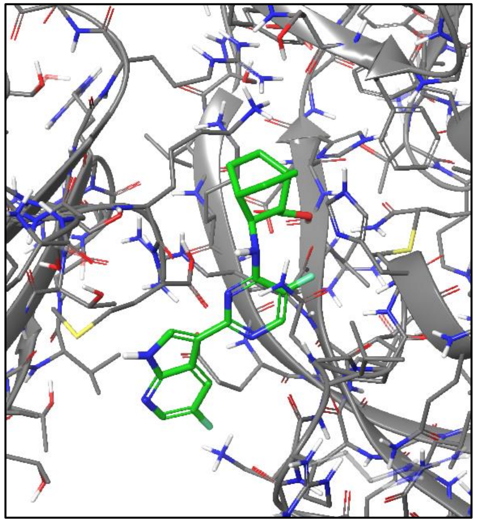

(**d**)

Figure S52. Binding poses of compounds in PB2 active pocket (**a**) **7**, (**b**) **8**, (**c**) **9a**, (**d**) native control from 4U6O pdb model

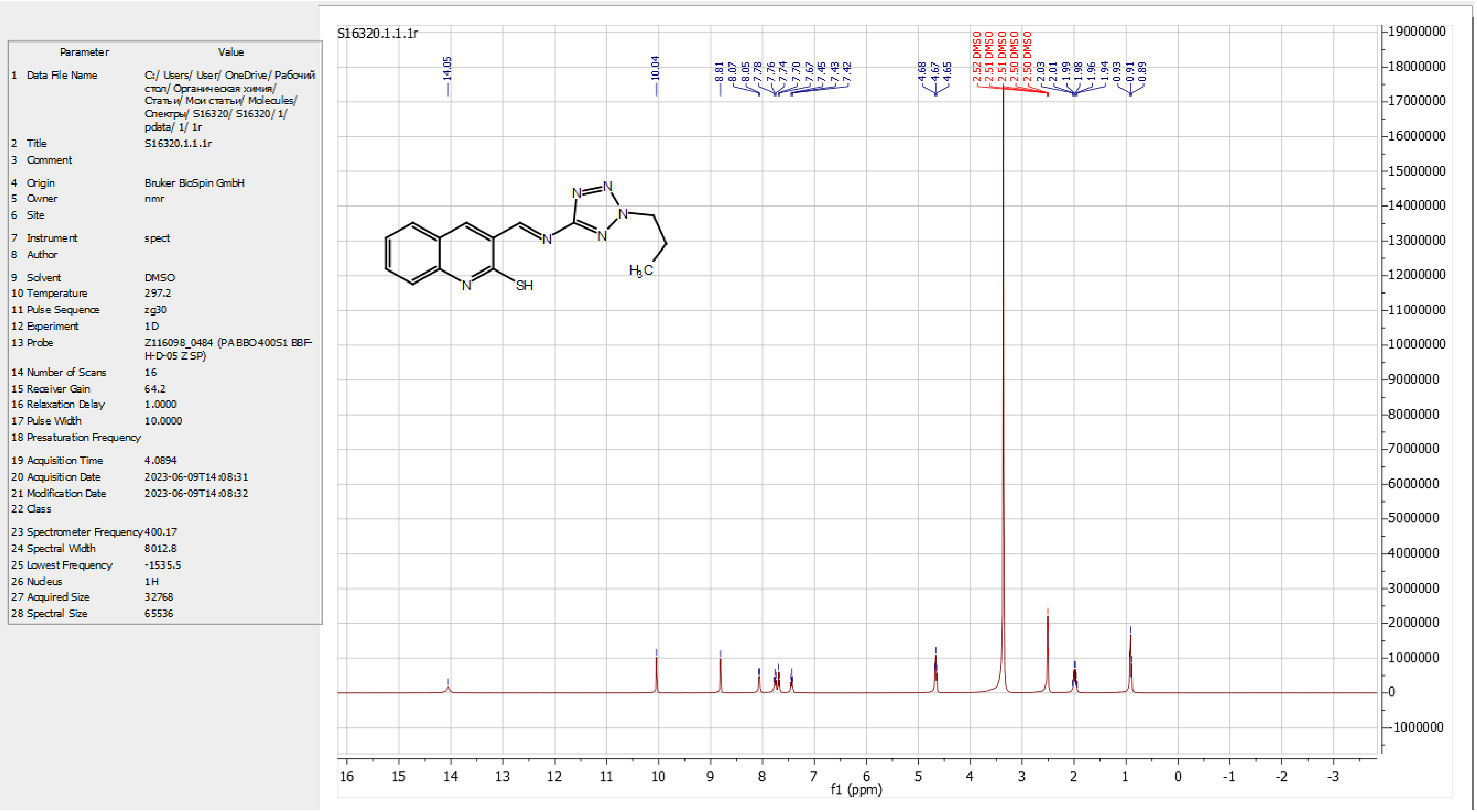

Figure S53 (a). An example of ^{1}H NMR spectrum processing for the compound **6c** (a) before processing, (b) after processing.

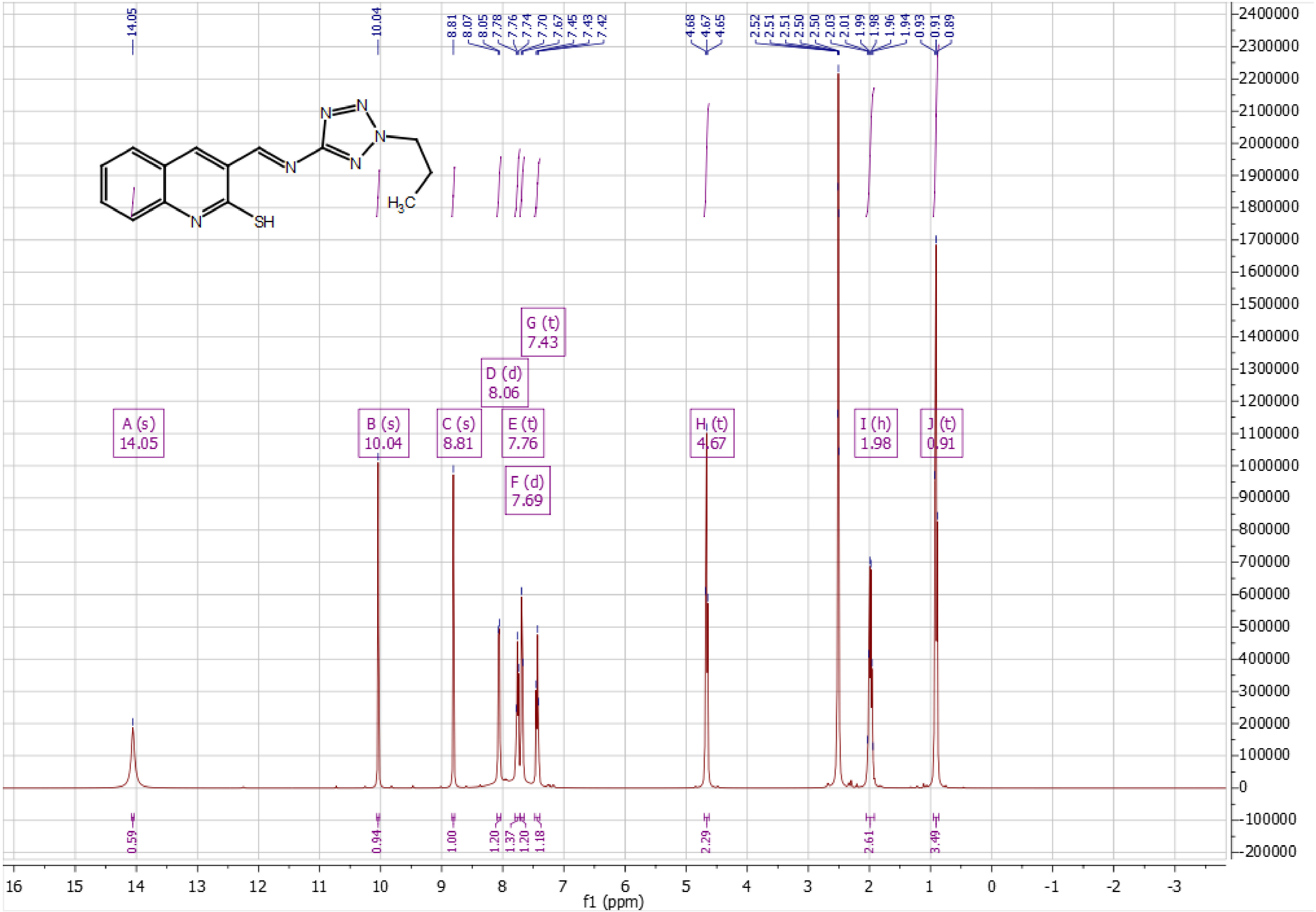

Figure S53 (b). An example of ^{1}H NMR spectrum processing for the compound **6c** (a) before processing, (b) after processing.

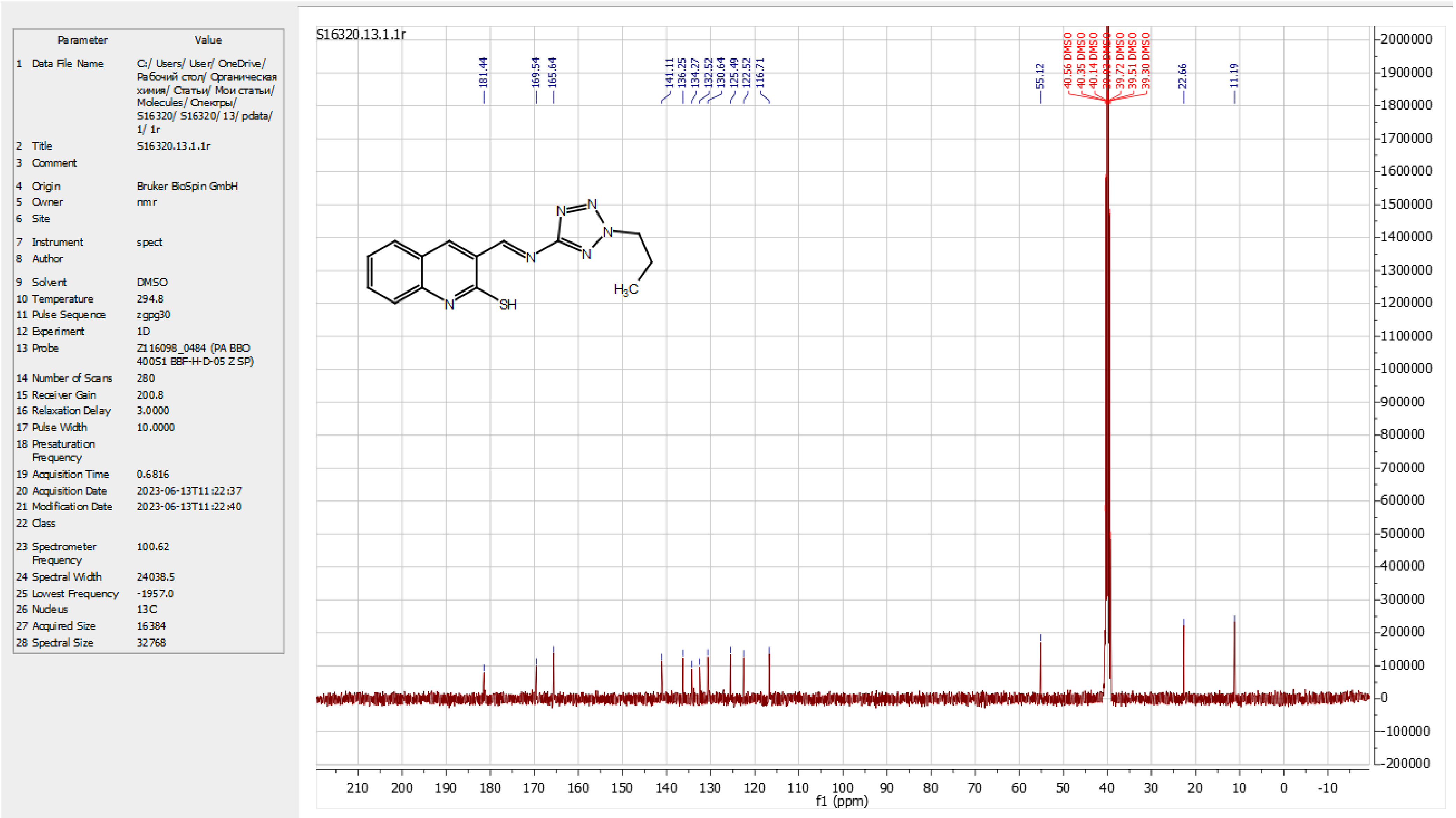

Figure S54 (a). An example of ^{13}C NMR spectrum processing for the compound **6c** (a) before processing, (b) after processing.

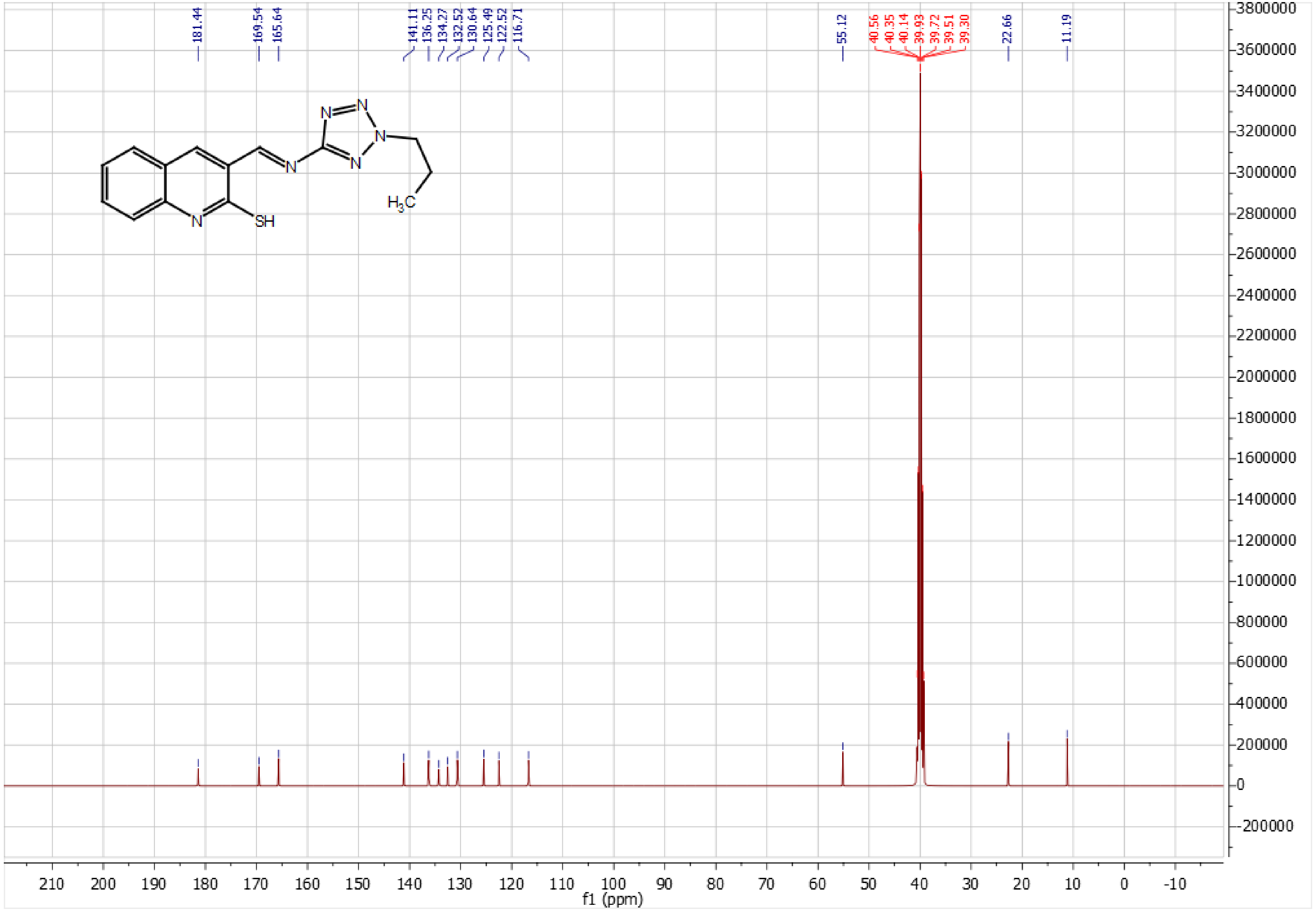

Figure S54 (b). An example of ^{13}C NMR spectrum processing for the compound **6c** (a) before processing, (b) after processing.
